# Biogenesis of Triterpene Dimers from Orthoquinones Related to Quinonemethides: Theoretical Study on the Reaction Mechanism

**DOI:** 10.3390/molecules21111551

**Published:** 2016-11-17

**Authors:** Mariana Quesadas-Rojas, Gonzalo J. Mena-Rejón, David Cáceres-Castillo, Gabriel Cuevas, Ramiro F. Quijano-Quiñones

**Affiliations:** 1 Facultad de Química, Universidad Autónoma de Yucatán, Calle 43 No. 613, Col. Inalambrica, Mérida, Yucatán, C.P. 97069, Mexico; mariana_quesadas@yahoo.com.mx (M.Q.-R.); david.caceres@correo.uady.mx (D.C.-C.); 2 Instituto de Química, Universidad Nacional Autónoma de México, Circuito Exterior, Ciudad Universitaria, Ciudad de México, C.P. 04510, Mexico; gecgb@unam.mx

**Keywords:** Celastraceae, quinonemethide, biogenesis, hetero Diels-Alder, DFT

## Abstract

The biogenetic origin of triterpene dimers from the Celastraceae family has been proposed as assisted hetero-Diels-Alder reaction (HDA). In this work, computational calculation of HDA between natural quinonemethides (tingenone and isopristimerol) and hypothetical orthoquinones has been performed at the M06-2X/6-31G(d) level of theory. We have located all the HDA transition states supporting the biogenetic route via HDA cycloadditions. We found that all reactions take place through a concerted inverse electron demand and asynchronous mechanism. The enzymatic assistance for dimer formation was analyzed in terms of the calculated transition state energy barrier.

## 1. Introduction

**Scheme 1:**
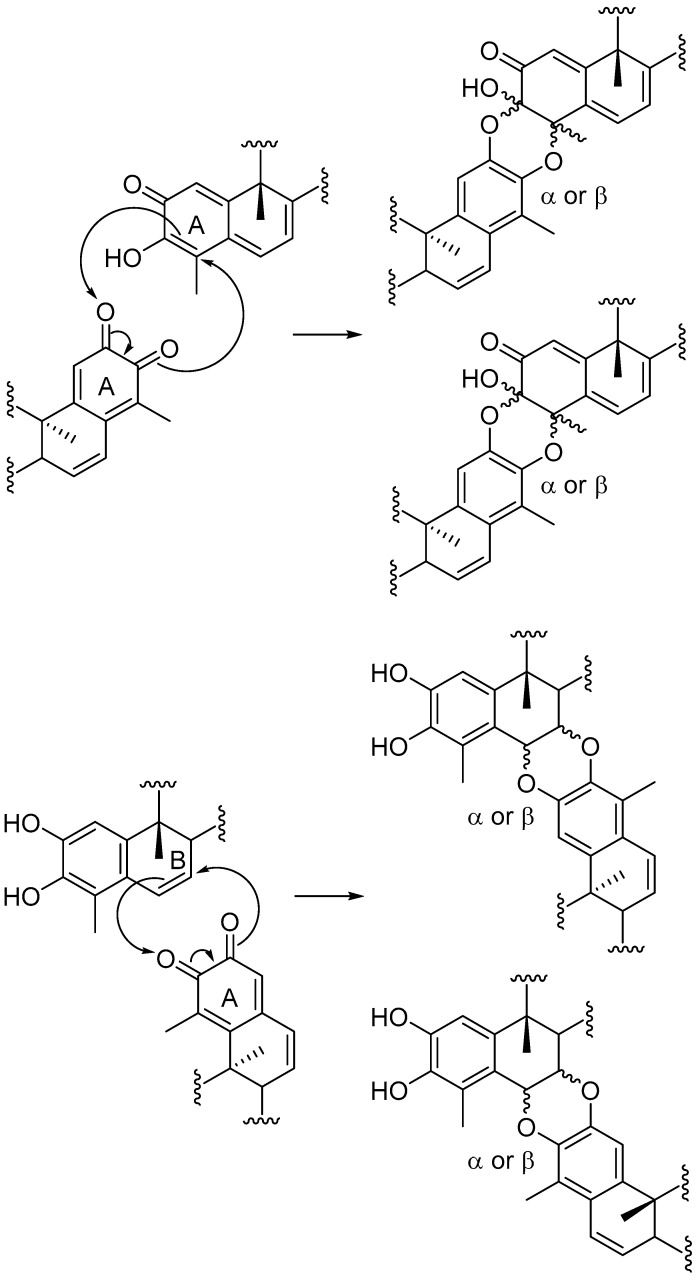
Proposed biosynthetic mechanism for formation of triterpene dimers.

Triterpenoid quinonemethides constitute a relatively small group of unsaturated and oxygenated D:A-friedo-nor-oleananes with interesting structures and a variety of biological activities [1,2,3]. These natural products are restricted to species of the Celastraceae family and, for this reason, the triterpenoid quinonemethides and related compounds (phenolic triterpenoids and triterpene dimers) are called celastroloids [4]. More than 60 triterpene dimers have been isolated in plants of the family Celastraceae to date, mainly belonging to the genus *Maytenus*: *M.* *chuchuhuasca*, *M.* *ilicifolia*, *M.* *umbellata*, *M.* *magellanica*, *M.* *scutioides*, and *M.* *blepharodes* [5,6,7,8,9,10,11,12,13]. These dimers are composed of quinonemethide and aromatic forms of nor-triterpenes, joined by two ether linkages formed between the A rings of the two celastroloids (A-A dimers), or between the A and the B rings (B-A dimers). It is noteworthy to stress that the intermediacy of orthoquinones related to quinonemethides has been implicated in the biogenesis of triterpene dimers [10,14,15,16] and recently, triterpenes with an orthoquinone system have been isolated from *Celastrus orbiculatus* [17]. The proposed biosynthetic mechanism includes a hetero Diels-Alder (HDA) type cycloaddition reaction between an orthoquinone and a quinonemethide triterpenoid to form A-A dimers, or a phenolic triterpenoid to form B-A dimers (Scheme 1).

The Diels-Alder (DA) reaction is one of the most important ring-forming reactions in organic synthesis. A large number of studies have resulted in several mechanisms that describe the Diels-Alder (DA) and HDA reactions, proposing three classes of them: non-polar, polar, and ionic [18,19,20,21,22]. The HDA and DA cycloadittions have been postulated as a key step in more than 100 biosynthetic conversions [11]. Nevertheless, until now, it has been difficult to verify the existence of a Diels-Alderase enzyme; however, cell-free extracts of the fungus *Alternaria solani* were shown to catalyze a Diels-Alder-type cycloaddition. Furthermore, a macrophomate synthase (MPS) was reported as the first example of a biological Diels-Alderase and it has been reported that lovastatin and structurally related polyketides are formed by Diels-Alderase to cyclize their backbone [23,24,25]. However, in the case of the MPS, the recent theoretical study by Jorgensen et al. [26] suggested a more energetically probable scenario involving a non-concerted Michael-aldol reaction, whose transition state (TS) was more stable than the TS model for the DA reaction. Therefore, it is probable that the MPS mechanism does not necessarily imply a concerted Diels-Alder reaction.

Experimental work carried out by Gonzalez et al. [10] demonstrates that under DA conditions is possible to obtain a synthetic triterpene dimer from the reaction of 4α-hydroxy-pristimerin and pristimerin. These results and the isolation of the different possible regio- and stereoisomers of the dimer compounds reinforce the biogenetic route via HDA cycloaddition. However, in this sense, Jacobsen et al. [27] report that the reaction of pristimerin with 2,3-dichloro-5,6-dicyano-1,4-benzoquinone (DDQ) yields xuxuarines Eα and Eβ dimers. In addition, an unusual product obtained from this reaction was identified as pristimerin dicyanophenalenedione suggesting an alternative reaction pathway for the formation of dimeric triterpenes.

Astonishingly, to our knowledge, there are no reported theoretical studies of the reaction leading to the formation of this kind of dimers. It became evident that a theoretical study of this key step would provide additional information on the biogenesis of the triterpene dimers and the feasibility of a biological enzymatic assisted Diels-Alder cycloaddition. In this work, we present the first theoretical study of the formation of the triterpene dimers. The main goal of this paper is to obtain evidence regarding the feasibility of the hetero Diels-Alder cycloaddition as a reaction mechanism of the biogenesis of triterpene dimers, and to evaluate the importance of enzymatic participation in this process.

## 2. Computational Details

Geometry optimizations of the stationary points (reactants, hetero Diels-Alder transition state structures and products) were carried out using DFT methods at the M06-2X/6-31G(d) level of theory for a 0 K gas phase. The meta-GGA M06-2X [28] functional was applied since it was constructed to treat dispersion with more accuracy than older functionals and consequently performs better with π → σ transformations in reactions such as Diels-Alder and [3 + 2] π cycloadditions [29,30,31,32]. In addition, the M06-2X functional along with the 6-31G(d) basis set was chosen because our previous work shows that this model is appropriate for describing the hetero Diels-Alder reaction between o-benzoquinone and norbornadiene [33]. Frequency calculations were performed to characterize all the stationary points at the same computational level. The reactants and products were identified from the vibrational analysis with all real frequencies, and all of the TS of hetero Diels-Alder reactions had only one imaginary frequency corresponding to a movement in the direction of the reaction coordinate. The QST2 (Schlegel´s Synchronous Transit-Guided Quasi-Newton) calculations and, in the more difficult cases, the QST3 methods were used to locate each TS between two minima [34]. Intrinsic reaction coordinate (IRC) calculations were performed to ensure that each TSs connected each reactant with its correct product through a concerted HDA reaction [35]. Zero-point vibrational energy corrections were applied without scaling for all the examined structures. Solvent effects of water were taken into account by single-point calculations of the gas phase structures using the conductor-like polarizable continuum model (CPCM) using UAKS radii [36,37,38,39].

The free energies of activation (ΔG_act_) for the reactions were estimated by subtracting the sum of free energies of isolated reactants from the free energy of the optimized TS at 298 K and 1 atm. In a similar manner, the free energies of reaction (ΔG**_rxn_**) were calculated from the differences between the free energies of products and reactants. All calculations were carried out with the Gaussian 09 suite of programs [40]. The global electrophilicity index (ω) was calculated from the chemical potential (µ) and the chemical hardness (η) using the expression ω = (µ^2^/2η) according to Parr et al. [41]. The global electrophilicity index ω measures the stabilization in energy when the system acquires an additional electronic charge, ΔN, from the environment. Using a finite difference approximation one gets µ = −(IE + EA)/2 and η = IE − EA [42], where IE and EA are the vertical ionization energy and electron affinity, respectively. Both quantities may be calculated with IE = E(N = N_0_ − 1) − E(N = N_0_) and EA = E(N = N_0_) − E(N = N_0_ + 1), where E is the energy of the structure and N_0_ is the number of electrons in the ground state of the system. Several approaches have been established for the calculations of nucleophilicity index (***N***) [43]. In this work, we have used the empirical approach of nucleophilicity proposed by Domingo et al. [44] since several studies have evidenced the capability to predict the nucleophilic behavior of organic molecules [43]. In this scheme, *N* is defined as

$$N=E_{HOMO}\left(Nucleophile\right)-E_{HOMO}\left(TCE\right)$$

where $E_{HOMO}\left(TCE\right)$ is the HOMO energy of tetracyanoethylene which is one the most electrophilic neutral species [43].

## 3. Results and Discussion

**Scheme 2:**
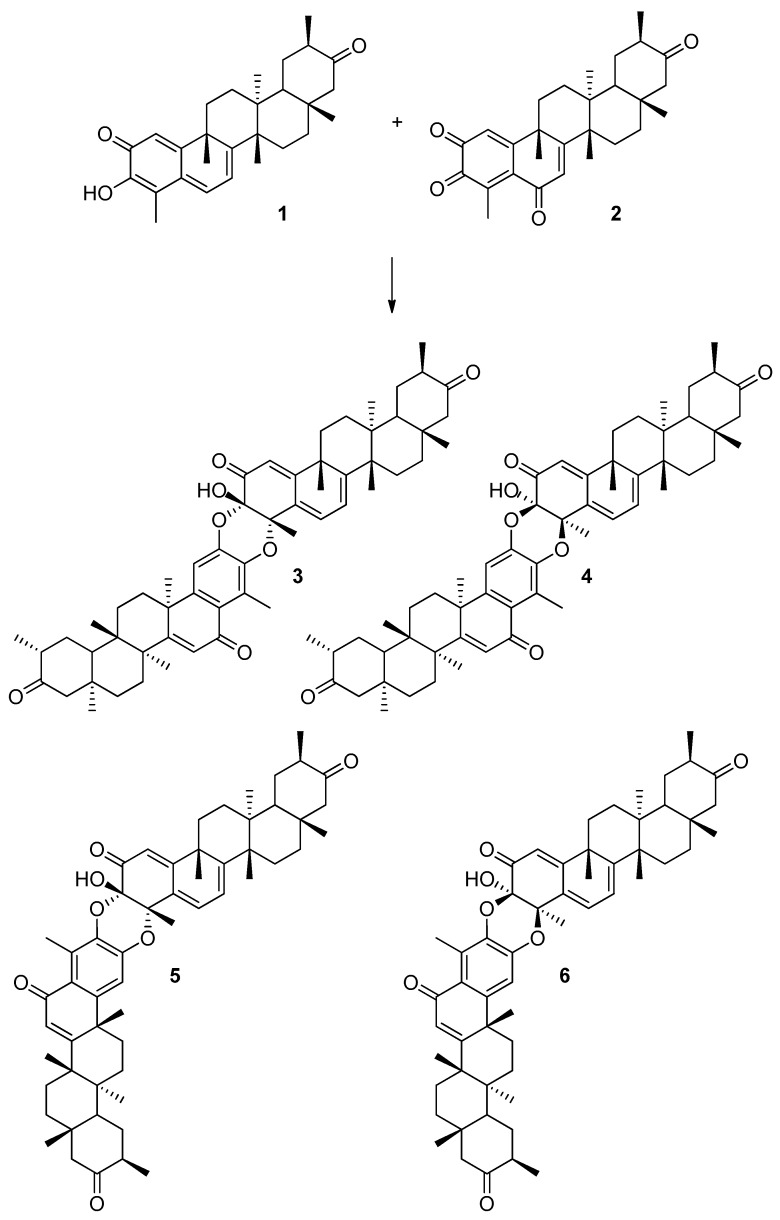
Structures of A-A dimers studied in this work (**3**–**6**).

**Scheme 3:**
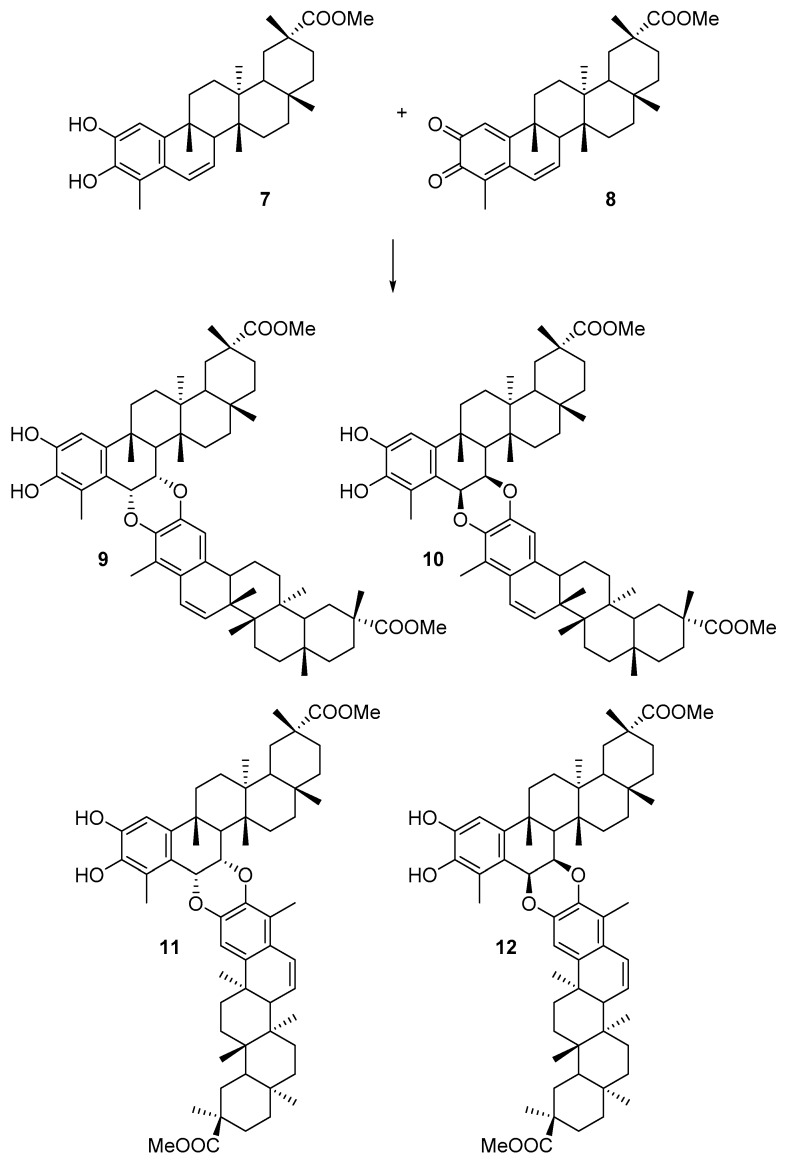
Structures of B-A dimers studied in this work (**9**–**12**).

In particular, we have analyzed the mechanism of the HDA reaction between tingenone **1** with the hypothetical orthoquinone derived from 6-oxotingenol, oq-6-oxotingenol **2**, to form four A-A dimers: xuxuarine Aα **3** [15], xuxuarine Aβ **4** [27], isoxuxuarine Aα **5** [12], and isoxuxuarine Aβ **6** [12] (Scheme 2). Additionally, we have studied the reaction between isopristimerol **7** with the hypothetical orthoquinone derived from isopristimerol, oq-isopristimerol **8**, to form four B-A dimers: cangorosin A **9** [13], cangorosin Aβ **10**, isocangorosin A **11** [13], and isocangorosin Aβ **12** (Scheme 3). It is important to note that cangorosin Aβ and isocangorosin Aβ have not been isolated yet.

For each adduct the diene and dienophile may arrange themselves in two different ways (*exo* and *endo*) giving a total of 16 possible HDA transition states. Firstly, an analysis of the global electrophilicity and nucleophilicity index [41,44,45] for the dienes and dienophiles was performed in order to gain an understanding on the reactivity of the triterpenoid quinonemethides and related compounds. Then, we investigated the TS structures for the triterpene dimerization and their mechanism was analyzed.

### 3.1. Reactivity Global Indexes

The reactivity global descriptors: electronic chemical potential (μ), global electrophilicity (ω), and global nucleophilicity (*N*) from the triterpene units of the optimized dimers studied in this work were obtained, and are listed in Table 1. From the analysis of these descriptors, we see that the ω of orthoquinones are higher than the ω of quinonemethide triterpenoids and phenolic triterpenoids and the *N* of orthoquinones are lower than the *N* of the quinone and phenolic systems. In addition, the μ of orthoquinones is lower than tingenone and isopristimerol. Therefore, in both cases, the HDA reactions may proceed through inverse electron demand (IED) and then the charge transfer (CT) of these cycloaddition reactions will take place from tingenone **1** to oq-6-oxotingenol **2** to form the A-A dimers and from isopristimerol **7** to oq-isopristemerol **8** to form the B-A dimers. These results are in agreement with those reported in previous works on orthoquinones systems [46,47,48,49]. Within the scale of electrophilicity and nucleophilicity power introduced by Domingo et al. [50] the isopristimerol is a strong nucleophile, the tingenone is a moderate nucleophile and the orthoquinones studied in this work are strong electrophiles and the latter can be classified as highly reactive molecules.

### 3.2. Frontier Orbital Analysis

**Figure 1:**
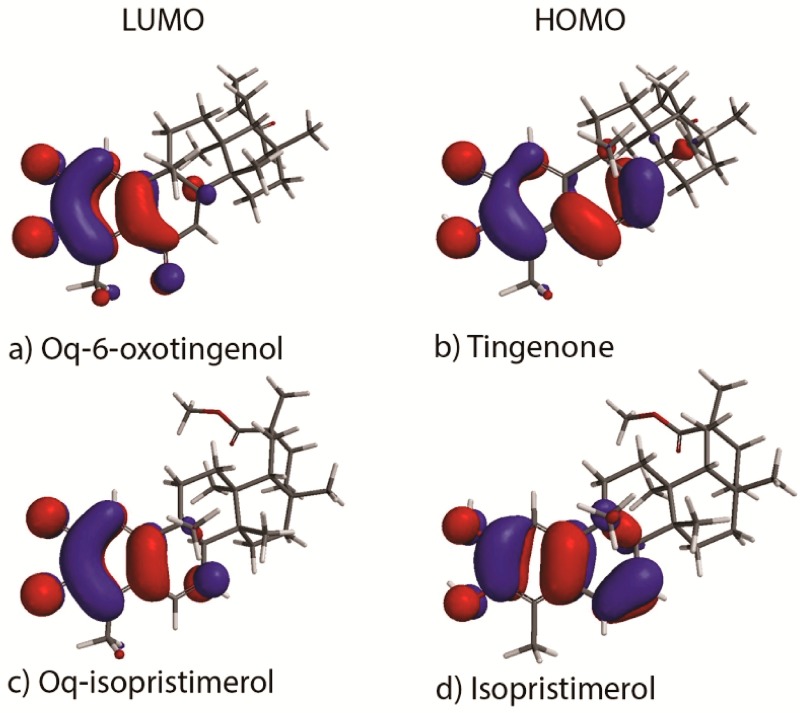
Frontier Molecular Orbitals of quinonic and phenolic triterpenoids implicated in the Hetero-Diels Alder reaction.

In order to gain understanding in analysis of the studied HDA reactions we have calculated the frontier orbitals HOMO and LUMO for the celastroloids: tingenone **1**, oq-6-oxotingenol **2**, isopristimerol **7**, oq-isopristimerol **8** (Figure 1). The HOMO and the LUMO are highly concentrated in the rings A and B, mainly due to the functional groups located in these rings. The energy differences between the frontier orbitals HOMO and LUMO for the celastroloids studied in this work are listed in Table 2. We observe that the energy gap between the LUMO of the diene and the HOMO of the dienophiles (ΔIED) has the lower value. This indicates that, LUMO(diene)–HOMO(dienophile) interactions are the important ones. This interaction corresponds to inverse electron demand HDA in agreement with the results found with the reactivity global indexes. It is important to note that the ΔIED of B-A dimers are higher than the ΔIED of A-A, suggesting that the activation energy is more accessible for the formation of the latter cycloadducts.

### 3.3. Study of the HDA Reactions

In the case of the A-A dimers the favored conformers (Figure S1 in Supplementary Materials) present a hydrogen bond between the hydroxyl group and the carbonyl in the quinonemethide. The O–H and H•••O distances fluctuate from 0.976 Å (isoxuxuarine Aα, **5**) up to 0.977 Å (xuxuarine Aβ, **4**) and from 0.055 Å (xuxuarine Aβ, **4**) up to 2.076 Å (isoxuxuarine Aα, **5**) respectively, while the O-H•••O angle varies from 115.74° (xuxuarine Aβ, **4**) down to 114.35° (isoxuxuarine Aα, **5**). In addition, in the favored B-A conformers (Figure S2 in Supplementary Materials), the rings present the individual ground state conformations (cyclohexane in the chair form, cyclohexene in the half chair conformation, etc.).

All 16 possible transition structures corresponding to each HDA reaction channel were located. These theoretical results support the hypothesis of a HDA reaction for the biogenesis of the triterpene dimers in plants of the Celastraceae family [1]. In order to explore the possible formation of the triterpene dimers via a stepwise mechanism, we searched for a non-concerted mechanism. However, all attempts to find hypothetical non-concerted pathways were unsuccessful. The Cartesian coordinates of the 16 TSs associated with the reactions studied are listed in the Supplementary Materials. The intrinsic reaction coordinate from TSs to reactants stop in charge transfer reactant complexes (CTC), which are energetically more stable than the separated reactants. However, in all cases the negative enthalpy resulting from CTC is cancelled by the entropic term associated with bringing two molecules together and, as a consequence, the free energies of complex formation are positive and the CTs do not have any significant contribution to the tested HDA reactions. The relative free energies, entropy, and enthalpy contributions for the reactions are summarized in Table 3; in this table we show the free energy change (ΔG_rxnt_) associated with the formation of the eight triterpenes dimers studied in this work. ΔG_rxn_ fluctuates from −20.9 kcal/mol (cangorosin Aβ, **10**) down to −27.5 kcal/mol (isoxuxuarine Aα, **5**). All the reactions were calculated to be exothermic. In the A-A type dimers, dimers with α stereochemistry (xuxuarine Aα, **3** and isoxuxuarine Aα, **5**) are more stable than the dimers with β stereochemistry (xuxuarine Aβ, **4** and isoxuxuarine Aβ, **6**) by 1.7–2.6 kcal/mol. For dimers B-A, the dimers with α stereochemistry are favored over dimers with β stereochemistry by 2.2–2.4 kcal/mol. These results could explain why **4** and **6** are not isolated yet. In all cases, the unfavorable reaction entropies are close to 16.7 kcal/mol.

The 16 activation free energies (ΔG_act_) for the reaction mechanism of the eight triterpene dimers studied in this work were collected in Table 3. In general, the ΔG_act_ for A-A dimers are lower than ΔG_act_ for B-A dimers in agreement with the results found in the frontier orbital analysis and with the fact that oq-6-oxotingenol **2** (*ω* = 2.084) is more electrophilic than the oq-isopristimerol **8** (*ω* = 1.707). In addition, the ΔG_act_ fluctuates from 25.5 (isoxuxuarine Aα, **5** via *endo*) up to 41.4 kcal/mol (isocangorosin Aβ, **12** via *endo*). Although evidence on natural Diels-Alderases has been accumulated [23,24,25], there is not enough information about the thermodynamics of this process. In this sense, the computational studies are valuable. The free energy barriers for A-A triterpene dimers formation are predicted to be 25.3–30.4 kcal/mol. Most of the chemical reactions occurring in the metabolic process of living cells have an activation barrier of 15.1–25.1 kcal/mol [51,52,53]. Therefore, a free energy barrier higher than 25.1 kcal/mol is considered high for thermal energy, indicating that the reaction will be slow at ambient temperature and tends to argue for an enzymatic reaction [22,51,52,53]. In addition, usually the M06-2X DFT method underestimates reaction barrier heights [22]. In order to test the accuracy of the free activation energies predicted here, the free energy of activation for the simple HDA reaction between the o-benzoquinone and the vinyl alcohol, which is a simple HDA reaction similar to those studied in this work, was calculated using the M06-2X/6-31G(d) and G3B3 models. The G3B3 [54] is a high-level composite method that combines a series of well-defined ab initio calculations to reach an energy at QCISD(T)/(T,full)/G3large level. The G3B3 total energy calculation is computationally demanding and thus their routine application is limited to small systems. However, it is a common practice to use G3B3 values whenever experimental data are unavailable [55], as in the present case. The computed G3B3 free activation energies for *endo* and *exo* channels are 30.9 and 32.6 kcal/mol, respectively. The results for M06-2X/6-31G(d) are 30.1 (*endo* channel) and 32.2 (*exo* channel) kcal/mol and are close to G3B3 results showing that M06-2X/6-31G(d) is an appropriate level for modeling the HDA reaction studied here. In addition, as we expected, the DFT method underestimates the free reaction barrier heights. The energy requirements for the HDAs for the first set of dimers are within the ranges of the conditions employed during the isolation of the natural products (hot methanol). However, due to Zhao et al. [17] reporting about triterpene dimers and orthoquinone system isolation at room temperature and the low percent yield reported in the biomimetic synthesis of triterpene dimers [10,27], it seems unlikely that dimers were an artifact formed during the extraction procedure.

The computed free energy barriers discussed in this work are for gas-phase cycloaddition (a crude model for the reaction in a non-polar enzyme active site) and the most biological environments are likely to be polar. In order to estimate the water effects in the activation energy, the effect of the solvent in water were considered implicitly. This was done by performing single-point energy calculations on the gas-phase geometries of the stationary points of all A-A dimers in the *endo* and *exo* channel, through the conductor-like polarizable continuum model (CPCM) [36,37,38,39], using the UAKS radii. In all the cases, the calculation predicted higher energy barriers than observed in gas phase. The difference of the activation energy between the water and the gas phase fluctuates from 3.6 kcal/mol (xuxuarine Aα, *endo*) down to 1.4 kcal/mol (isoxuxuarine Aβ, *exo*). It is well known that the acceleration of the Diels-Alder reaction by water is a general phenomenon [56,57]. However, our solvent phase calculations do not agree with this fact. When substances with non polar regions are dissolved in water, they tend to associate so as to diminish the hydrocarbon-water interfacial area. Hence in the TS for HDA reactions, the two hydrocarbon surfaces must come together and one might expect that the HDA reaction operating in the formation of the dimers of triterpenes could be accelerated [52,57,58]. Therefore, from a theoretical point of view, it is not possible to conclude that an enzyme may be responsible for the A-A dimers formation.

In the second set of dimers, the B-A dimers, the free energies activations are predicted to be 36.0–37.8 kcal/mol, which is quite high for ambient temperature. Interestingly, the two as yet unknown isomers, while having significantly higher free activation energies via the *endo* channel, have comparable free activation energies via *exo* addition. Due to the free activation energies being considerably higher than 25.1 kcal/mol, they seem more likely to require enzymatic assistance [51,52,53]. The enzyme can work in two ways; either stabilizing the TS or destabilizing the ground states energy of the reactants. In the suggested MPS mechanism, two hydrogen bonds and the appropriate orientation of the 2-pyrone and the enolate maximize the overlapping of π-orbital in the dienophile and the diene in the TS [59].

In the case of A-A dimers, the difference between the *exo* TS and *endo* TS activation enthalpies (ΔΔH_act_) varies from 1.4 (isoxuxuarine Aβ, **6**) to 9.4 kcal/mol (isoxuxuarine Aα, **5**). However, as a consequence of the *endo* TS is a more compact form than the *exo* TS; the unfavorable activation entropic term in the *endo* TS is greater than in the *exo* TS. This decreases the activation free energy differences between the *exo* and *endo* transition states. However, the *endo* TS remains favored over the *exo* TS, except for isoxuxuarine Aβ **6**, where the activation free energy is similar (ΔΔG_act_ = 0.45 kcal/mol). The *endo* preference may be due to the favorable interaction between the secondary orbitals via the *endo* approach and for other interactions like van der Waals attractions [60,61,62]. It is interesting to note that, in the B-A dimers, the *endo* TSs is favored over the *exo* TSs in the α stereochemistry (isocangorosin A, **11** and cangorosin A, **9**), while in the β stereochemistry the *exo* route has the lowest activation free energy. Similarly to A-A dimers, the unfavorable activation entropic term in *endo* TS is greater than in the *exo* TS, except for cangorosin Aβ **10** where the value of the entropic term is similar.

### 3.4. Geometrical and Charge Transfer Analysis

**Figure 2:**
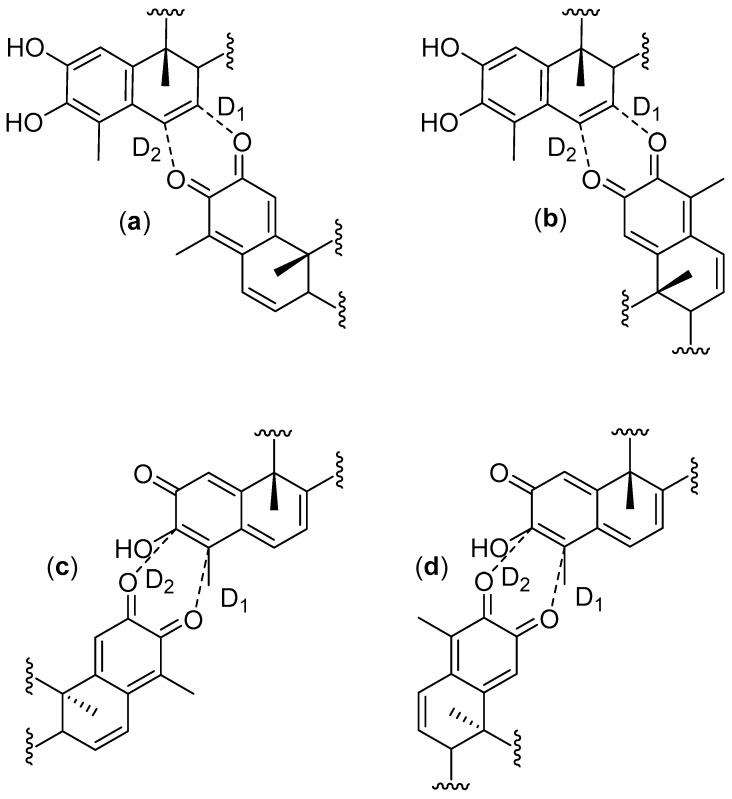
Nomenclature for selected bond lengths in the transition states for cangorosines A and Aβ (**a**); isocangorosines A and Aβ (**b**); xuxuarines Aα and Aβ (**c**); isoxuxuarines Aα and Aβ (**d**).

In order to get deeper insights into the nature of this HDA reaction mechanism, we have calculated the charge transfer (CT), the distance of the selected bond (D) (Figure 2) and the asynchronicity degrees (AD) [63] for the TSs studied in this work. The selected parameters for the TSs are shown in Table 4.

**Figure 3:**
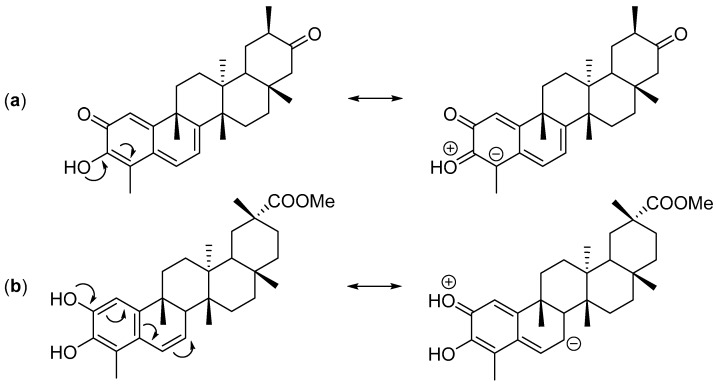
Mesomeric structures of tingenone (**a**) and isopristimerol (**b**).

The CT calculated using the Mulliken population analysis [64], fluctuates from 0.297 e (*exo* TS cangorosin Aβ, **10**) up to 0.418 e (*endo* TS isoxuxuarine Aα, **5**). Interestingly, the CTs in A-A dimers are always higher than the CT in B-A dimers. These results are in agreement with the values obtained in the analysis of the DFT based reactivity indices and suggests that the hydroxyl group of tingenone **1** has a higher electron donating power than the phenolic ring of the isopristimerol **7**. The values of asynchronicity degree vary from 0.02 (*endo* TS cangorosin A, **9**) to 0.21 (*endo* TS xuxuarine Aβ, **4**). Interestingly, the *endo* TSs have higher values of synchronicity than the *exo* TSs. From bond distances listed in Table 4, we observe that for the *exo* TSs of A-A dimers, the length of the D1 bond distance are shorter than the length of the D2. This is because the π donor character of the hydroxyl group at position 3 (Figure 3a) allows the charge transfer from the quinonemethide to the orthoquinone causing a decrease in the C4-O bond length. In the case of *exo* TSs of A-B dimers, the length of the D1 bond is shorter than the length of the D2 bond, due to π donating power of the phenolic ring in the dienophile (Figure 3b). These results indicate that these HDA cycloaddition reactions proceeded via a polar asynchronous mechanism [65].

## 4. Conclusions

In summary, we have analyzed the feasibility of the hetero Diels-Alder cycloaddition on the biosynthetic mechanism of triterpene dimers of Celastraceae plant family using the DFT method at the M06-2X/6-31G(d) computational level. We have located all the HDA transition states and the mechanism of the reaction and its stereoselectivity were fully analyzed (*endo*/*exo* and specific diastereofacial stereochemistry of adducts). We conclude that these reactions take place through a concerted and asynchronous mechanism. The electrophilicity index of orthoquinones studied in this work classifies them as highly reactive molecules. In all the reactions studied, the HDA reactions may proceed through inverse electron demand (IED). The plausible enzymatic participation was examined in terms of the calculated transition state free energy barrier.

## Figures and Tables

**Table 1:** Computed global reactivity indexes of dienes and dienophiles.

Molecule	µ (eV)	ω (eV)	N (eV)
tingenone **1**	−4.218	1.409	−6.742
oq-6-oxotingenol **2**	−5.415	2.084	−8.330
isopristimerol **7**	−3.020	0.551	−6.460
oq-isopristimerol **8**	−4.816	1.707	−7.590

**Table 2:** Calculated energy HOMO-LUMO GAP (eV) between dienes and dienophiles for A-A and B-A type dimers.

Diene	Dienophile	ΔNED ^a^	ΔIED ^b^
oq-6-oxotingenol **2**	tingenone **1**	6.626	4.100
oq-isopristimerol **8**	isopristimerol **7**	8.019	4.326

^a^ LUMO_dienophile_ − HOMO_diene_; ^b^ LUMO_diene_ − HOMO_dienophile_.

**Table 3:** Relative enthalpies, entropies, and Gibbs energies (kcal/mol) for the stationary points involved in the DA reactions at 298 K and 1 atm.

Molecule	ΔG_rxn_	ΔH_rxn_	TΔS_rxn_	*endo*			*exo*		
				ΔG_act_	ΔH_act_	TΔS_act_	ΔG_act_	ΔH_act_	TΔS_act_
Xuxuarine Aα **3**	−27.1	−44.4	−17.3	27.6	8.8	−18.8	30.6	14.1	−16.4
Xuxuarine Aβ **4**	−24.3	−42.1	−17.7	28.1	11.2	−16.8	30.6	13.8	−16.8
Isoxuxuarine Aα **5**	−27.5	−43.7	−16.1	25.5	5.2	−20.2	30.9	14.6	−16.2
Isoxuxuarine Aβ **6**	−25.8	−42.1	−16.3	29.7	11.9	−17.8	29.3	13.3	−16.0
Cangorosin A **9**	−23.1	−39.2	−16.1	36.0	16.7	−19.3	38.7	23.1	−15.6
Cangorosin Aβ **10**	−20.9	−37.4	−16.5	40.0	25.0	−15.0	37.8	21.9	−16.0
Isocangorosin A **11**	−23.4	−39.2	−15.9	36.8	18.7	−18.1	38.5	23.3	−15.2
Isocangorosin Aβ **12**	−21.0	−37.6	−16.6	41.4	22.0	−19.3	36.2	21.4	−14.9

**Table 4:** Theoretical reactivity parameters, asynchronicity degree (AD), charge transfer (CT, in e), and selected bond distances (D, in Å), for *exo* and *endo* transition states (TS) and products.

Molecule	*exo* TS				*endo* TS				Products ^a^	
	AD	CT ^b^	D1	D2	AD	CT ^b^	D1	D2	D1	D2
Xuxuarine Aα **3**	0.19	0.397	1.92	2.20	0.06	0.357	2.02	2.11	1.42	1.43
Xuxuarine Aβ **4**	0.21	0.404	1.90	2.22	0.07	0.366	2.01	2.12	1.42	1.43
Isoxuxuarine Aα **5**	0.10	0.382	1.98	2.12	0.15	0.418	2.16	1.94	1.42	1.42
Isoxuxuarine Aβ **6**	0.09	0.381	1.98	2.11	0.07	0.378	2.11	2.02	1.42	1.42
Cangorosin A **9**	0.16	0.319	1.92	2.16	0.02	0.307	2.01	2.05	1.42	1.43
Cangorosin Aβ **10**	0.16	0.297	1.97	2.20	0.03	0.317	2.03	2.07	1.42	1.42
Isocangorosin A **11**	0.06	0.310	1.98	2.08	0.16	0.329	2.11	1.89	1.42	1.43
Isocangorosin Aβ **12**	0.19	0.300	1.96	2.23	0.03	0.308	2.07	2.02	1.42	1.42

^a^ Corresponding to dimers suggested in Figure 2; ^b^ CT was calculated using Mullliken’s partition scheme.

## Supplementary Materials

Supplementary materials can be accessed at: http://www.mdpi.com/1420-3049/21/11/1551/s1.

## References

[1] Zhou Q., Rokita S.E. (2009). Natural Diterpene and Triterpene quinone methides: Structures, synthesis, and biological potentials. Quinone Methides.

[2] Gunatilaka A.A.L., Dhanabalasingham B., Karunaratne V., Kikuchi T., Tezuka Y. (1993). Studies on terpenoids and stereoids. Part 27. Structure of a D:A-friedo-oleanane triterpenoid from *Salacia reticulata* and revision of the structures of kokoonol and kokzeylanol series of triterpenoids. Tetrahedron.

[3] Furbacher T.R., Gunatilaka A.A.L. (2001). Catalytic inhibition of topoisomerase IIα by demethylzeylasterone, a 6-oxophenolic triterpenoid from *Kokoona zeylanica*. J. Nat. Prod..

[4] Gunatilaka A.A.L. (1996). Triterpenoid Quinonemethides and Related Compounds (Celastroloids). Progress in the Chemistry of Organic Natural Products.

[5] González A.G., Rodríguez F.M., Bazzocchi I.B., Ravelo A.G. (2000). New terpenoids from *Maytenus blepharodes*. J. Nat. Prod..

[6] Shirota O., Sekita S., Satake M. (2004). Nine New isoxuxuarinse-type triterpene dimers from *Maytenus chuchuhuasca*. Chem. Biodivers..

[7] Shirota O., Morita H., Takeya K., Itokawa H. (1998). New geometric and stereoisomeric triterpene dimers from *Maytenus chuchuhuasca*. Chem. Pharm. Bull..

[8] Shirota O., Sekita S., Satake M. (2004). Nine triterpene dimers from *Maytenus chuchuhuasca*. Helv. Chim. Acta.

[9] González A.G., Kennedy M.L., Rodríguez F.M., Bazzocchi I.L., Jiménez I.A., Ravelo A.G., Moujir L. (2001). Absolute configuration of triterpene dimers from *Maytenus* species (Celastraceae). Tetrahedron.

[10] Gonzalez A.G., Alvarenga N.L., Estevez-Braun A., Ravelo A.G., Bazzocchi I.L., Moujir L. (1996). Structure and absolute configuration of triterpene dimers from *Maytenus scutioides*. Tetrahedron.

[11] Shirota O., Sekita S., Satake M., Morita H., Takeya K., Itokawa H. (2004). Nine regioisomeric and stereoisomeric triterpene dimers from *Maytenus chuchuhuasca*. Chem. Pharm. Bull..

[12] Shirota O., Morita H., Takeya K., Itokawa H. (1997). Five new triterpene dimers from *Maytenus chuchuhuasca*. J. Nat. Prod..

[13] Shirota O., Morita H., Takeya K., Itokawa H. (1997). Revised structures of cangorosins, triterpene dimers from *Maytenus ilicifolia*. J. Nat. Prod..

[14] Minami A., Oikawa H., Poupon E., Nay B. (2011). The diels-alderase never ending story. Biomimetic Organic Synthesis.

[15] Shirota O., Morita H., Takeya K., Itokawa H. (1995). Structures of xuxuarines, stereoisomeric triterpene dimers from *Maytenus chuchuhuasca*. Tetrahedron.

[16] Gonzalez A.G., Alvarenga N.L., Bazzocchi I.L., Ravelo A.G., Moujir L. (1999). Triterpene trimers from *Maytenus scutioides*: Cycloaddition compounds?. J. Nat. Prod..

[17] Wu J., Zhou Y., Wang L., Zuo J., Zhao W. (2012). Terpenoids from root bark of *Celastrus orbiculatus*. Phytochemistry.

[18] Jasiński R. (2016). A reexamination of the molecular mechanism of the Diels-Alder reaction between tetrafluoroethene and cyclopentadiene. React. Kinet. Mech. Catal..

[19] Singleton D.A., Schulmeier B.E., Hang C., Thomas A.A., Leung S.W., Merrigan S.R. (2001). Isotope effects and the distinction between synchronous, asynchronous, and stepwise Diels-Alder reactions. Tetrahedron.

[20] Firestones R.A. (1996). Volume of concert and heavy atom effects in Diels-Alder reaction mechanisms. Tetrahedron.

[21] Jasinski R., Kubik M., Krygier A.L., Kacka A., Dresler E., Czubara A.B. (2014). An experimental and theoretical study of the hetero Diels-Alder reactions between (E)-2-aryl-1-cyano-1-nitroethenes and ethyl vinyl ether: One-step or zwitterionic, two-step mechanism?. React. Kinet. Mech. Catal..

[22] Jasinski R. (2014). Searching for zwitterionic intermediates in Hetero Diels-Alder reactions between methyl α,p-dinitrocinnamate and vinyl-alkyl ethers. Comput. Theor. Chem..

[23] Katayama K., Kobayashi T., Oikawa H., Honna M., Ichihara A. (1998). Enzymatic activity and partial purification of solanapyrone synthase: first enzyme catalyzing Diels-Alder reaction. Biochim. Biophys. Acta.

[24] Pohnert G. (2003). Macrophomate synthase: The first structure of a natural diels-alderase. ChemBioChem.

[25] Campbell C.D., Vederas J.C. (2010). Biosynthesis of lovastatin and related metabolites formed by fungal iterative PKS enzymes. Biopolymers.

[26] Guimarães C.R.W., Udier-Blagović M., Jorgensen W.L. (2005). Macrophomate synthase: QM/MM simulations address the Diels-Alder versus Michael–Aldol reaction mechanism. J. Am. Chem. Soc..

[27] Jacobsen N.E., Wijeratne K., Corsino J., Furlan M., Bolzani V., Gunatilaka A.A.L. (2008). Biomimetic synthesis of xuxuarines Eα and Eβ: Structure revision of *Rzedowskia* bistriterpenoids. Bioorg. Med. Chem..

[28] Zhao Y., Truhlar D.G. (2008). Density functionals with broad applicability in chemistry. Acc. Chem. Res..

[29] Pieniazek S., Clemente F., Houk K. (2008). Sources of error in DFT computations of C–C bond formation thermochemistries: π→σ transformations and error cancellation by DFT methods. Angew. Chem. Int. Ed..

[30] Hohenstein E.G., Chill S.T., Sherrill C.D. (2008). Assessment of the performance of the M05−2X and M06−2X exchange-correlation functionals for noncovalent interactions in biomolecules. J. Chem. Theory Comput..

[31] Linder M., Brinck T. (2012). Stepwise Diels–Alder: More than just an oddity? A computational mechanistic study. J. Org. Chem..

[32] Tajabadi J., Bakavoli M., Gholizadeh M., Eshghi H. (2016). A mechanistic insight into the effect of piperidine as an organocatalyst on the [3 + 2] cycloaddition reaction of benzalacetone with phenyl azide from a computational study. Org. Biomol. Chem..

[33] Quijano-Quiñones R.F., Quesadas-Rojas M., Cuevas G., Mena-Rejón G.J. (2013). Theoretical study of the Diels-Alder reaction between o-benzoquinone and norbornadiene. IOP Conf. Ser. Mater. Sci. Eng..

[34] Peng C., Ayala P.Y., Schlegel H.B., Frisch M.J. (1996). Using redundant internal coordinates to optimize equilibrium geometries and transition states. J. Comput. Chem..

[35] Fukui K. (1981). The path of chemical reactions-the IRC approach. Acc. Chem. Res..

[36] Klamt A., Schüürmann G. (1993). COSMO: A new approach to dielectric screening in solvents with explicit expressions for the screening energy and its gradient. J. Chem. Soc. Perkin Trans. 2.

[37] Andzelm J., Kölmel C., Klamt A. (1995). Incorporation of solvent effects into density functional calculations of molecular energies and geometries. J. Chem. Phys..

[38] Barone V., Cossi M. (1998). Quantum calculation of molecular energies and energy gradients in solution by a conductor solvent model. J. Phys. Chem. A.

[39] Cossi M., Rega N., Scalmani G., Barone V. (2003). Energies, structures, and electronic properties of molecules in solution with the C-PCM solvation model. J. Comput. Chem..

[40] Frisch M.J., Trucks G.W., Schlegel H.B., Scuseria G.E., Robb M.A., Cheeseman J.R., Scalmani G., Barone V., Mennucci B., Petersson G.A. (2009). Gaussian 09.

[41] Parr R.G., von Szentpaly L., Liu S. (1999). Electrophilicity index. J. Am. Chem. Soc..

[42] Chermette H. (1999). Chemical reactivity indexes in density functional theory. J. Comp. Chem..

[43] Domingo L.R., Gutierrea M.R., Pérez P. (2016). Applications of the Conceptual Density Functional Theory Indices to Organic Chemistry Reactivity. Molecules.

[44] Domingo L.R., Chamorro E., Pérez P. (2008). Understanding the reactivity of captodative ethylenes in polar cycloaddition reactions. A theoretical study. J. Org. Chem..

[45] Geerlings P., De Proft F.D., Langenaeker W. (2003). Conceptual density functional theory. Chem. Rev..

[46] Lemal D.M., Ramanathan S., Shellito J. (2008). *o*-Fluoranil chemistry: Diels-Alder versus hetero-Diels-Alder cycloaddition. J. Org. Chem..

[47] Lemal D.M., Ramanathan S. (2009). *o*-Fluoranil: Stereochemistry and mechanism of its Diels-Alder reactions. J. Org. Chem..

[48] Margetic D., Johnston M.R., Warrener R.N. (2000). High-level computational study of the site-, facial- and stereoselectivities for the Diels-Alder reaction between o-benzoquinone and norbornadiene. Molecules.

[49] Rosokha S.V., Korotchenko V., Stern C.L., Zaitsev V., Ritzert J.T. (2012). Substituent-induced switch of the role of charge-transfer complexes in the Diels-Alder reactions of *o*-chloranil and styrenes. J. Org. Chem..

[50] Domingo L., Aurell M.J., Pérez P., Contreras R. (2002). Quantitative characterization of the global electrophilicity power of common diene/dienophile pairs in Diels-Alder reactions. Tetrahedron.

[51] Painter P.P., Pemberton R.P., Wong B.M., Ho K.C., Tantillo D.J. (2014). The Viability of nitrone–alkene (3 + 2) cycloadditions in alkaloid biosynthesis. J. Org. Chem..

[52] Wang S.C., Tantillo D.J. (2008). Theoretical studies on synthetic and biosynthetic oxidopyrylium-alkene cycloadditions: Pericyclic pathways to intricarene. J. Org. Chem..

[53] Masel R.I. (2001). Chemical Kinetics and Catalysis.

[54] Baboul A.G., Curtiss L.A., Redfern P.C., Raghavachari K. (1999). Gaussian-3 theory using density functional geometries and zero-point energies. J. Chem. Phys..

[55] Lau Y., Zou L., Houk K.N. (2011). Computational methods to calculate accurate activation and reaction energies of 1,3-dipolar cycloadditions of 24 1,3-dipoles. J. Chem. Phys. A.

[56] Grieco P.A., Garner P., He Z. (1983). “Micellar” catalysis in the aqueous intermolecular Diels-Alder reaction: Rate acceleration and enhanced selectivity. Tetrahedron Lett..

[57] Rideout D., Breslow R. (1980). Hydrophobic acceleration of Diels-Alder reactions. J. Am. Chem. Soc..

[58] Reichardt C. (2003). Solvents and Solvent Effects in Organic Chemistry.

[59] Ose T., Watanabe K., Mie T., Honma M., Watanabe H., Yao M., Oikawa H., Tanaka I. (2003). Insight into a natural Diels-Alder reaction from the structure of macrophomate synthase. Nature.

[60] Wannere C.S., Paul A., Herges R., Houk K.N., Schaefer H.F., von Ragué Schleyer P. (2007). The existence of secondary orbital interactions. J. Comput. Chem..

[61] Ogawa A., Fujimoto H. (2002). Reexamination of orbital interactions in Diels-Alder reactions. Tetrahedron Lett..

[62] García J.I., Mayoral J.A., Salvatella L. (2005). The source of the endo rule in the Diels-Alder reaction: Are secondary orbital interactions really necessary?. Eur. J. Org. Chem..

[63] Contini S., Leone S., Menichetti S., Viglianisi C., Trimarco P. (2006). [2 + 4] and [4 + 2] cycloadditions of *o*-thioquinones with 1, 3-dienes: A computational study. J. Org. Chem..

[64] Mulliken R.S. (1955). Electronic population analysis on LCAO–MO molecular wave functions. I. J. Chem. Phys..

[65] Domingo L.R., Saéz J. (2009). Understanding the mechanism of polar Diels-Alder reactions. Org. Biomol. Chem..

