# Supplementary material for: Biogenesis of Triterpene Dimers from Orthoquinones Related to Quinonemethides: Theoretical Study on the Reaction Mechanism
Source: Molecules. 2016 Nov 17;21(11):1551. doi: 10.3390/molecules21111551 (PMC6273858; doi:10.3390/molecules21111551)
Supplement: Supplementary file 1 [file molecules-21-01551-s001.pdf]

Supplementary Materials: Biogenesis of Triterpene Dimers from Orthoquinones Related to Quinonemethides: Theoretical Study on the Reaction Mechanism

Mariana Quesadas-Rojas, Gonzalo J. Mena-Rejón, David Cáceres-Castillo, Gabriel Cuevas and Ramiro F. Quijano-Quiñones

1. Supplemental Figures

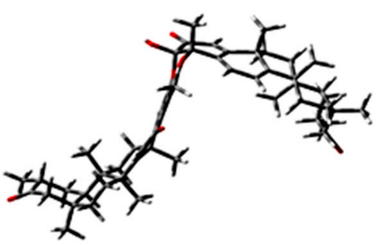

(**3**)

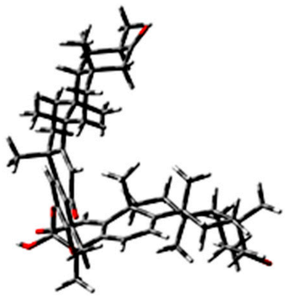

(**4**)

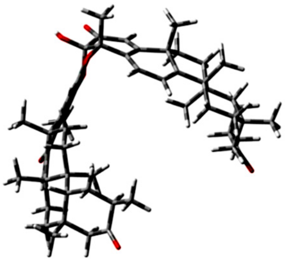

(**5**)

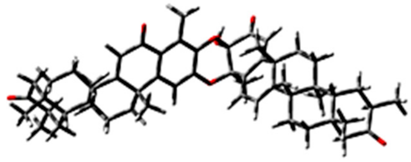

(**6**)

Figure S1. M06-2X/6-31G(d) optimized geometries of xuxuarine Aα (**3**); xuxuarine Aβ (**4**); isoxuxuarine Aα (**5**) and isoxuxuarine Aβ (**6**).

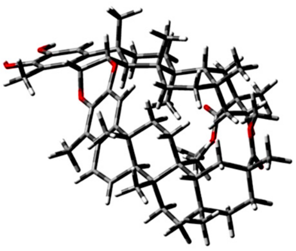

(**9**)

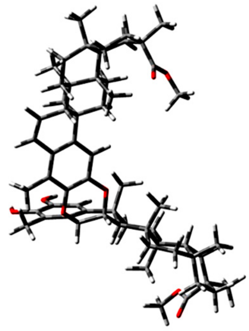

(**10**)

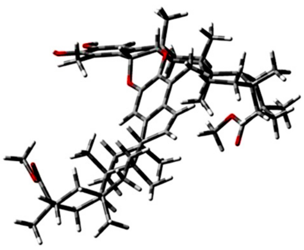

(**11**)

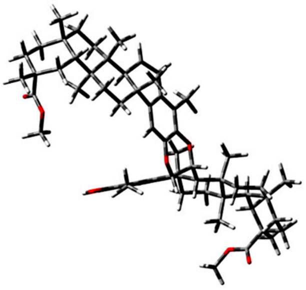

(**12**)

Figure S2. M06-2X/6-31G(d) optimized geometries of cangorosin A (**9**); cangorosin Aβ (**10**); isocangorosin A (**11**) and isocangorosin Aβ (**12**).

2. Cartesian Coordinates of All the Stationary Points for Triterpenes Included in This Study at M06-2X/6-31G* Level of Theory.

Isopristimerol

H	−3.105700	2.189300	1.243200
C	−3.693200	1.456900	0.694500
C	−5.274300	−0.385500	−0.713900
C	−3.186100	0.184100	0.435000
C	−4.958600	1.807300	0.251900
C	−5.752100	0.893400	−0.447400
C	−3.985700	−0.738300	−0.265500
O	−5.532800	3.039000	0.447600
H	−4.919400	3.609300	0.930000
O	−6.992300	1.268000	−0.871400
H	−7.127600	2.187800	−0.594100
C	−6.139300	−1.351100	−1.483400
H	−6.420100	−2.211500	−0.865500
H	−5.611300	−1.735900	−2.361600
H	−7.055300	−0.863400	−1.817600
C	−1.835000	−0.276000	0.997000
C	−3.447000	−2.082600	−0.514400
H	−4.135600	−2.877700	−0.785600
C	−2.134100	−2.319200	−0.419700
H	−1.744300	−3.315600	−0.609400
C	−1.195100	−1.180900	−0.096900
H	−1.204000	−0.552800	−1.002000
C	−0.890900	0.904800	1.278000
H	−1.253400	1.465300	2.149600
H	−0.917800	1.600800	0.429800
C	0.283000	−1.600300	0.130500
C	0.559800	0.482700	1.540000
H	0.625400	−0.086300	2.476100
H	1.143800	1.395900	1.700500
C	1.171500	−0.322900	0.372500
C	−2.157900	−0.988000	2.330400
H	−2.814900	−0.340900	2.920500
H	−1.267800	−1.185100	2.930500
H	−2.679000	−1.934600	2.163200
C	0.824700	−2.281600	−1.140400
H	0.740800	−1.587300	−1.984100

H	0.210100	−3.149400	−1.407000
C	2.613800	−0.802700	0.736700
H	2.462800	−1.369200	1.665000
C	2.268800	−2.767900	−0.970400
H	2.243800	−3.714700	−0.420200
H	2.673900	−3.027000	−1.956900
C	3.278500	−1.813100	−0.266600
C	0.365500	−2.606800	1.301100
H	0.326000	−2.127500	2.279100
H	1.284400	−3.195100	1.281600
H	−0.470800	−3.312300	1.253500
C	1.173300	0.612400	−0.854900
H	0.181700	0.735400	−1.294300
H	1.833900	0.283100	−1.654500
H	1.496800	1.608100	−0.542000
C	3.624100	0.298400	1.165000
H	3.114600	1.098500	1.709100
H	4.297200	−0.163500	1.898700
C	4.113900	−1.098000	−1.354900
H	3.471100	−0.562900	−2.060200
H	4.623500	−1.872800	−1.942500
C	4.564000	0.935000	0.115100
C	5.160500	−0.134000	−0.804000
H	5.687000	0.353600	−1.630500
H	5.912800	−0.690400	−0.231500
C	4.235100	−2.713300	0.540700
H	3.682000	−3.281400	1.297600
H	5.016200	−2.149500	1.058400
H	4.728600	−3.433100	−0.123400
C	5.698300	1.663200	0.864600
H	6.282000	0.934800	1.436600
H	5.299700	2.411700	1.554400
H	6.373600	2.162300	0.160800
C	3.891500	2.007700	−0.731800
O	3.902500	2.069400	−1.936900
O	3.324600	2.965800	0.033600
C	2.752400	4.049900	−0.694800
H	3.513300	4.541500	−1.305100
H	2.355400	4.735300	0.052700
H	1.954700	3.690400	−1.350600

M06-2X/6-31G(d) Free Energy = −1468.145124

Tingenone

C	−3.495500	1.424100	0.656100
C	−5.486800	0.664400	−0.670200
C	−3.514800	−0.718300	−0.504200
C	−4.873200	−0.496700	−0.987900
C	−2.861000	0.263500	0.364400
C	−4.831200	1.704900	0.151400
H	−3.066000	2.189800	1.292800
O	−5.450800	2.744100	0.375600
C	−2.783600	−1.793400	−0.903300

H	−3.232200	−2.528000	−1.566200
C	−1.560300	−0.126400	1.072600
C	−0.738600	−1.159500	0.292300
C	−1.387500	−1.958900	−0.578500
H	−0.862400	−2.745500	−1.107100
C	−2.069100	−0.804200	2.386900
H	−2.802800	−0.143600	2.857700
H	−1.258400	−0.968200	3.097400
H	−2.547800	−1.763300	2.170000
C	−0.752100	1.142400	1.443000
H	−1.189100	1.589200	2.343300
H	−0.881400	1.881100	0.646000
C	0.746400	0.932000	1.670500
H	0.934700	0.440400	2.631300
H	1.199200	1.925600	1.749600
C	0.765700	−1.304000	0.551200
C	1.405600	0.132700	0.529100
C	1.457800	−2.161400	−0.525900
H	1.008300	−3.160100	−0.554600
H	1.296200	−1.718300	−1.516300
C	2.948800	0.044700	0.767500
H	3.041300	−0.283300	1.812800
C	3.736500	−1.023400	−0.065800
C	2.949200	−2.335000	−0.244800
H	3.078100	−2.958600	0.646500
H	3.408700	−2.902200	−1.064100
C	0.964300	−2.037800	1.900700
H	0.482600	−1.543700	2.739900
H	2.019000	−2.144700	2.161100
H	0.535800	−3.042900	1.821500
C	1.056900	0.849700	−0.792300
H	−0.001900	0.740800	−1.045300
H	1.620400	0.477200	−1.647800
H	1.257500	1.922900	−0.701300
C	4.147800	−0.509800	−1.468700
H	3.273400	−0.387200	−2.118100
H	4.808400	−1.241800	−1.944800
C	3.686000	1.405700	0.720500
H	3.074400	2.190900	1.176500
H	4.577000	1.331000	1.357000
C	4.164900	1.912600	−0.657700
C	4.896800	0.802300	−1.386800
C	5.042000	−1.361200	0.675900
H	4.835000	−1.688100	1.701300
H	5.730100	−0.510600	0.720200
H	5.565800	−2.174100	0.160800
C	5.030500	3.158000	−0.521800
H	4.479800	3.958800	−0.019400
H	5.358800	3.515000	−1.500300
H	5.928400	2.933600	0.062100
O	−6.729800	0.986500	−1.075400
H	−6.887500	1.879200	−0.704300

C	−5.561000	−1.519600	−1.847900
H	−6.585000	−1.205400	−2.053600
H	−5.591600	−2.497700	−1.355400
H	−5.045000	−1.646800	−2.806500
H	3.286000	2.144600	−1.273500
O	6.011500	0.940600	−1.841000

M06-2X/6-31G(d) Free Energy = −1313.221173

oq-6-oxotingenol

C	−3.215100	1.736700	0.544900
C	−5.357400	1.067600	−0.699300
C	−3.551900	−0.548100	−0.312300
C	−4.783000	−0.309300	−0.835900
C	−2.759100	0.474400	0.419000
C	−4.507300	2.150200	−0.006600
H	−2.683600	2.508100	1.089200
O	−4.936100	3.279000	0.079700
C	−2.888100	−1.866700	−0.556500
C	−1.494000	0.022300	1.165500
C	−0.729600	−1.058100	0.373900
C	−1.426600	−1.912800	−0.388800
H	−0.964800	−2.750300	−0.898200
C	−2.040500	−0.598900	2.485300
H	−2.728900	0.110800	2.953500
H	−1.239400	−0.804200	3.197300
H	−2.577000	−1.532700	2.291900
C	−0.615400	1.247900	1.519200
H	−1.027000	1.726600	2.415100
H	−0.695900	1.985500	0.716600
C	0.870800	0.964600	1.734000
H	1.047000	0.438100	2.678200
H	1.367300	1.935100	1.834400
C	0.782700	−1.240800	0.574500
C	1.474800	0.167600	0.561900
C	1.420800	−2.106800	−0.528900
H	0.939000	−3.088900	−0.566100
H	1.259300	−1.643500	−1.510100
C	3.018600	0.024200	0.763100
H	3.124400	−0.316700	1.802800
C	3.749500	−1.060900	−0.097700
C	2.909100	−2.338700	−0.269600
H	3.023000	−2.968600	0.619700
H	3.333600	−2.922500	−1.095900
C	0.971100	−2.001800	1.912200
H	0.461100	−1.535300	2.751800
H	2.023400	−2.090600	2.189200
H	0.565300	−3.013200	1.803600
C	1.123400	0.912700	−0.743400
H	0.060700	0.823300	−0.990100
H	1.673600	0.545000	−1.608900
H	1.341200	1.981400	−0.639400
C	4.150100	−0.547500	−1.503700

H	3.267800	−0.387000	−2.133900
H	4.774200	−1.297400	−2.000600
C	3.797600	1.362000	0.711400
H	3.220900	2.162400	1.186300
H	4.698800	1.253400	1.328300
C	4.262800	1.864900	−0.672900
C	4.945900	0.737300	−1.423100
C	5.056600	−1.452900	0.613500
H	4.858700	−1.786600	1.638500
H	5.773100	−0.625800	0.654900
H	5.543500	−2.275500	0.078100
C	5.167100	3.083500	−0.546300
H	4.647900	3.898100	−0.032300
H	5.489500	3.434900	−1.528700
H	6.067400	2.831300	0.022300
O	−6.451000	1.359600	−1.122000
C	−5.639900	−1.293600	−1.575100
H	−6.526300	−0.784600	−1.953200
H	−5.938700	−2.114800	−0.919200
H	−5.089000	−1.754900	−2.398800
H	3.379000	2.127600	−1.268900
O	6.057600	0.842100	−1.892500
O	−3.498700	−2.848600	−0.942300

M06-2X/6-31G(d) Free Energy = −1387.21519

oq-isopristimerol

H	−2.997000	2.379000	1.019200
C	−3.620600	1.620500	0.558400
C	−5.391500	−0.379300	−0.611800
C	−3.221800	0.344400	0.404900
C	−4.959600	2.062300	0.150600
C	−5.911000	0.996900	−0.438100
C	−4.117700	−0.666100	−0.233900
O	−5.347000	3.202700	0.276100
O	−7.033900	1.324300	−0.755600
C	−6.320700	−1.373100	−1.253900
H	−6.511900	−2.226800	−0.594900
H	−5.905100	−1.761700	−2.189200
H	−7.272900	−0.888200	−1.471700
C	−1.890400	−0.164400	0.954900
C	−3.552200	−1.993400	−0.481500
H	−4.233400	−2.797600	−0.740100
C	−2.230900	−2.206900	−0.451200
H	−1.848800	−3.199200	−0.676700
C	−1.268700	−1.090100	−0.140300
H	−1.243900	−0.470600	−1.048600
C	−0.912100	0.980900	1.260100
H	−1.269300	1.543900	2.131100
H	−0.903300	1.685200	0.419600
C	0.195300	−1.556100	0.101600
C	0.520300	0.505400	1.531800
H	0.561100	−0.072800	2.463500

H	1.130000	1.398500	1.705300
C	1.117200	−0.307700	0.361700
C	−2.248000	−0.879500	2.281500
H	−2.895700	−0.222700	2.870400
H	−1.361200	−1.085500	2.881700
H	−2.778100	−1.820600	2.116900
C	0.727300	−2.243000	−1.170900
H	0.672700	−1.538900	−2.008700
H	0.091200	−3.090900	−1.452100
C	2.541600	−0.834200	0.733400
H	2.366500	−1.402400	1.656700
C	2.154000	−2.774400	−0.992000
H	2.094700	−3.725000	−0.451100
H	2.558400	−3.036900	−1.977600
C	3.185300	−1.855800	−0.271800
C	0.232400	−2.572600	1.265300
H	0.201600	−2.099600	2.246800
H	1.131200	−3.190700	1.247800
H	−0.624400	−3.252700	1.208200
C	1.157900	0.637600	−0.857300
H	0.175100	0.798600	−1.305500
H	1.815600	0.296000	−1.654000
H	1.510800	1.618700	−0.531100
C	3.579700	0.233900	1.178800
H	3.089200	1.045300	1.723600
H	4.231000	−0.253100	1.915700
C	4.051900	−1.158300	−1.346800
H	3.432000	−0.599100	−2.054000
H	4.543100	−1.944000	−1.935700
C	4.548300	0.849300	0.142400
C	5.121400	−0.230700	−0.778600
H	5.669900	0.246600	−1.596600
H	5.851000	−0.813700	−0.203600
C	4.105000	−2.792400	0.537100
H	3.526700	−3.350100	1.282800
H	4.897900	−2.258700	1.068700
H	4.582600	−3.520800	−0.128900
C	5.696600	1.537300	0.908100
H	6.252200	0.787600	1.480400
H	5.314500	2.293100	1.599200
H	6.393500	2.020000	0.214200
C	3.915100	1.947000	−0.703300
O	3.934700	2.012000	−1.908200
O	3.371400	2.916600	0.063100
C	2.836500	4.023200	−0.661900
H	3.618200	4.500100	−1.257200
H	2.448200	4.711200	0.087500
H	2.038300	3.691100	−1.331100

M06-2X/6-31G(d) Free Energy = −1466.935292

Cartesian Coordinates of All the Stationary Points for Transition States Included in this Study at M06_2x/6-31G* Level of Theory.

Endo Chanel
Cangorosin A

H	4.409600	−0.174400	3.183300
C	4.937700	−0.680600	2.380100
C	6.417800	−1.987400	0.376900
C	4.889600	−0.197000	1.070400
C	5.680000	−1.810600	2.674000
C	6.448800	−2.443400	1.687600
C	5.580400	−0.890300	0.062800
O	5.767400	−2.374500	3.919900
H	5.231900	−1.867800	4.545700
O	7.208300	−3.522300	2.025800
H	7.088700	−3.676900	2.976200
C	4.325700	1.186100	0.748000
C	3.559400	1.100500	−0.612400
H	2.804000	0.310500	−0.442900
C	5.404100	−0.468000	−1.329900
H	6.208200	−0.660500	−2.030900
C	4.463400	0.527500	−1.682100
H	4.691700	1.125300	−2.556300
O	4.615400	−2.254100	−1.955100
O	3.178800	−0.392100	−2.928600
C	2.551200	−1.308000	−2.315100
C	1.417600	−3.297700	−0.668500
C	1.131700	−1.364700	−2.142400
C	3.380400	−2.328400	−1.699200
C	2.794300	−3.254400	−0.763000
C	0.574400	−2.383500	−1.420500
H	0.528700	−0.657600	−2.701700
C	3.710600	−4.169000	0.003800
H	3.433100	−5.221700	−0.113300
H	3.691200	−3.934200	1.074800
H	4.736000	−4.042100	−0.346700
C	0.752600	−4.260700	0.214300
H	1.352900	−5.051600	0.653400
C	−0.547200	−4.158800	0.517600
H	−0.992500	−4.878900	1.198800
C	−1.388100	−3.034700	−0.035600
H	−1.108200	−2.149600	0.558200
C	−0.921400	−2.697700	−1.483300
C	−2.920400	−3.226900	0.157000
C	−1.747900	−1.516700	−2.011600
H	−1.516800	−1.353200	−3.071300
H	−1.451000	−0.599000	−1.486200
C	−3.259000	−1.724400	−1.863900
H	−3.594700	−2.546200	−2.509200
H	−3.754300	−0.822900	−2.240800
C	−3.695600	−1.974800	−0.405200
C	−1.030000	−3.867300	−2.493000
H	−0.446800	−3.611000	−3.382900
H	−2.056500	−4.045600	−2.816100
H	−0.630300	−4.798200	−2.083100

C	−3.247400	−3.307400	1.660500
H	−2.890200	−2.398300	2.157000
H	−2.703700	−4.135200	2.130600
C	−5.223000	−2.299800	−0.344300
H	−5.315300	−3.181400	−0.991300
C	−4.743800	−3.531500	1.925100
H	−4.951900	−3.316600	2.980800
H	−4.944900	−4.602300	1.812400
C	−5.774200	−2.747000	1.055500
C	−3.346100	−0.699100	0.386800
H	−3.634700	0.179100	−0.195600
H	−2.274800	−0.599200	0.578400
H	−3.848300	−0.642400	1.350700
C	−3.364900	−4.549300	−0.510400
H	−3.527500	−4.453000	−1.584100
H	−4.295200	−4.933900	−0.088400
H	−2.603100	−5.322800	−0.367700
C	−6.312500	−1.554700	1.882500
H	−5.497700	−0.910400	2.226700
H	−6.772900	−1.964100	2.791300
C	−6.186200	−1.283800	−1.020500
H	−5.722300	−0.845000	−1.908100
H	−7.036200	−1.866200	−1.398800
C	−6.827200	−0.154400	−0.183000
C	−7.343400	−0.700100	1.151400
H	−7.651600	0.134800	1.788700
H	−8.243100	−1.293000	0.947400
C	−6.952700	−3.710800	0.808900
H	−7.760600	−3.258200	0.226500
H	−7.377400	−4.043300	1.763600
H	−6.614200	−4.600400	0.265700
C	−7.998100	0.440000	−0.990300
H	−7.659000	0.812400	−1.960500
H	−8.469700	1.265300	−0.445600
H	−8.755000	−0.333100	−1.156400
C	−5.883700	1.011000	0.078600
O	−5.660400	1.503500	1.160400
O	−5.362000	1.503300	−1.060000
C	−4.521300	2.648200	−0.898500
H	−5.065000	3.448500	−0.391000
H	−4.236100	2.951000	−1.905600
H	−3.634500	2.399900	−0.308100
C	7.277400	−2.665900	−0.657800
H	7.989100	−1.952800	−1.090600
H	6.666000	−3.060400	−1.472000
H	7.847900	−3.478000	−0.206500
C	3.369800	1.688400	1.837600
H	3.932400	1.853800	2.765100
H	2.626800	0.910900	2.057600
C	2.798800	2.411000	−1.017500
C	2.655000	2.987900	1.468700
H	3.377100	3.813200	1.405700

H	1.973400	3.239900	2.288300
C	1.849500	2.877100	0.161100
C	5.575100	2.103900	0.727100
H	6.207300	1.896000	−0.141700
H	6.168000	1.903500	1.625200
H	5.325700	3.165100	0.727500
C	1.299200	4.291100	−0.230400
H	2.212200	4.869200	−0.410700
C	1.877300	2.157700	−2.228200
H	2.462000	1.823000	−3.089100
H	1.212000	1.323500	−1.990100
C	0.498300	4.351900	−1.568400
C	1.072700	3.397900	−2.657600
H	1.714800	3.994800	−3.315200
H	0.245700	3.069900	−3.301300
C	3.821900	3.505100	−1.414700
H	4.175700	4.093100	−0.566900
H	3.397100	4.212900	−2.129700
H	4.703900	3.067900	−1.890500
C	0.744500	1.822100	0.413700
H	1.136900	0.802000	0.388700
H	−0.060400	1.851600	−0.317700
H	0.313100	1.964500	1.406900
C	0.598200	5.133700	0.865400
H	1.088200	4.999200	1.833000
H	0.757200	6.186500	0.600500
C	−1.010100	4.077600	−1.346400
H	−1.190200	3.029800	−1.090700
H	−1.524100	4.243700	−2.303400
C	−0.918700	4.973800	1.063900
C	−1.651900	4.972100	−0.284000
H	−2.699600	4.691100	−0.132000
H	−1.665600	6.007700	−0.644400
C	0.624500	5.776700	−2.148300
H	1.676900	6.023800	−2.328900
H	0.211900	6.549200	−1.494100
H	0.098200	5.839100	−3.108400
C	−1.430500	6.154000	1.916300
H	−1.243200	7.096200	1.391300
H	−0.919700	6.180400	2.882800
H	−2.508600	6.068200	2.093000
C	−1.196400	3.760300	1.941400
O	−0.574300	3.513100	2.950300
O	−2.255100	3.037700	1.551300
C	−2.637900	1.987200	2.446400
H	−1.893200	1.186400	2.420600
H	−3.606700	1.639100	2.091600
H	−2.708900	2.373800	3.464700

M06-2X/6-31G(d) Free Energy = −2935.023107

Cangorosin Aβ

H	−4.664400	2.948500	−2.306400

C	−4.493400	3.444700	−1.354700
C	−4.062500	4.812400	1.056700
C	−3.897900	2.763100	−0.290000
C	−4.874000	4.767200	−1.217500
C	−4.658500	5.456400	−0.017300
C	−3.686800	3.452600	0.919100
O	−5.471000	5.505700	−2.205500
H	−5.568100	4.966900	−3.002400
O	−5.040800	6.759700	0.083200
H	−5.414100	7.020900	−0.773500
C	−3.372400	1.335800	−0.477400
C	−3.541600	0.586200	0.880800
H	−4.620200	0.698800	1.090400
C	−3.141300	2.754200	2.078400
H	−3.287400	3.218100	3.043500
C	−3.004000	1.352000	2.073400
H	−3.163100	0.874100	3.030200
O	−1.271000	3.601900	2.352500
O	−1.072000	1.021700	2.582200
C	−0.199600	1.605800	1.864600
C	1.485500	3.035200	0.102200
C	0.803400	0.916900	1.110700
C	−0.344600	3.044200	1.699100
C	0.468300	3.730500	0.724200
C	1.653100	1.604300	0.291900
H	0.879200	−0.156500	1.245000
C	0.215200	5.198500	0.511700
H	1.013000	5.814500	0.941900
H	−0.725800	5.477700	0.988900
H	0.147500	5.439900	−0.553500
C	2.447900	3.724600	−0.763600
H	2.210900	4.729400	−1.099800
C	3.615300	3.158300	−1.092500
H	4.322300	3.706400	−1.709600
C	3.973700	1.787100	−0.572500
H	4.204500	1.942200	0.492900
C	2.699300	0.889400	−0.567500
C	5.251800	1.168400	−1.206000
C	3.062100	−0.495000	−0.007700
H	2.213200	−1.177300	−0.141000
H	3.228100	−0.418400	1.073900
C	4.298600	−1.114200	−0.668200
H	4.092900	−1.340700	−1.722000
H	4.480700	−2.078900	−0.182400
C	5.555700	−0.226800	−0.540500
C	2.029000	0.703200	−1.949600
H	1.040000	0.258200	−1.795800
H	2.593800	0.032100	−2.598800
H	1.894300	1.655600	−2.469200
C	6.461600	2.073700	−0.906800
H	6.568900	2.181300	0.178500
H	6.292500	3.086200	−1.292100

C	6.758100	−0.870700	−1.301900
H	6.374400	−0.988700	−2.324100
C	7.756700	1.546800	−1.537000
H	8.607000	2.085300	−1.100200
H	7.756800	1.832400	−2.594500
C	8.045200	0.019100	−1.437800
C	5.848700	−0.087500	0.967600
H	5.720800	−1.062000	1.444300
H	5.162900	0.594200	1.474800
H	6.857500	0.258200	1.183900
C	5.078200	1.065800	−2.739800
H	4.512800	0.186100	−3.048900
H	6.034700	1.018500	−3.263200
H	4.545200	1.942900	−3.123000
C	9.048000	−0.213500	−0.282700
H	8.667300	0.188100	0.661200
H	9.952800	0.364900	−0.511100
C	7.133100	−2.327100	−0.903200
H	6.238800	−2.892100	−0.626800
H	7.508900	−2.812000	−1.813400
C	9.437300	−1.674000	−0.078700
H	10.125700	−1.759100	0.768000
H	9.971200	−2.044400	−0.962300
C	8.228900	−2.582900	0.158600
C	8.747000	−0.375400	−2.752900
H	9.009500	−1.436300	−2.794100
H	9.671300	0.201300	−2.879200
H	8.100400	−0.163300	−3.612000
C	8.663000	−4.058900	0.054700
H	7.812300	−4.731200	0.193400
H	9.420700	−4.295500	0.810000
H	9.099100	−4.242200	−0.932600
C	7.741400	−2.409000	1.591400
O	8.272800	−1.739500	2.443300
O	6.654500	−3.169000	1.844800
C	6.185000	−3.112500	3.190800
H	6.958100	−3.463200	3.878200
H	5.311100	−3.761000	3.228500
H	5.916000	−2.087100	3.457800
C	−3.831200	5.576700	2.334100
H	−3.984900	6.643900	2.170600
H	−2.815200	5.410400	2.701000
H	−4.528900	5.256000	3.117300
C	−4.173900	0.578000	−1.557700
H	−3.914700	0.981300	−2.544600
H	−5.246200	0.761300	−1.419200
C	−3.317900	−0.960300	0.868200
C	−3.914200	−0.931700	−1.571700
H	−2.868500	−1.134900	−1.835700
H	−4.520900	−1.365100	−2.374600
C	−4.252800	−1.603900	−0.226200
C	−1.928400	1.502700	−1.002000

H	−1.235200	1.802800	−0.217200
H	−1.921100	2.293200	−1.760300
H	−1.543800	0.597500	−1.477300
C	−3.974800	−3.141300	−0.310800
H	−2.956000	−3.197600	−0.714000
C	−3.719900	−1.577700	2.220200
H	−3.143800	−1.121100	3.032000
H	−4.777900	−1.373100	2.429300
C	−3.930400	−3.924600	1.051800
C	−3.426300	−3.080000	2.252800
H	−2.342500	−3.205000	2.348800
H	−3.846700	−3.507500	3.171800
C	−1.826200	−1.288400	0.650100
H	−1.268600	−0.928600	1.517000
H	−1.398800	−0.817200	−0.233000
H	−1.648200	−2.360800	0.558800
C	−5.750000	−1.357800	0.047800
H	−5.985000	−0.329200	0.328400
H	−6.155300	−2.001300	0.827200
H	−6.305800	−1.558300	−0.873000
C	−4.809000	−3.919300	−1.374500
H	−5.041200	−3.274700	−2.227800
H	−4.148000	−4.697600	−1.776100
C	−5.304700	−4.517900	1.435600
H	−6.013700	−3.728100	1.704900
H	−5.162300	−5.115100	2.345800
C	−6.102600	−4.675500	−0.972400
C	−5.923000	−5.403400	0.361300
H	−6.893100	−5.779300	0.701200
H	−5.285500	−6.278800	0.188000
C	−2.938600	−5.091900	0.882600
H	−1.926500	−4.711700	0.702400
H	−3.196600	−5.748200	0.046000
H	−2.908800	−5.704200	1.792000
C	−6.420100	−5.698000	−2.081900
H	−5.610200	−6.431900	−2.143500
H	−6.524800	−5.208500	−3.053500
H	−7.349100	−6.235700	−1.862100
C	−7.330500	−3.776000	−0.885100
O	−8.064500	−3.668800	0.067000
O	−7.566800	−3.135800	−2.050100
C	−8.725000	−2.303600	−2.048200
H	−8.788500	−1.878100	−3.048700
H	−9.616300	−2.891500	−1.819400
H	−8.622800	−1.514200	−1.298500

M06-2X/6-31G(d) Free Energy = −2935.016706

isocangorosin A

H	1.059100	2.337800	−3.117700
C	0.926600	3.052100	−2.309600
C	0.545600	4.960100	−0.286800
C	1.863600	3.149800	−1.275300

C	−0.189600	3.868100	−2.317800
C	−0.366400	4.840000	−1.327900
C	1.654400	4.082700	−0.249000
O	−1.179000	3.822100	−3.265800
H	−0.962300	3.153200	−3.929500
O	−1.457700	5.653800	−1.366800
H	−1.956600	5.435100	−2.170000
C	3.201700	2.419600	−1.359300
C	3.580600	1.934700	0.085600
H	2.702900	1.329600	0.377000
C	2.536400	4.058900	0.939000
H	2.728000	4.997700	1.445100
C	3.545600	3.064100	1.075400
H	4.433000	3.323200	1.640900
O	2.961300	2.055600	2.839600
O	1.139500	3.651900	2.151700
C	0.841000	2.412000	2.068900
C	0.486400	−0.363200	1.832700
C	−0.249100	1.903400	1.302000
C	1.849900	1.504900	2.563900
C	1.589400	0.085500	2.536200
C	−0.419900	0.552900	1.166400
H	−0.905500	2.626700	0.826800
C	2.459600	−0.835600	3.353000
H	2.911700	−1.638500	2.759000
H	1.864600	−1.317500	4.137000
H	3.259300	−0.265900	3.830100
C	0.232500	−1.802300	1.689800
H	1.028700	−2.497300	1.944400
C	−0.938600	−2.259400	1.229000
H	−1.089700	−3.330900	1.122900
C	−2.054800	−1.300300	0.894600
H	−2.417200	−0.938100	1.868700
C	−1.458900	−0.034000	0.208000
C	−3.277900	−1.950400	0.191900
C	−2.593700	0.955800	−0.100400
H	−2.204300	1.776900	−0.717000
H	−2.942000	1.412700	0.834200
C	−3.782100	0.313200	−0.825000
H	−3.486000	−0.007200	−1.832100
H	−4.539200	1.092000	−0.967800
C	−4.398400	−0.868000	−0.042000
C	−0.674100	−0.298400	−1.099700
H	−0.127200	0.617400	−1.354100
H	−1.327100	−0.539200	−1.940900
H	0.055000	−1.106700	−0.985100
C	−3.885000	−3.027700	1.110700
H	−4.186400	−2.563400	2.056600
H	−3.133800	−3.781900	1.373000
C	−5.548000	−1.533200	−0.865400
H	−5.060100	−1.795300	−1.814100
C	−5.065400	−3.752600	0.454900

H	−5.591400	−4.337100	1.220000
H	−4.662300	−4.498000	−0.239100
C	−6.114500	−2.885800	−0.300600
C	−4.921200	−0.289200	1.289400
H	−5.427400	0.657600	1.086700
H	−4.123600	−0.063200	2.000000
H	−5.626900	−0.941000	1.801400
C	−2.823000	−2.622200	−1.125600
H	−1.844700	−3.097600	−0.991100
H	−2.727500	−1.917600	−1.951900
H	−3.513500	−3.398300	−1.460500
C	−7.326000	−2.672600	0.636900
H	−7.017800	−2.215900	1.582300
H	−7.718200	−3.664100	0.898900
C	−6.704600	−0.595400	−1.316200
H	−6.325400	0.409700	−1.521400
H	−7.054300	−0.973900	−2.285200
C	−8.452500	−1.846700	0.026800
H	−9.255400	−1.717700	0.759400
H	−8.885000	−2.379200	−0.829100
C	−7.986100	−0.472000	−0.457500
C	−6.591800	−3.727800	−1.500900
H	−7.348900	−3.218700	−2.104400
H	−7.023500	−4.674200	−1.153900
H	−5.749700	−3.966500	−2.160900
C	−9.084600	0.172900	−1.326400
H	−8.762500	1.144200	−1.710900
H	−10.005100	0.314800	−0.749500
H	−9.312700	−0.482100	−2.173400
C	−7.829300	0.455200	0.742000
O	−8.107000	0.185300	1.885200
O	−7.403600	1.682100	0.373900
C	−7.306800	2.625500	1.440200
H	−8.281500	2.765800	1.912400
H	−6.959100	3.552800	0.987300
H	−6.597200	2.275300	2.194500
C	0.254300	6.001800	0.760400
H	1.135300	6.258300	1.348600
H	−0.504300	5.635400	1.458100
H	−0.132500	6.907500	0.286800
C	3.125700	1.199500	−2.298600
H	3.046400	1.553400	−3.334600
H	2.207100	0.636300	−2.091500
C	4.827900	0.994200	0.152000
C	4.324800	0.253300	−2.196500
H	5.232700	0.746600	−2.564700
H	4.144200	−0.586700	−2.876400
C	4.549000	−0.261000	−0.759400
C	4.160900	3.466900	−1.989000
H	4.553400	4.162300	−1.240200
H	3.606700	4.052000	−2.729000
H	5.000400	3.007900	−2.514200

C	5.773600	−1.238000	−0.737800
H	6.543900	−0.702800	−1.310700
C	5.070200	0.477300	1.577500
H	5.153100	1.306200	2.285400
H	4.200000	−0.095300	1.896400
C	6.434700	−1.541800	0.656400
C	6.332100	−0.377000	1.671400
H	7.210900	0.270900	1.584500
H	6.393400	−0.801400	2.681900
C	6.103100	1.758300	−0.264600
H	6.266900	2.599500	0.420300
H	6.077600	2.159400	−1.271900
H	6.990700	1.126700	−0.206200
C	3.272300	−1.017500	−0.344000
H	2.398500	−0.379100	−0.207000
H	3.391600	−1.585900	0.579400
H	3.012400	−1.718100	−1.142500
C	5.563300	−2.555500	−1.550600
H	4.887800	−2.381300	−2.394600
H	6.532000	−2.800200	−2.004500
C	5.828100	−2.789300	1.333900
H	4.793400	−2.596000	1.638200
H	6.387800	−2.969700	2.260500
C	5.128000	−3.871800	−0.845100
C	5.874600	−4.047400	0.477900
H	5.446400	−4.891500	1.027500
H	6.916100	−4.307000	0.252800
C	7.933200	−1.800100	0.411600
H	8.419400	−0.892900	0.034400
H	8.114400	−2.595300	−0.318300
H	8.432100	−2.083100	1.346300
C	5.456400	−5.044000	−1.791100
H	6.538700	−5.094300	−1.947200
H	4.966900	−4.919600	−2.760300
H	5.131500	−5.997100	−1.359200
C	3.629300	−3.975300	−0.570100
O	3.119800	−4.147800	0.512300
O	2.900100	−3.923100	−1.702900
C	1.489300	−4.015200	−1.504900
H	1.039900	−3.875100	−2.487400
H	1.227400	−4.994600	−1.097000
H	1.150100	−3.242600	−0.807300

M06-2X/6-31G(d) Free Energy = −2935.021773

isocangorosin Aβ

H	−0.057100	−2.308900	0.630900
C	−0.156100	−1.701000	1.523800
C	−0.460000	−0.219700	3.892400
C	0.580700	−0.525800	1.687300
C	−1.007000	−2.147300	2.522400
C	−1.142500	−1.417200	3.710400
C	0.416400	0.224200	2.872700

O	−1.682200	−3.344700	2.473500
H	−2.325500	−3.362600	1.735200
O	−1.968100	−1.903400	4.683000
H	−2.203900	−2.802200	4.393200
C	1.513900	−0.037600	0.570900
C	2.707900	0.710000	1.239600
H	3.094900	−0.041600	1.950100
C	1.218700	1.427600	3.113200
C	2.274100	1.768600	2.237400
O	1.779800	3.604700	1.428500
O	−0.038900	3.004300	3.163800
C	−0.439700	3.331300	2.005900
C	−1.129300	3.820100	−0.684700
C	−1.780300	3.110900	1.558600
C	0.570600	3.730400	1.047500
C	0.176700	4.047300	−0.303900
C	−2.123400	3.324100	0.255700
H	−2.483700	2.748100	2.299700
C	1.223300	4.539700	−1.268200
H	0.875400	5.425000	−1.810400
H	1.478200	3.776800	−2.013600
H	2.134000	4.806500	−0.730500
C	−1.513500	3.893500	−2.103700
H	−0.963100	4.551000	−2.769900
C	−2.471100	3.078500	−2.564400
H	−2.737400	3.074100	−3.618300
C	−3.153100	2.150500	−1.586900
H	−2.348000	1.477600	−1.259800
C	−3.507600	2.976900	−0.298200
C	−4.244900	1.224500	−2.169300
C	−4.337700	2.144600	0.699100
H	−4.778600	2.820400	1.442900
H	−3.691100	1.464400	1.258300
C	−5.438300	1.309600	0.038900
H	−6.200700	1.948000	−0.424800
H	−5.946000	0.746600	0.830400
C	−4.841700	0.344000	−1.006700
C	−4.283000	4.291700	−0.545600
H	−4.199200	4.916400	0.349800
H	−5.348900	4.106300	−0.707800
H	−3.895500	4.859300	−1.393700
C	−3.609500	0.262100	−3.189200
H	−2.841000	−0.342800	−2.690800
H	−3.090300	0.828200	−3.973100
C	−5.945500	−0.602500	−1.583500
H	−6.740000	0.082900	−1.907400
C	−4.666000	−0.629600	−3.838400
H	−4.185400	−1.342000	−4.521800
H	−5.305500	−0.002000	−4.470200
C	−5.555200	−1.427900	−2.856800
C	−3.741100	−0.465200	−0.281700
H	−4.109400	−0.734400	0.710600

H	−2.821400	0.095400	−0.104800
H	−3.458800	−1.380400	−0.794000
C	−5.307900	2.078800	−2.892800
H	−5.650100	2.920000	−2.290300
H	−6.190800	1.509700	−3.190800
H	−4.880100	2.495700	−3.811800
C	−4.838400	−2.750100	−2.498600
H	−3.830500	−2.561800	−2.118500
H	−4.697300	−3.312900	−3.431000
C	−6.671500	−1.517400	−0.560700
H	−6.828400	−0.992400	0.385100
H	−7.677400	−1.692900	−0.960700
C	−5.612400	−3.637100	−1.523200
H	−5.000800	−4.498800	−1.237500
H	−6.494000	−4.038200	−2.036700
C	−6.108500	−2.929900	−0.253400
C	−6.842700	−1.778700	−3.626400
H	−7.530700	−2.406000	−3.052500
H	−6.594000	−2.319000	−4.547700
H	−7.381200	−0.866700	−3.909000
C	−7.244400	−3.768200	0.374700
H	−7.646600	−3.283600	1.267900
H	−6.887800	−4.767300	0.649300
H	−8.051500	−3.885100	−0.354900
C	−5.019600	−2.898400	0.811300
O	−3.926500	−3.422800	0.725100
O	−5.436700	−2.261100	1.911600
C	−4.507200	−2.167000	2.991600
H	−4.170700	−3.163600	3.284100
H	−5.041900	−1.683400	3.806700
H	−3.645800	−1.560400	2.698200
C	−0.658800	0.557100	5.167900
H	0.230200	0.501600	5.807900
H	−1.497800	0.150900	5.734000
H	−0.845000	1.610700	4.945300
C	2.077100	−1.231100	−0.230900
H	1.279000	−1.658300	−0.849000
H	2.383000	−2.023300	0.463200
C	3.947800	1.037600	0.341600
C	3.252000	−0.875200	−1.143100
H	2.921800	−0.185300	−1.929700
H	3.561500	−1.794300	−1.652600
C	4.443600	−0.281000	−0.365400
C	0.621400	0.811800	−0.353500
H	0.069500	1.555200	0.219900
H	−0.126300	0.152300	−0.813800
H	1.161600	1.318100	−1.155400
C	5.601400	0.092900	−1.348200
H	5.114500	0.757700	−2.073200
C	5.108900	1.532200	1.225400
H	4.817100	2.437500	1.768100
H	5.338000	0.774900	1.985300

C	6.783600	0.928800	−0.737700
C	6.356800	1.890900	0.408700
H	6.202300	2.888900	−0.014000
H	7.205000	2.003500	1.095700
C	3.617400	2.150700	−0.669900
H	4.516900	2.583600	−1.111600
H	3.081100	2.947700	−0.155200
H	3.002400	1.806300	−1.502700
C	4.905900	−1.350800	0.643700
H	4.236400	−1.460600	1.499400
H	5.901400	−1.166300	1.042600
H	4.919800	−2.322400	0.143800
C	6.143300	−1.057400	−2.246300
H	5.341200	−1.754000	−2.506000
H	6.441200	−0.595600	−3.196400
C	7.932800	0.033900	−0.216900
H	7.635100	−0.511000	0.684000
H	8.751000	0.697200	0.092700
C	7.382100	−1.875500	−1.806300
C	8.469200	−0.957400	−1.243600
H	9.259100	−1.566900	−0.793600
H	8.923000	−0.416000	−2.082400
C	7.354200	1.815400	−1.863100
H	6.596300	2.524000	−2.216200
H	7.691400	1.238600	−2.729500
H	8.209100	2.395800	−1.495900
C	7.925100	−2.624900	−3.039600
H	8.248100	−1.900000	−3.793800
H	7.160000	−3.270800	−3.478000
H	8.789200	−3.242200	−2.770300
C	7.064000	−2.960700	−0.784500
O	7.623600	−3.125700	0.272100
O	6.103500	−3.799200	−1.229400
H	1.336000	1.729400	4.145000
H	3.065500	2.357300	2.679700
C	5.797200	−4.878700	−0.349000
H	5.028200	−5.463600	−0.851600
H	6.687300	−5.484000	−0.164400
H	5.427100	−4.497200	0.606500

M06-2X/6-31G(d) Free Energy = −2935.014481

isoxuxuarine Aα

C	−5.314600	0.697200	0.510800
C	−5.965600	−1.743900	0.434700
C	−3.929500	−1.059900	1.524600
C	−4.945800	−2.083400	1.358800
C	−4.179900	0.344200	1.152200
C	−6.313100	−0.305900	0.166200
H	−5.563000	1.719800	0.252700
O	−7.408400	−0.074300	−0.320400
C	−2.648700	−1.394700	1.856900
H	−2.415300	−2.437300	2.058400

C	−3.220100	1.392100	1.718000
C	−1.769800	0.888500	1.756700
C	−1.564400	−0.443600	1.853200
H	−0.563500	−0.852800	1.930600
C	−3.721100	1.587600	3.186100
H	−4.809000	1.702400	3.171600
H	−3.299000	2.489400	3.631600
H	−3.464800	0.728300	3.811900
C	−3.378100	2.734200	0.968800
H	−4.292600	3.225100	1.320500
H	−3.530100	2.528300	−0.095700
C	−2.200600	3.697500	1.115800
H	−2.153600	4.113900	2.128900
H	−2.395400	4.544700	0.451000
C	−0.611400	1.892600	1.808900
C	−0.862700	3.026800	0.751300
C	0.740100	1.230700	1.489200
H	0.930000	0.407900	2.187100
H	0.710000	0.787700	0.483600
C	0.293600	4.077700	0.799500
H	0.171000	4.574700	1.772200
C	1.758400	3.516100	0.805500
C	1.897500	2.218400	1.625200
H	2.033400	2.468200	2.683200
H	2.833600	1.725900	1.325600
C	−0.524500	2.422500	3.263900
H	−1.449400	2.869100	3.620000
H	0.256000	3.176600	3.379500
H	−0.284600	1.587700	3.931500
C	−1.022100	2.405100	−0.647600
H	−1.623900	1.490200	−0.616800
H	−0.074400	2.132300	−1.108000
H	−1.523700	3.100600	−1.330100
C	2.305900	3.242200	−0.618500
H	1.796200	2.386200	−1.075900
H	3.371600	2.992600	−0.556600
C	0.164500	5.218800	−0.240900
H	−0.882500	5.517400	−0.353300
H	0.673400	6.103900	0.162500
C	0.746100	4.974000	−1.650900
C	2.160800	4.446000	−1.522400
C	2.685200	4.568400	1.438400
H	2.340600	4.836500	2.443700
H	2.745600	5.484500	0.841800
H	3.702400	4.169900	1.525300
C	0.695400	6.238000	−2.498600
H	−0.334600	6.591500	−2.604200
H	1.109300	6.057300	−3.492900
H	1.290700	7.030800	−2.035000
O	−6.965000	−2.587300	0.238200
H	−7.636600	−2.082600	−0.269500
C	−5.168700	−3.183500	2.355500

H	−5.678100	−4.019600	1.876700
H	−5.791400	−2.811500	3.178100
H	0.169600	4.179300	−2.143300
O	3.110300	4.964100	−2.068300
C	−3.821800	−1.790700	−1.357100
C	−3.225000	−2.900200	−0.632300
C	−1.805400	−3.081100	−0.689500
H	−1.415900	−3.966300	−0.199100
C	−1.020800	−2.233900	−1.425600
C	−1.611800	−1.065300	−2.033000
C	−2.978300	−0.805200	−1.974600
C	−3.655200	0.388700	−2.589700
H	−3.557500	0.375800	−3.678600
H	−3.189900	1.322000	−2.260800
H	−4.713000	0.380600	−2.322200
C	0.430900	−2.615800	−1.751500
C	−0.688400	−0.089700	−2.665100
O	−1.061800	0.857200	−3.343400
C	0.750700	−0.242100	−2.363500
H	1.328600	0.643100	−2.608900
C	1.318000	−1.369800	−1.908800
C	0.982600	−3.626800	−0.718100
H	0.586900	−4.620500	−0.957300
H	0.594400	−3.379600	0.274600
C	2.837300	−1.463700	−1.699500
C	2.507600	−3.699900	−0.634400
H	2.932200	−4.162400	−1.533000
H	2.756100	−4.369400	0.195400
C	3.132100	−2.308800	−0.409700
C	0.331300	−3.348500	−3.124100
H	−0.474800	−4.085900	−3.069900
H	1.252200	−3.882500	−3.367800
H	0.107800	−2.645200	−3.931400
C	3.489500	−0.080900	−1.519800
H	3.064800	0.417100	−0.638100
H	3.266700	0.565000	−2.375200
C	4.673300	−2.440200	−0.182300
H	5.014100	−3.077800	−1.009500
C	5.009100	−0.187300	−1.410800
H	5.413000	−0.511600	−2.376200
H	5.431400	0.811500	−1.241000
C	5.520700	−1.125700	−0.301600
C	3.433200	−2.086300	−2.988500
H	3.316200	−1.371000	−3.809800
H	2.939800	−3.006800	−3.290400
H	4.497600	−2.308800	−2.888800
C	2.418900	−1.679700	0.803400
H	2.370400	−2.400100	1.628400
H	1.391000	−1.396900	0.555400
H	2.909400	−0.779100	1.174200
C	5.577100	−0.319900	1.020800
H	4.603400	0.128700	1.251900

H	6.296700	0.498600	0.916000
C	5.070200	−3.218800	1.096300
H	4.385300	−4.056200	1.264600
H	6.050200	−3.680700	0.921200
C	6.016800	−1.176000	2.187400
C	5.182400	−2.422500	2.414200
H	4.183600	−2.065300	2.699200
C	6.971500	−1.507400	−0.644100
H	7.458800	−2.067600	0.160900
H	7.566200	−0.603400	−0.816300
H	7.008700	−2.115000	−1.555400
O	6.981900	−0.900600	2.865900
C	5.750500	−3.281000	3.536400
H	5.130400	−4.168400	3.695900
H	5.806800	−2.717200	4.470100
H	6.765800	−3.605600	3.289200
O	−3.992900	−3.567700	0.105800
O	−5.094300	−1.695300	−1.303400
H	−4.224200	−3.537800	2.772300

M06-2X/6-31G(d) Free Energy = −2700.395799

isoxuxuarine Aβ

C	3.011300	3.459400	0.860000
C	1.949800	5.012600	−0.811000
C	2.221900	2.643900	−1.311500
C	1.875100	3.982900	−1.776200
C	2.778400	2.415100	0.027900
C	2.700200	4.824200	0.468600
H	3.446800	3.339400	1.845600
O	3.011700	5.827900	1.096000
C	2.017000	1.560200	−2.110000
H	1.542900	1.703400	−3.076700
C	2.975100	0.976100	0.533700
C	3.026400	−0.067500	−0.584700
C	2.476900	0.238000	−1.779800
H	2.419000	−0.499000	−2.571300
C	1.680300	0.713300	1.358400
H	1.567100	1.509900	2.100000
H	1.706400	−0.241200	1.883700
H	0.810300	0.731300	0.697400
C	4.212300	0.910900	1.466200
H	3.936800	1.318400	2.444800
H	4.983000	1.577700	1.065800
C	4.819200	−0.479800	1.667900
H	4.197300	−1.085800	2.335200
H	5.768200	−0.336800	2.194000
C	3.650000	−1.445200	−0.327100
C	5.058200	−1.222800	0.338200
C	3.852700	−2.233800	−1.637200
H	2.888200	−2.385800	−2.135000
H	4.471400	−1.649500	−2.329500
C	5.738200	−2.602700	0.614700

H	5.101800	−3.062500	1.384300
C	5.760900	−3.633500	−0.565700
C	4.464100	−3.614600	−1.398600
H	3.720100	−4.259700	−0.918800
H	4.669900	−4.086800	−2.367500
C	2.687900	−2.282600	0.552900
H	3.048700	−3.303400	0.687800
H	1.709900	−2.336500	0.061100
H	2.534000	−1.875000	1.548600
C	5.925400	−0.300600	−0.544200
H	5.360700	0.567200	−0.898900
H	6.322400	−0.797200	−1.429000
H	6.773600	0.081300	0.034100
C	6.962200	−3.430400	−1.523900
H	6.852300	−2.510900	−2.110000
H	7.007300	−4.264900	−2.231100
C	7.139000	−2.510700	1.267900
H	7.170000	−1.701100	2.003800
H	7.304700	−3.428700	1.845900
C	8.352200	−2.350000	0.327200
C	8.277900	−3.388100	−0.776000
C	5.909400	−5.047800	0.023900
H	5.118100	−5.249900	0.754700
H	6.875900	−5.197000	0.515900
H	5.833200	−5.795900	−0.773100
C	9.666200	−2.437100	1.091900
H	9.712100	−1.669500	1.870400
H	10.518200	−2.309500	0.420400
H	9.766900	−3.418900	1.564300
H	8.286000	−1.375200	−0.174300
O	9.186000	−4.151300	−1.020800
O	0.264700	4.870800	0.292400
O	−0.214900	3.778600	−2.012900
C	−0.378800	3.779900	0.298100
C	−0.663900	3.181700	−0.999900
C	−1.288900	1.893400	−1.041900
H	−1.426600	1.457400	−2.025400
C	−1.765100	1.307100	0.099800
C	−1.490400	1.920600	1.381300
C	−0.748600	3.084100	1.506700
C	−0.346900	3.723900	2.805800
H	0.248600	3.035800	3.411000
H	−1.220100	3.973300	3.415700
H	0.225500	4.629200	2.598000
C	−2.426100	−0.080200	0.022900
C	−2.066100	1.271200	2.588200
O	−1.724000	1.546400	3.727900
C	−3.159500	0.306600	2.361100
H	−3.750700	0.121800	3.250600
C	−3.395500	−0.314000	1.196900
C	−3.106800	−0.265900	−1.357000
H	−2.338100	−0.520500	−2.095900

H	−3.528400	0.691200	−1.677400
C	−4.542700	−1.329800	1.067300
C	−4.210700	−1.322000	−1.408400
H	−3.795400	−2.334000	−1.337800
H	−4.672500	−1.254200	−2.398600
C	−5.266100	−1.105200	−0.307700
C	−1.247300	−1.094000	0.117600
H	−0.458700	−0.785600	−0.578200
H	−1.555100	−2.105000	−0.158300
H	−0.828800	−1.120000	1.129000
C	−5.587600	−1.175900	2.189200
H	−6.011100	−0.163900	2.170500
H	−5.111500	−1.294000	3.167600
C	−6.429300	−2.134200	−0.482600
H	−5.918600	−3.102400	−0.581100
C	−6.686100	−2.234400	2.087700
H	−6.257600	−3.215100	2.323300
H	−7.437100	−2.052200	2.866800
C	−7.407900	−2.310900	0.728500
C	−3.927900	−2.744800	1.214300
H	−3.108800	−2.935200	0.525400
H	−4.664600	−3.535500	1.059400
H	−3.531100	−2.851700	2.229800
C	−5.753100	0.355400	−0.414600
H	−5.956800	0.609400	−1.460800
H	−5.000800	1.061700	−0.049700
H	−6.661900	0.546800	0.154600
C	−8.563000	−1.277200	0.731300
H	−8.194700	−0.271800	0.965800
H	−9.288900	−1.546900	1.505300
C	−7.231300	−1.984200	−1.798500
H	−6.567000	−1.725900	−2.629100
H	−7.645200	−2.967400	−2.057300
C	−9.291800	−1.243800	−0.594900
C	−8.410000	−0.987900	−1.802400
H	−8.013200	0.028000	−1.676800
C	−8.065400	−3.698600	0.628900
H	−8.712600	−3.792700	−0.249400
H	−8.688100	−3.883300	1.511400
H	−7.305500	−4.487000	0.580500
O	−10.483100	−1.444400	−0.685000
C	−9.201900	−1.066500	−3.100700
H	−8.556600	−0.858900	−3.959800
H	−10.027700	−0.351700	−3.099300
H	−9.634000	−2.064500	−3.222000
O	1.836000	6.273900	−1.194500
H	2.086400	6.804700	−0.407100
C	1.958200	4.384000	−3.223100
H	1.343200	5.269500	−3.385100
H	1.606000	3.594900	−3.887300
H	2.995700	4.625800	−3.481300

M06-2X/6-31G(d) Free Energy = −2700.388972

xuxuarine Aα

C	−3.640100	−2.904700	−1.710600
C	−3.448900	−4.685200	0.033700
C	−4.405800	−2.498900	0.581500
C	−3.987600	−3.828900	1.027900
C	−4.117000	−2.040900	−0.776500
C	−3.419600	−4.307200	−1.407200
H	−3.476400	−2.612700	−2.742600
O	−3.227400	−5.195700	−2.228100
C	−4.995300	−1.626600	1.444100
H	−5.233900	−1.955700	2.450600
C	−4.587400	−0.644300	−1.220200
C	−4.872300	0.301500	−0.052300
C	−5.186100	−0.232800	1.147000
H	−5.471600	0.398400	1.979500
C	−5.962500	−0.937800	−1.899400
H	−5.809200	−1.661200	−2.705700
H	−6.414800	−0.038900	−2.319000
H	−6.658400	−1.367800	−1.173300
C	−3.574300	−0.079900	−2.264800
H	−3.871300	−0.416800	−3.264300
H	−2.606600	−0.550600	−2.062200
C	−3.355400	1.438500	−2.287900
H	−4.112900	1.947500	−2.891700
H	−2.401800	1.611700	−2.798800
C	−4.740000	1.811800	−0.246500
C	−3.313900	2.038100	−0.870900
C	−4.825500	2.564000	1.095700
H	−5.808400	2.395300	1.551800
H	−4.081900	2.162300	1.795700
C	−2.952700	3.556800	−0.955600
H	−3.600500	3.951000	−1.753300
C	−3.270500	4.440100	0.294300
C	−4.620100	4.065400	0.926200
H	−5.434100	4.486100	0.323800
H	−4.693300	4.555300	1.905400
C	−5.878300	2.342700	−1.146700
H	−5.726100	3.392500	−1.409600
H	−6.831300	2.263300	−0.612300
H	−5.978500	1.796200	−2.081800
C	−2.266300	1.246100	−0.075700
H	−2.548000	0.199300	0.065700
H	−2.068100	1.648900	0.917300
H	−1.319600	1.228300	−0.623200
C	−2.170900	4.363700	1.383900
H	−2.106300	3.367600	1.837500
H	−2.395900	5.079400	2.181500
C	−1.502400	3.821100	−1.436600
H	−1.212800	3.093200	−2.203300
H	−1.483200	4.794800	−1.942700
C	−0.391200	3.851600	−0.362700
C	−0.816300	4.712400	0.811400

C	−3.357800	5.911100	−0.148400
H	−4.103700	6.034900	−0.941700
H	−2.401400	6.297900	−0.514700
H	−3.655900	6.538900	0.698700
C	0.933900	4.320100	−0.946100
H	1.244300	3.673700	−1.774600
H	1.713900	4.312600	−0.179700
H	0.849900	5.346100	−1.318200
O	−3.344500	−5.978500	0.267800
H	−3.060200	−6.368600	−0.589100
C	−4.612100	−4.493100	2.225300
H	−4.021100	−5.369000	2.493000
H	−5.633400	−4.814500	1.994000
H	−4.630700	−3.824100	3.085700
H	−0.263200	2.846300	0.061100
O	−0.138300	5.623300	1.233200
O	−2.354300	−3.349800	2.115500
O	−1.369100	−4.335000	−0.076400
C	−1.508400	−2.635600	1.498700
C	−0.940300	−3.208300	0.286200
C	0.016800	−2.446600	−0.455800
H	0.402600	−2.910200	−1.357000
C	0.501000	−1.262800	0.033500
C	−0.120900	−0.669300	1.196500
C	−1.164100	−1.283500	1.875700
C	−1.888900	−0.691000	3.051500
H	−2.490600	0.167700	2.734400
H	−1.197400	−0.314400	3.808800
H	−2.542700	−1.448600	3.486700
C	1.624400	−0.541000	−0.735100
C	0.407000	0.639900	1.652600
O	−0.211900	1.399000	2.389400
C	1.771700	0.989000	1.218500
H	2.230600	1.754900	1.833500
C	2.397900	0.438700	0.166800
C	2.554600	−1.598100	−1.390600
H	2.074600	−1.966000	−2.304300
H	2.637600	−2.461300	−0.724600
C	3.834500	0.847600	−0.194300
C	3.960400	−1.109100	−1.736100
H	3.941700	−0.410700	−2.581200
H	4.528700	−1.980200	−2.077800
C	4.654600	−0.451700	−0.528400
C	0.933600	0.256700	−1.885700
H	0.094400	−0.327800	−2.275600
H	1.620900	0.434800	−2.716200
H	0.559400	1.224500	−1.535200
C	4.542100	1.581300	0.960400
H	4.575900	0.940300	1.850200
H	3.974800	2.474700	1.241800
C	6.129900	−0.088300	−0.898900
H	6.046500	0.431700	−1.863300

C	5.943600	2.035900	0.556000
H	5.858600	2.814500	−0.210700
H	6.428600	2.522500	1.411700
C	6.870800	0.918600	0.044600
C	3.763000	1.842400	−1.380800
H	3.104500	1.516600	−2.182200
H	4.743100	2.025300	−1.826000
H	3.378900	2.799800	−1.015000
C	4.577700	−1.446300	0.649500
H	4.874300	−2.447200	0.316300
H	3.561800	−1.526400	1.047800
H	5.219300	−1.172900	1.486700
C	7.527400	0.228400	1.266400
H	6.770100	−0.138600	1.968100
H	8.145900	0.954000	1.804300
C	7.034800	−1.309100	−1.197700
H	6.474200	−2.081000	−1.734800
H	7.819700	−0.986600	−1.893700
C	8.420900	−0.919400	0.847500
C	7.746400	−1.979300	−0.002900
H	6.990100	−2.444500	0.642800
C	8.009600	1.585100	−0.747200
H	8.786300	0.872400	−1.043900
H	8.492900	2.354100	−0.134200
H	7.623200	2.067600	−1.652100
O	9.595500	−0.965500	1.140900
C	8.736700	−3.038400	−0.467700
H	8.231500	−3.804100	−1.064400
H	9.224000	−3.519200	0.383300
H	9.521700	−2.581800	−1.078400

M06-2X/6-31G(d) Free Energy = −2700.392442

xuxuarine Aβ

C	1.221300	2.499600	2.257900
C	1.259700	4.999700	2.028000
C	2.075100	3.565800	0.223100
C	1.795200	4.916700	0.719700
C	1.675500	2.374800	0.986800
C	1.116200	3.800800	2.901800
H	0.963200	1.648800	2.877300
O	0.945500	3.984400	4.100000
C	2.778200	3.370700	−0.923300
H	3.104500	4.229700	−1.501200
C	1.717900	1.004700	0.293700
C	2.801900	0.923500	−0.789800
C	3.211900	2.070400	−1.369300
H	3.921200	2.056700	−2.187900
C	0.326000	0.882800	−0.403200
H	−0.445500	1.243600	0.284700
H	0.093900	−0.155900	−0.647400
H	0.286400	1.480200	−1.317600
C	1.831300	−0.125500	1.344600

H	0.845600	−0.278800	1.799500
H	2.492400	0.203700	2.152300
C	2.346600	−1.461100	0.805900
H	1.591100	−1.953200	0.182000
H	2.497200	−2.118400	1.668300
C	3.330500	−0.438500	−1.252700
C	3.662900	−1.301300	0.019400
C	4.614100	−0.309600	−2.095800
H	4.424700	0.316100	−2.974500
H	5.396700	0.195600	−1.515900
C	4.207400	−2.705100	−0.401800
H	3.341300	−3.205700	−0.856300
C	5.321300	−2.730400	−1.505700
C	5.096500	−1.669200	−2.598700
H	4.380400	−2.053000	−3.333400
H	6.036300	−1.539000	−3.150100
C	2.260000	−1.085500	−2.168400
H	1.273300	−1.136000	−1.717000
H	2.528900	−2.102200	−2.461500
H	2.169600	−0.487200	−3.081600
C	4.652700	−0.543400	0.929300
H	4.386600	0.513600	1.028200
H	5.678400	−0.575500	0.564200
H	4.645300	−0.975700	1.936000
C	6.747600	−2.540400	−0.929900
H	6.896400	−1.517000	−0.566800
H	7.484600	−2.715600	−1.720300
C	4.608200	−3.620200	0.782100
H	3.908100	−3.501600	1.615400
H	4.492700	−4.662400	0.457700
C	6.042100	−3.482500	1.338100
C	7.036700	−3.513100	0.193200
C	5.305400	−4.109900	−2.186700
H	4.310100	−4.336000	−2.585900
H	5.592100	−4.916800	−1.504500
H	6.015600	−4.124600	−3.021100
C	6.344200	−4.558500	2.372500
H	5.635400	−4.503700	3.204400
H	7.357600	−4.450100	2.764800
H	6.272700	−5.552000	1.919500
H	6.149100	−2.492300	1.800300
O	7.974500	−4.279100	0.159300
O	0.221800	5.481100	−0.402100
O	−0.853300	4.852700	1.873000
C	−0.695000	4.608700	−0.429200
C	−1.304200	4.273700	0.850800
C	−2.302500	3.247800	0.894900
H	−2.743200	3.051900	1.865300
C	−2.708100	2.616000	−0.248700
C	−2.065900	2.927300	−1.510200
C	−1.038700	3.849600	−1.608500
C	−0.268000	4.151600	−2.863200

H	0.116800	3.232700	−3.315000
H	−0.905900	4.614300	−3.620100
H	0.556100	4.824700	−2.622000
C	−3.915800	1.665400	−0.210100
C	−2.424100	2.094100	−2.686400
O	−2.095600	2.368900	−3.830800
C	−3.131600	0.829900	−2.411200
H	−3.084700	0.132200	−3.239500
C	−3.804700	0.566100	−1.282500
C	−4.090600	1.084500	1.216100
H	−4.578300	1.838500	1.844300
H	−3.102700	0.918500	1.656200
C	−4.553300	−0.765300	−1.110200
C	−4.875900	−0.223500	1.305500
H	−5.941900	−0.062100	1.109100
H	−4.810500	−0.565600	2.343500
C	−4.318600	−1.295900	0.349300
C	−5.139100	2.578000	−0.521300
H	−5.075600	3.476600	0.099400
H	−6.085500	2.084400	−0.291600
H	−5.147700	2.880200	−1.572600
C	−4.066700	−1.843300	−2.097600
H	−2.993800	−2.025100	−1.955500
H	−4.187200	−1.492800	−3.127300
C	−5.072500	−2.647400	0.567900
H	−6.135300	−2.369800	0.541800
C	−4.866700	−3.138200	−1.959600
H	−5.891400	−2.962100	−2.305900
H	−4.456000	−3.892300	−2.642700
C	−4.906800	−3.739300	−0.542100
C	−6.045700	−0.500400	−1.431700
H	−6.471100	0.324200	−0.865500
H	−6.672400	−1.374400	−1.241700
H	−6.136700	−0.246800	−2.493500
C	−2.804700	−1.421500	0.625400
H	−2.620800	−1.484700	1.703900
H	−2.256300	−0.551200	0.251900
H	−2.355800	−2.298800	0.160000
C	−3.642300	−4.614500	−0.348700
H	−2.729000	−4.040800	−0.544200
H	−3.663200	−5.445100	−1.061800
C	−4.868800	−3.273700	1.969400
H	−4.839300	−2.495500	2.738800
H	−5.756300	−3.874900	2.204200
C	−3.571500	−5.203100	1.043600
C	−3.643600	−4.190900	2.171100
H	−2.730500	−3.585800	2.090000
C	−6.111300	−4.694900	−0.476100
H	−6.138400	−5.272700	0.453800
H	−6.064400	−5.413500	−1.302000
H	−7.053200	−4.141400	−0.562700
O	−3.497300	−6.396400	1.239000

C	−3.677400	−4.872200	3.532700
H	−3.707700	−4.129300	4.335500
H	−2.801700	−5.509600	3.673700
H	−4.562400	−5.510500	3.615600
O	1.268500	6.152000	2.670000
H	0.964500	5.939000	3.580200
C	2.579800	6.107000	0.233600
H	2.089200	7.018300	0.575600
H	2.622400	6.137100	−0.855600
H	3.598500	6.081900	0.635500

M06-2X/6-31G(d) Free Energy = −2700.391653

Exo Channel

Cangorosin A

H	7.189700	−2.082100	1.273000
C	6.404500	−2.810000	1.087000
C	4.468400	−4.781000	0.572000
C	5.333000	−2.508900	0.250200
C	6.493700	−4.058000	1.684600
C	5.546900	−5.045200	1.409700
C	4.334900	−3.483500	0.028000
O	7.498600	−4.437100	2.535300
H	8.098900	−3.693500	2.680900
O	5.672700	−6.283100	1.963700
H	6.477500	−6.281400	2.505600
C	3.533300	−5.929900	0.305000
H	4.115600	−6.826200	0.071700
H	2.842700	−5.724800	−0.510900
H	2.937500	−6.150100	1.196100
C	5.320900	−1.233300	−0.595500
C	3.866000	−0.687000	−0.589000
H	3.631600	−0.550100	0.473600
C	3.178600	−3.114100	−0.784800
H	2.726300	−3.862100	−1.425300
C	2.882600	−1.756700	−1.023600
H	2.247600	−1.543800	−1.879600
O	1.669000	−3.798300	0.602700
O	1.524800	−1.260600	0.239400
C	0.436800	−1.912400	0.113100
C	−1.812600	−3.550100	−0.310100
C	−0.776600	−1.339300	−0.376400
C	0.528600	−3.345300	0.314000
C	−0.633300	−4.165700	0.049300
C	−1.888800	−2.114600	−0.541800
H	−0.783600	−0.269300	−0.558800
C	−0.507500	−5.650300	0.263000
H	−0.795000	−6.212100	−0.632300
H	−1.143900	−5.994700	1.085600
H	0.527300	−5.895100	0.507400
C	−3.047100	−4.329000	−0.459600
H	−2.968800	−5.409400	−0.534600
C	−4.246000	−3.734200	−0.458000

H	−5.145500	−4.338300	−0.541400
C	−4.355100	−2.238000	−0.296200
H	−4.112400	−2.049800	0.760900
C	−3.202300	−1.547300	−1.088300
C	−5.783700	−1.666600	−0.517400
C	−3.300900	−0.026400	−0.891300
H	−2.592400	0.475500	−1.562100
H	−2.990500	0.229200	0.129200
C	−4.704400	0.534100	−1.150500
H	−4.966700	0.426500	−2.210400
H	−4.666900	1.611600	−0.957400
C	−5.786000	−0.113200	−0.257700
C	−3.181800	−1.850600	−2.605600
H	−2.216900	−1.528800	−3.010200
H	−3.959100	−1.310500	−3.148200
H	−3.296000	−2.918200	−2.810100
C	−6.750100	−2.288000	0.508800
H	−6.393800	−2.062700	1.520300
H	−6.749900	−3.381600	0.429200
C	−7.200800	0.434500	−0.633100
H	−7.279500	0.214000	−1.705900
C	−8.193500	−1.808000	0.312600
H	−8.782200	−2.087400	1.195300
H	−8.630500	−2.384100	−0.510300
C	−8.423100	−0.292400	0.034500
C	−5.416700	0.226300	1.201100
H	−5.114900	1.275200	1.251400
H	−4.570100	−0.354400	1.573700
H	−6.234900	0.081400	1.904300
C	−6.275800	−2.025700	−1.938000
H	−5.865000	−1.372800	−2.708100
H	−7.361700	−1.965800	−2.027400
H	−5.987400	−3.050700	−2.194500
C	−8.857800	0.380500	1.358000
H	−8.114600	0.220800	2.145000
H	−9.770800	−0.124200	1.699800
C	−7.381900	1.979200	−0.585100
H	−6.454100	2.484200	−0.867500
H	−8.099200	2.232900	−1.376300
C	−9.137900	1.874500	1.238100
H	−9.415400	2.279500	2.216500
H	−9.991800	2.042400	0.570100
C	−7.943900	2.657500	0.686700
C	−9.607500	−0.205100	−0.949000
H	−9.860100	0.824100	−1.219600
H	−10.502000	−0.664900	−0.511900
H	−9.375800	−0.740200	−1.877100
C	−8.388400	4.085400	0.311300
H	−7.560500	4.656400	−0.117000
H	−8.763200	4.621100	1.190500
H	−9.196800	4.033300	−0.425000
C	−6.906700	2.834500	1.788600

O	−7.030400	2.491600	2.939000
O	−5.821500	3.506800	1.348900
C	−4.839700	3.777700	2.348100
H	−5.272000	4.376200	3.153000
H	−4.044100	4.324100	1.843800
H	−4.457900	2.844200	2.769900
C	6.244900	−0.146700	−0.024100
H	7.291000	−0.460700	−0.133000
H	6.059500	−0.044000	1.053000
C	3.665300	0.694800	−1.291700
C	6.075000	1.218600	−0.698900
H	6.419400	1.170700	−1.739700
H	6.747800	1.919700	−0.192500
C	4.628400	1.755600	−0.634400
C	5.866500	−1.675300	−1.975400
H	5.136100	−2.282100	−2.519700
H	6.757700	−2.291100	−1.817400
H	6.156500	−0.835100	−2.606800
C	4.521000	3.084700	−1.452800
H	4.870300	2.793700	−2.452500
C	2.227400	1.210600	−1.082100
H	1.506900	0.491600	−1.486800
H	2.011900	1.258900	−0.011200
C	3.074800	3.654800	−1.665400
C	1.974600	2.559000	−1.767800
H	1.771400	2.370100	−2.827500
H	1.038300	2.977000	−1.376100
C	3.903800	0.556600	−2.815700
H	3.534700	−0.407700	−3.180500
H	4.953900	0.624700	−3.100100
H	3.383600	1.330200	−3.383000
C	4.299400	1.976000	0.855100
H	4.133500	1.042800	1.396900
H	3.419200	2.595400	1.017700
H	5.149600	2.467100	1.333900
C	5.507900	4.222400	−1.060600
H	6.464200	3.807100	−0.731300
H	5.735600	4.768100	−1.985400
C	2.669500	4.656200	−0.558200
H	2.521500	4.148500	0.399800
H	1.691000	5.072600	−0.831800
C	5.073300	5.317700	−0.057500
C	3.655000	5.803500	−0.364100
H	3.313000	6.454200	0.446800
H	3.695700	6.418700	−1.271000
C	3.062600	4.409300	−3.010100
H	3.275900	3.721600	−3.836400
H	3.800500	5.215600	−3.056400
H	2.074800	4.851000	−3.188600
C	6.063100	6.494200	−0.174400
H	6.006300	6.919800	−1.181400
H	7.089200	6.165900	0.010700

H	5.815900	7.284800	0.542700
C	5.147900	4.874100	1.398000
O	4.264200	4.970200	2.214200
O	6.377300	4.414100	1.718100
C	6.538900	4.048300	3.087100
H	7.573200	3.722200	3.188200
H	6.336300	4.903200	3.735700
H	5.851900	3.238700	3.348500

M06-2X/6-31G(d) Free Energy = −2935.01871

cangorosin Aβ

H	−6.616700	3.286800	−0.137700
C	−5.709600	3.592400	0.377400
C	−3.397300	4.492600	1.692300
C	−4.529200	2.862400	0.240200
C	−5.741300	4.730400	1.167200
C	−4.590900	5.187200	1.821700
C	−3.375600	3.312300	0.912100
O	−6.858700	5.498600	1.366200
H	−7.593700	5.145200	0.847000
O	−4.653100	6.319900	2.576500
H	−5.555900	6.668200	2.505800
C	−4.425300	1.677000	−0.722900
C	−3.477600	0.633000	−0.045000
H	−3.892100	0.524200	0.971200
C	−2.144200	2.555700	0.802300
H	−1.306100	2.865700	1.413600
C	−2.136700	1.249700	0.289100
H	−1.386800	0.580500	0.695500
O	−1.180200	3.812100	−0.724700
O	−1.072800	1.291300	−1.367200
C	0.029700	1.917400	−1.272500
C	2.396200	3.406200	−0.878400
C	1.304800	1.272000	−1.358100
C	−0.035900	3.334300	−0.938700
C	1.196600	4.059300	−0.711200
C	2.457000	1.990900	−1.224000
H	1.307200	0.215500	−1.606000
C	1.092000	5.508500	−0.318400
H	1.604900	6.160100	−1.034400
H	1.537000	5.689000	0.666300
H	0.041700	5.803000	−0.286900
C	3.665700	4.108700	−0.663700
H	3.655600	5.194300	−0.642000
C	4.802700	3.435700	−0.451800
H	5.724000	3.982100	−0.268300
C	4.804400	1.927100	−0.426900
H	4.317500	1.658500	0.523400
C	3.832700	1.391700	−1.524800
C	6.220100	1.286500	−0.385000
C	3.820900	−0.144500	−1.477400
H	3.258300	−0.531400	−2.336100

H	3.282700	−0.477200	−0.581000
C	5.222700	−0.764600	−1.484000
H	5.722200	−0.565700	−2.440500
H	5.101300	−1.852000	−1.427300
C	6.097000	−0.282200	−0.306200
C	4.169300	1.840300	−2.966900
H	3.307800	1.625400	−3.607000
H	5.024700	1.303100	−3.379300
H	4.374900	2.912000	−3.023600
C	6.950400	1.735900	0.895400
H	6.361000	1.433200	1.768300
H	7.016200	2.829500	0.943100
C	7.534900	−0.882800	−0.411400
H	7.872000	−0.559300	−1.405300
C	8.377600	1.180000	0.976600
H	8.760600	1.334500	1.993100
H	9.016500	1.799900	0.338600
C	8.596800	−0.314200	0.596800
C	5.385500	−0.734800	0.986200
H	5.005900	−1.749300	0.843400
H	4.520600	−0.117400	1.237900
H	6.032400	−0.742500	1.861100
C	7.037700	1.751000	−1.612600
H	6.800900	1.192100	−2.518200
H	8.113100	1.648700	−1.458200
H	6.842500	2.807600	−1.824600
C	8.674400	−1.142300	1.901600
H	7.778800	−1.001300	2.514100
H	9.507600	−0.743900	2.495400
C	7.634700	−2.432300	−0.501900
H	6.785800	−2.841100	−1.056800
H	8.514300	−2.652700	−1.120300
C	8.898900	−2.635100	1.682900
H	8.912600	−3.153100	2.646900
H	9.878600	−2.798900	1.218300
C	7.838800	−3.271900	0.781400
C	9.976700	−0.387900	−0.087800
H	10.252400	−1.404300	−0.383200
H	10.756100	−0.014100	0.586900
H	9.990500	0.232200	−0.991400
C	8.296100	−4.684300	0.363200
H	7.570200	−5.151100	−0.307600
H	8.424000	−5.327800	1.240700
H	9.259300	−4.618300	−0.152800
C	6.559000	−3.486300	1.579000
O	6.417200	−3.279900	2.759600
O	5.581700	−4.024400	0.818200
C	4.376200	−4.328000	1.517800
H	4.570700	−5.046700	2.317000
H	3.699200	−4.749500	0.776100
H	3.951200	−3.421100	1.956100
C	−2.155000	5.024600	2.354000

H	−1.342100	5.086000	1.622600
H	−1.823800	4.374600	3.172200
H	−2.336100	6.017600	2.766100
C	−5.808700	1.045800	−0.985400
H	−6.365900	1.695700	−1.672400
H	−6.389400	1.013000	−0.056100
C	−3.481300	−0.821100	−0.614200
C	−5.745800	−0.364200	−1.577300
H	−5.281000	−0.341200	−2.571200
H	−6.773700	−0.714300	−1.726600
C	−4.972400	−1.336600	−0.663800
C	−3.904100	2.280000	−2.049500
H	−2.865400	2.603000	−1.961900
H	−4.519200	3.154600	−2.289100
H	−3.998000	1.582000	−2.885300
C	−5.025100	−2.786100	−1.255900
H	−4.769900	−2.649000	−2.313600
C	−2.712900	−1.769600	0.323200
H	−1.686900	−1.412400	0.469000
H	−3.180600	−1.791100	1.316000
C	−3.975100	−3.814700	−0.701200
C	−2.630500	−3.171100	−0.281100
H	−1.967900	−3.121900	−1.151900
H	−2.134800	−3.848100	0.426600
C	−2.795600	−0.881700	−1.998200
H	−1.712600	−0.859300	−1.867100
H	−3.039600	−0.031900	−2.630400
H	−3.044600	−1.790900	−2.548400
C	−5.653500	−1.324300	0.719200
H	−5.487100	−0.407300	1.286700
H	−5.346600	−2.153100	1.355700
H	−6.735600	−1.399700	0.572400
C	−6.446900	−3.424900	−1.328100
H	−7.207100	−2.649800	−1.466100
H	−6.473400	−4.019000	−2.250000
C	−4.513300	−4.615200	0.505600
H	−4.629400	−3.973300	1.385600
H	−3.751100	−5.358300	0.774700
C	−6.941400	−4.396000	−0.223100
C	−5.824700	−5.339800	0.230200
H	−6.149600	−5.877200	1.126700
H	−5.673700	−6.092400	−0.553000
C	−3.654400	−4.809200	−1.833100
H	−3.172500	−4.290700	−2.669800
H	−4.545800	−5.308100	−2.225200
H	−2.965400	−5.584900	−1.477600
C	−8.108600	−5.219600	−0.804100
H	−7.744900	−5.827600	−1.638500
H	−8.909900	−4.570500	−1.166500
H	−8.524200	−5.895100	−0.048100
C	−7.513600	−3.687200	0.999600
O	−7.156500	−3.844600	2.141800

O	−8.547200	−2.882400	0.674400
C	−9.142100	−2.200000	1.775900
H	−9.965700	−1.622500	1.358000
H	−9.506700	−2.914800	2.516400
H	−8.411500	−1.539800	2.251700

M06-2X/6-31G(d) Free Energy = −2935.020127

isocangorosin A

H	5.158300	4.569500	−0.186800
C	4.077200	4.467500	−0.224100
C	1.272500	4.312900	−0.275700
C	3.450500	3.308400	0.230900
C	3.323300	5.520400	−0.716700
C	1.928900	5.454600	−0.721000
C	2.046800	3.217400	0.168300
O	3.850900	6.691200	−1.195800
H	4.816200	6.650600	−1.161100
O	1.200000	6.513200	−1.173000
H	1.825300	7.206700	−1.436300
C	4.229000	2.233800	0.992800
C	3.671500	0.839600	0.571500
H	3.791100	0.807500	−0.519900
C	1.409500	1.959500	0.564100
H	0.432600	2.004700	1.032400
C	2.168600	0.786500	0.748200
H	1.752000	0.033500	1.411100
O	1.799500	−0.364000	−0.819700
O	0.399400	1.736800	−1.244800
C	−0.221800	0.640800	−1.294400
C	−1.455900	−1.894900	−1.157500
C	−1.647700	0.528600	−1.419300
C	0.564200	−0.555500	−1.065700
C	−0.089100	−1.834000	−0.973800
C	−2.248300	−0.695700	−1.398700
H	−2.201200	1.446300	−1.589800
C	0.760400	−3.052600	−0.724100
H	1.814900	−2.795100	−0.829900
H	0.534900	−3.854800	−1.433500
H	0.604400	−3.453300	0.284500
C	−2.170100	−3.171200	−1.062300
H	−1.596000	−4.090800	−1.117200
C	−3.491600	−3.213200	−0.853500
H	−3.987800	−4.174900	−0.754400
C	−4.282500	−1.936600	−0.710300
H	−4.007200	−1.541900	0.280100
C	−3.734800	−0.877300	−1.716700
C	−5.824300	−2.131500	−0.683300
C	−4.527500	0.429100	−1.550400
H	−4.254200	1.125300	−2.353100
H	−4.237700	0.915000	−0.610700
C	−6.047300	0.227800	−1.566400
H	−6.375600	−0.114800	−2.555500

H	−6.509000	1.210000	−1.418800
C	−6.535400	−0.743300	−0.469000
C	−3.791000	−1.303200	−3.203100
H	−3.166100	−0.618400	−3.785000
H	−4.801800	−1.249600	−3.610600
H	−3.412300	−2.317300	−3.352700
C	−6.207000	−3.017800	0.517100
H	−5.858700	−2.542100	1.441100
H	−5.691100	−3.984000	0.465900
C	−8.076900	−0.967000	−0.582400
H	−8.206700	−1.312100	−1.616800
C	−7.713200	−3.297500	0.576200
H	−7.954800	−3.728000	1.556100
H	−7.936800	−4.093000	−0.142800
C	−8.680900	−2.104900	0.316700
C	−6.159800	−0.106100	0.884600
H	−6.393300	0.960700	0.846400
H	−5.094200	−0.179200	1.112000
H	−6.696000	−0.533200	1.729800
C	−6.285400	−2.834900	−1.981300
H	−6.371500	−2.154900	−2.828700
H	−7.259100	−3.314500	−1.869000
H	−5.572700	−3.616300	−2.266200
C	−9.173100	−1.578600	1.685600
H	−8.332300	−1.292900	2.325300
H	−9.669500	−2.412200	2.199200
C	−8.967100	0.309100	−0.530400
H	−8.451200	1.155600	−0.992100
H	−9.831600	0.111700	−1.177400
C	−10.147900	−0.410000	1.593700
H	−10.427100	−0.080200	2.599200
H	−11.070800	−0.732200	1.096800
C	−9.580100	0.777000	0.811700
C	−9.898900	−2.687000	−0.428100
H	−10.658600	−1.933500	−0.655900
H	−10.376200	−3.470100	0.173300
H	−9.588400	−3.137300	−1.377900
C	−10.710000	1.778800	0.500600
H	−10.338100	2.625500	−0.081900
H	−11.155200	2.161600	1.425700
H	−11.496500	1.276200	−0.071400
C	−8.599700	1.538000	1.695400
O	−8.358500	1.299700	2.853900
O	−8.060800	2.593700	1.048900
C	−7.183600	3.395500	1.837800
H	−7.698800	3.757900	2.729800
H	−6.883000	4.225200	1.199700
H	−6.311400	2.813300	2.147500
C	−0.232800	4.345100	−0.307100
H	−0.592100	4.244700	−1.335400
H	−0.587800	5.305300	0.077400
H	−0.679500	3.538100	0.272300

C	5.730500	2.271100	0.668400
H	6.167600	3.195700	1.066500
H	5.861200	2.306800	−0.421000
C	4.447700	−0.380800	1.168900
C	6.515200	1.085200	1.236600
H	6.536700	1.137600	2.332600
H	7.555000	1.194000	0.909100
C	5.970100	−0.282000	0.772600
C	4.028200	2.609100	2.482600
H	3.010900	2.383800	2.818600
H	4.181500	3.687200	2.593400
H	4.730200	2.109600	3.150400
C	6.740700	−1.432900	1.500100
H	6.585400	−1.204500	2.563000
C	3.919300	−1.701100	0.579800
H	2.853900	−1.803700	0.806100
H	3.979700	−1.661200	−0.512600
C	6.178700	−2.884000	1.301500
C	4.633400	−2.939100	1.137400
H	4.190800	−3.178400	2.110600
H	4.385600	−3.798700	0.500900
C	4.248800	−0.442900	2.703900
H	3.237800	−0.124500	2.977600
H	4.945500	0.183400	3.260200
H	4.376300	−1.456000	3.088100
C	6.159100	−0.344500	−0.756500
H	5.431600	0.260500	−1.301000
H	6.091600	−1.353500	−1.157800
H	7.144100	0.054500	−1.009600
C	8.290800	−1.423100	1.367500
H	8.669300	−0.398100	1.327100
H	8.685500	−1.837300	2.304000
C	6.829600	−3.608500	0.100000
H	6.508200	−3.169700	−0.849600
H	6.454400	−4.640300	0.094300
C	8.977000	−2.248200	0.253100
C	8.354200	−3.641800	0.143600
H	8.740800	−4.139900	−0.751100
H	8.688700	−4.230300	1.006400
C	6.509400	−3.691800	2.573000
H	6.017600	−3.248000	3.446300
H	7.581200	−3.735400	2.786800
H	6.147100	−4.721900	2.472800
C	10.473700	−2.374700	0.602500
H	10.583100	−2.921600	1.544500
H	10.936500	−1.390600	0.712500
H	11.010300	−2.927600	−0.176400
C	8.941300	−1.572900	−1.111900
O	8.573500	−2.079100	−2.143700
O	9.456900	−0.324800	−1.065800
C	9.529600	0.337200	−2.326900
H	9.975200	1.310800	−2.127500

H	10.147100	−0.236200	−3.021700
H	8.530700	0.451000	−2.756400

M06-2X/6-31G(d) Free Energy = −2935.019126

isocangorin Aβ

H	4.951200	4.263400	1.102600
C	4.123300	4.152700	0.407000
C	1.938500	3.971400	−1.348300
C	3.563900	2.900500	0.155000
C	3.621500	5.282400	−0.219100
C	2.528400	5.199800	−1.090600
C	2.478800	2.813400	−0.739300
O	4.118800	6.546200	−0.038100
H	4.823700	6.526400	0.623400
O	2.046300	6.334200	−1.671700
H	2.560300	7.080600	−1.325200
C	3.998600	1.655300	0.931400
C	3.937100	0.455500	−0.069100
H	4.504700	0.812400	−0.944200
C	1.909500	1.518400	−1.044400
C	2.564200	0.328500	−0.693900
O	1.404400	−0.603300	0.575800
O	0.135600	1.658100	0.301700
C	−0.528300	0.610900	0.110900
C	−1.806100	−1.843500	−0.504300
C	−1.883500	0.597500	−0.374900
C	0.179000	−0.659900	0.231400
C	−0.469300	−1.881900	−0.164400
C	−2.520700	−0.577400	−0.634100
H	−2.367200	1.561400	−0.494300
C	0.330100	−3.155700	−0.095400
H	0.100400	−3.730100	0.809500
H	1.396000	−2.918900	−0.075000
H	0.136100	−3.800500	−0.956800
C	−2.564900	−3.075700	−0.730100
H	−2.019200	−4.005200	−0.858600
C	−3.904000	−3.079400	−0.722700
H	−4.436100	−4.016500	−0.862700
C	−4.672600	−1.804900	−0.478900
H	−4.545900	−1.599900	0.595400
C	−3.946800	−0.622400	−1.191800
C	−6.205300	−1.917400	−0.717500
C	−4.716000	0.679400	−0.917300
H	−4.303900	1.484100	−1.538700
H	−4.556400	0.983800	0.124200
C	−6.220600	0.569100	−1.189600
H	−6.403200	0.424500	−2.261800
H	−6.671200	1.534700	−0.934700
C	−6.901100	−0.548900	−0.369800
C	−3.787600	−0.790700	−2.721600
H	−3.073200	−0.043600	−3.081600
H	−4.726100	−0.630100	−3.254200

H	−3.406100	−1.780200	−2.986200
C	−6.794900	−2.971200	0.239700
H	−6.572900	−2.682000	1.272900
H	−6.310000	−3.943600	0.092500
C	−8.409800	−0.667000	−0.758900
H	−8.376500	−0.837600	−1.843300
C	−8.302100	−3.169200	0.035600
H	−8.702600	−3.732900	0.887400
H	−8.440400	−3.830100	−0.827100
C	−9.182500	−1.899400	−0.165200
C	−6.724300	−0.176500	1.116600
H	−6.932700	0.888800	1.237900
H	−5.705100	−0.332700	1.476000
H	−7.384400	−0.724400	1.786500
C	−6.478700	−2.367800	−2.171500
H	−6.433000	−1.549400	−2.890100
H	−7.463300	−2.824500	−2.284000
H	−5.740800	−3.114100	−2.485100
C	−9.873300	−1.568300	1.178600
H	−9.138800	−1.429400	1.977700
H	−10.468700	−2.444000	1.467900
C	−9.263800	0.628300	−0.643200
H	−8.660100	1.508100	−0.881800
H	−10.019700	0.574400	−1.437400
C	−10.788000	−0.349100	1.127100
H	−11.217600	−0.166000	2.117000
H	−11.626300	−0.540800	0.446300
C	−10.068500	0.913500	0.646600
C	−10.280900	−2.288200	−1.174400
H	−10.971800	−1.467900	−1.388700
H	−10.870900	−3.129600	−0.791400
H	−9.835800	−2.598300	−2.126700
C	−11.104000	2.012500	0.334200
H	−10.618200	2.918600	−0.036900
H	−11.681800	2.269000	1.229100
H	−11.801900	1.650400	−0.427600
C	−9.220100	1.472900	1.781100
O	−9.185300	1.053800	2.912300
O	−8.539300	2.574300	1.398500
C	−7.774100	3.194700	2.430400
H	−8.426500	3.520100	3.243800
H	−7.282300	4.047300	1.964500
H	−7.035800	2.494800	2.830600
C	0.721200	3.902600	−2.230700
H	−0.080700	3.369400	−1.709200
H	0.930000	3.370800	−3.166100
H	0.375400	4.905200	−2.483100
C	5.432400	1.815400	1.479500
H	5.402600	2.482800	2.350300
H	6.063100	2.312700	0.733200
C	4.670100	−0.858900	0.349000
C	6.098300	0.499900	1.887600

H	5.565300	0.045300	2.732300
H	7.106700	0.730600	2.250100
C	6.163100	−0.501000	0.715900
C	3.018600	1.567500	2.127600
H	2.020400	1.264400	1.805000
H	2.946900	2.561200	2.583200
H	3.374300	0.884400	2.903300
C	6.941000	−1.788500	1.156600
H	6.503700	−2.043100	2.129500
C	4.723300	−1.847100	−0.829600
H	3.710100	−2.068400	−1.186200
H	5.263700	−1.405200	−1.676800
C	6.754100	−3.068100	0.264800
C	5.357500	−3.168000	−0.396900
H	4.665400	−3.663300	0.293100
H	5.437400	−3.838000	−1.262600
C	3.940900	−1.564300	1.515900
H	3.070700	−2.100500	1.134200
H	3.562900	−0.869800	2.260500
H	4.577200	−2.289300	2.026900
C	6.918100	0.179000	−0.443400
H	6.355000	0.973000	−0.935900
H	7.228900	−0.519400	−1.219300
H	7.817500	0.652300	−0.037700
C	8.446300	−1.568200	1.506500
H	8.603100	−0.560200	1.903600
H	8.671200	−2.242400	2.342400
C	7.817000	−3.165400	−0.851300
H	7.676500	−2.379800	−1.601500
H	7.657800	−4.115800	−1.377200
C	9.558600	−1.857100	0.461800
C	9.251800	−3.124100	−0.339800
H	9.950000	−3.196500	−1.179500
H	9.439200	−3.990800	0.305400
C	6.898200	−4.299200	1.178800
H	6.094000	−4.315800	1.923100
H	7.848300	−4.317600	1.721100
H	6.830700	−5.223300	0.591600
C	10.888100	−2.036500	1.222500
H	10.816900	−2.908900	1.879800
H	11.118600	−1.157200	1.829200
H	11.716500	−2.203700	0.525100
C	9.799900	−0.706200	−0.508800
O	9.770900	−0.777300	−1.713500
O	10.132700	0.432400	0.135400
H	1.147200	1.472600	−1.812200
H	2.421300	−0.507700	−1.369800
C	10.391400	1.545200	−0.717400
H	10.653100	2.371600	−0.057900
H	11.213400	1.319700	−1.400000
H	9.501600	1.787900	−1.304900

M06-2X/6-31G(d) Free Energy = −2935.022663

isoxuxuarine Aα

C	3.609700	−3.298600	0.488800
C	1.273400	−3.600500	−0.340900
C	2.811900	−2.159700	−1.535300
C	1.517500	−2.859400	−1.514900
C	3.842300	−2.436400	−0.529400
C	2.326800	−3.970600	0.632000
H	4.355000	−3.549000	1.234600
O	2.054800	−4.829100	1.463600
C	3.022100	−1.152200	−2.416600
H	2.236800	−0.896400	−3.122100
C	5.247900	−1.872600	−0.775000
C	5.229700	−0.546300	−1.547300
C	4.182000	−0.294800	−2.357400
H	4.151400	0.600300	−2.966400
C	5.908200	−2.953400	−1.689400
H	5.436800	−2.966200	−2.676400
H	5.776300	−3.934400	−1.223200
H	6.977500	−2.779600	−1.813500
C	6.029300	−1.791400	0.559400
H	6.409000	−2.789100	0.807400
H	5.326200	−1.528100	1.355300
C	7.189000	−0.794600	0.596800
H	8.051600	−1.166100	0.032900
H	7.518100	−0.731600	1.639000
C	6.412000	0.423600	−1.442800
C	6.777700	0.597500	0.077200
C	6.073100	1.811800	−2.021700
H	5.788200	1.719700	−3.075500
H	5.204300	2.232200	−1.500200
C	7.972000	1.592900	0.239400
H	8.827700	1.058900	−0.198000
C	7.885100	2.941100	−0.552700
C	7.265000	2.765400	−1.951500
H	8.038900	2.424000	−2.648100
H	6.959400	3.753100	−2.319500
C	7.581000	−0.141200	−2.287800
H	7.884600	−1.143100	−1.995700
H	8.471300	0.487800	−2.229900
H	7.269700	−0.184900	−3.337300
C	5.530400	1.036800	0.872800
H	4.645500	0.458000	0.591400
H	5.274800	2.086200	0.726900
H	5.691500	0.877600	1.944600
C	7.088900	4.035700	0.202300
H	6.021200	3.792700	0.245800
H	7.186900	4.987800	−0.329300
C	8.386500	1.862100	1.707200
H	8.313700	0.945800	2.301400
H	9.451600	2.127100	1.716100
C	7.640600	2.983600	2.462400
C	7.612600	4.236000	1.608300

C	9.309100	3.492900	−0.744000
H	9.953200	2.751000	−1.229700
H	9.776100	3.785100	0.202400
H	9.282900	4.385600	−1.378900
C	8.266000	3.246800	3.825700
H	9.301800	3.580500	3.710700
H	8.258300	2.338500	4.435700
H	7.727300	4.033700	4.357900
O	0.133200	−4.273200	−0.234900
H	0.225200	−4.796200	0.595100
C	0.873700	−3.320900	−2.799400
H	1.417800	−4.188800	−3.189300
H	0.888500	−2.533300	−3.553600
H	6.591800	2.686500	2.592800
O	8.012700	5.309000	2.004000
C	−0.293700	−1.836900	0.805300
C	−0.672500	−1.476200	−0.550200
C	−2.052100	−1.424300	−0.900300
H	−2.285300	−1.195200	−1.934000
C	−3.018300	−1.591900	0.056600
C	−2.639000	−1.971300	1.398900
C	−1.319200	−2.170500	1.767600
C	−0.855400	−2.644000	3.116100
H	−1.297300	−3.612400	3.359700
H	−1.176800	−1.963300	3.909900
H	0.232800	−2.721100	3.112700
C	−4.500600	−1.550900	−0.349600
C	−3.720100	−2.081000	2.414400
O	−3.561200	−2.586000	3.514900
C	−5.012700	−1.454000	2.075300
H	−5.635500	−1.275600	2.944300
C	−5.406300	−1.152300	0.830500
C	−4.682600	−0.624900	−1.579900
H	−4.396400	−1.178600	−2.481300
H	−3.978600	0.208400	−1.500900
C	−6.782000	−0.516500	0.572300
C	−6.083400	−0.047100	−1.777700
H	−6.785900	−0.813400	−2.124000
H	−6.014600	0.683900	−2.589900
C	−6.617000	0.620900	−0.496500
C	−4.827100	−3.012400	−0.776200
H	−4.058900	−3.356800	−1.475000
H	−5.792900	−3.087100	−1.279800
H	−4.832600	−3.679600	0.090700
C	−7.385800	0.105100	1.846900
H	−6.709100	0.871200	2.245400
H	−7.483900	−0.656800	2.626800
C	−7.993500	1.299900	−0.789800
H	−8.572700	0.520000	−1.303800
C	−8.778400	0.681200	1.592500
H	−9.474300	−0.141900	1.393800
H	−9.146700	1.156700	2.510500

C	−8.865800	1.706800	0.446400
C	−7.743700	−1.644000	0.120800
H	−7.377100	−2.210300	−0.731800
H	−8.729100	−1.261100	−0.152900
H	−7.875200	−2.348500	0.949200
C	−5.555600	1.637600	−0.026100
H	−5.217800	2.247600	−0.871400
H	−4.673100	1.141400	0.389100
H	−5.925000	2.314000	0.744200
C	−8.501700	3.103100	1.013200
H	−7.518700	3.090200	1.498300
H	−9.236400	3.389800	1.772600
C	−7.925400	2.468100	−1.804600
H	−7.204200	2.247800	−2.598200
H	−8.897400	2.536100	−2.310100
C	−8.511000	4.164500	−0.065000
C	−7.613400	3.875900	−1.252200
H	−6.586500	3.886500	−0.863100
C	−10.337100	1.785600	0.003000
H	−10.512800	2.582300	−0.727500
H	−10.978900	1.990800	0.867100
H	−10.660600	0.837300	−0.440900
O	−9.211500	5.151100	0.000900
C	−7.755200	4.941500	−2.331100
H	−7.083300	4.734500	−3.169600
H	−7.528200	5.932800	−1.932500
H	−8.782900	4.968400	−2.705900
O	0.944500	−1.989600	1.002400
O	0.274600	−1.320300	−1.384800
H	−0.162300	−3.612300	−2.615800

M06-2X/6-31G(d) Free Energy = −2700.387196

isoxuxuarine Aβ

C	−3.037200	−1.524800	−1.594500
C	−0.710900	−0.634700	−1.706600
C	−2.414300	0.573400	−0.478000
C	−1.027000	0.517500	−0.949900
C	−3.329400	−0.557700	−0.695100
C	−1.732400	−1.569600	−2.238600
H	−3.706600	−2.343900	−1.826600
O	−1.393300	−2.328300	−3.139100
C	−2.912100	1.679600	0.124900
H	−2.276600	2.549000	0.261700
C	−4.528300	−0.662000	0.243200
C	−5.108600	0.712800	0.609000
C	−4.285800	1.779600	0.561700
H	−4.627100	2.762400	0.863900
C	−3.887500	−1.301500	1.522100
H	−3.248100	−2.133700	1.214700
H	−4.653900	−1.689600	2.195100
H	−3.269200	−0.575200	2.056400
C	−5.585200	−1.636600	−0.320800

H	−5.237400	−2.663100	−0.157900
H	−5.648000	−1.504600	−1.405600
C	−6.989000	−1.477800	0.261900
H	−7.028600	−1.831200	1.298600
H	−7.650900	−2.138500	−0.307100
C	−6.550600	0.832400	1.113500
C	−7.486800	−0.020900	0.182400
C	−7.058500	2.286900	1.104500
H	−6.404600	2.917600	1.716000
H	−7.020600	2.693500	0.086100
C	−8.966900	0.062000	0.678300
H	−8.960700	−0.479600	1.634400
C	−9.522000	1.487300	1.020200
C	−8.468100	2.392900	1.685400
H	−8.429000	2.173900	2.758400
H	−8.813100	3.432300	1.614000
C	−6.566100	0.360300	2.589900
H	−6.117700	−0.618800	2.735300
H	−7.576800	0.313000	3.000500
H	−5.992400	1.072700	3.192700
C	−7.337100	0.443300	−1.282000
H	−6.289400	0.619800	−1.545700
H	−7.870900	1.368800	−1.495800
H	−7.719500	−0.324900	−1.963400
C	−10.086700	2.229800	−0.217200
H	−9.283700	2.528800	−0.900800
H	−10.593700	3.144100	0.108300
C	−9.982200	−0.711900	−0.198400
H	−9.545400	−1.649300	−0.557000
H	−10.821600	−1.013500	0.441300
C	−10.589800	0.027300	−1.409900
C	−11.094000	1.388500	−0.971000
C	−10.691900	1.330600	2.007300
H	−10.376500	0.779500	2.900500
H	−11.544100	0.804400	1.564400
H	−11.049400	2.315800	2.326800
C	−11.691500	−0.793700	−2.066600
H	−11.305400	−1.762100	−2.399100
H	−12.112400	−0.268100	−2.926500
H	−12.507200	−0.968400	−1.358500
H	−9.794300	0.222700	−2.141100
O	−12.227100	1.764900	−1.175800
O	−0.548400	−2.078300	−0.174200
O	−0.107300	0.255400	0.787200
C	0.642400	−1.853800	0.186400
C	0.893100	−0.527100	0.720700
C	2.226700	−0.143000	1.043200
H	2.361400	0.847900	1.461400
C	3.255600	−1.040900	0.920900
C	3.008500	−2.343300	0.343900
C	1.751800	−2.737100	−0.082900
C	1.429600	−4.025800	−0.784800

H	1.472700	−4.873000	−0.094300
H	2.158300	−4.240200	−1.568600
H	0.424300	−3.959700	−1.205700
C	4.628000	−0.707900	1.527600
C	4.181500	−3.227200	0.114500
O	4.081600	−4.408900	−0.177300
C	5.514200	−2.599400	0.187200
H	6.288900	−3.198800	−0.277000
C	5.772100	−1.433800	0.796400
C	4.833900	0.827300	1.568800
H	4.271400	1.233600	2.417300
H	4.391500	1.270300	0.671500
C	7.206300	−0.884300	0.863700
C	6.286200	1.291300	1.671000
H	6.700700	1.075100	2.662400
H	6.283000	2.382100	1.577100
C	7.172000	0.659800	0.579900
C	4.534200	−1.225700	2.994200
H	3.588100	−0.886800	3.425900
H	5.340000	−0.834300	3.618300
H	4.562500	−2.318700	3.028900
C	8.137200	−1.541300	−0.174200
H	7.750800	−1.369700	−1.186600
H	8.157300	−2.626200	−0.030200
C	8.612400	1.261500	0.653800
H	8.883200	1.184100	1.716200
C	9.573800	−1.035500	−0.039900
H	9.992200	−1.400800	0.904400
H	10.192100	−1.488200	−0.825500
C	9.742500	0.493900	−0.113300
C	7.773000	−1.238100	2.262000
H	7.135900	−0.918500	3.082500
H	8.757800	−0.799200	2.434400
H	7.874800	−2.326500	2.334300
C	6.494700	0.928200	−0.781600
H	6.175400	1.974800	−0.845400
H	5.602700	0.308600	−0.917500
H	7.146700	0.728400	−1.631600
C	9.844600	0.897600	−1.606200
H	8.983100	0.528100	−2.174200
H	10.741200	0.446200	−2.043300
C	8.687000	2.777100	0.342700
H	7.818400	3.298200	0.757700
H	9.552200	3.192200	0.875700
C	9.950500	2.396900	−1.779800
C	8.832300	3.193900	−1.136400
H	7.916700	2.913900	−1.673200
C	11.095600	0.845400	0.528900
H	11.353100	1.903100	0.409400
H	11.896300	0.262000	0.060800
H	11.089500	0.613100	1.600000
O	10.878100	2.919500	−2.358100

C	9.062500	4.692800	−1.273100
H	8.236800	5.252800	−0.823600
H	9.154400	4.980300	−2.322900
H	9.992100	4.981000	−0.772900
O	0.491100	−0.720300	−2.265600
H	0.441600	−1.508900	−2.853700
C	−0.247200	1.776300	−1.240800
H	0.801300	1.526300	−1.413200
H	−0.299400	2.476900	−0.407500
H	−0.641000	2.259300	−2.142100

M06-2X/6-31G(d) Free Energy = −2700.389684

xuxuarine Aα

C	2.380800	−0.299700	2.593100
C	0.442800	−1.605200	1.721200
C	2.695500	−2.183300	1.046400
C	1.263400	−2.536000	1.052500
C	3.212000	−1.079100	1.860300
C	0.944000	−0.522900	2.597200
H	2.732300	0.509500	3.221900
O	0.122700	0.067400	3.290600
C	3.539200	−2.805300	0.189400
H	3.150100	−3.580600	−0.464100
C	4.734300	−0.955000	2.011000
C	5.500900	−1.464100	0.780800
C	4.906600	−2.383500	−0.004800
H	5.426300	−2.813200	−0.852200
C	5.049800	−1.907300	3.208600
H	4.375400	−1.664500	4.035200
H	6.073400	−1.783900	3.563100
H	4.898600	−2.952900	2.925900
C	5.114000	0.494100	2.406800
H	4.928200	0.627200	3.478600
H	4.435900	1.182200	1.892900
C	6.552200	0.912600	2.096600
H	7.258700	0.451500	2.795400
H	6.614700	1.990500	2.276800
C	6.929200	−0.977600	0.505800
C	6.954100	0.588200	0.644300
C	7.401600	−1.345800	−0.914300
H	7.365700	−2.432000	−1.051300
H	6.719600	−0.915600	−1.658100
C	8.391100	1.135800	0.360000
H	8.990300	0.770100	1.205600
C	9.117700	0.595000	−0.918800
C	8.840200	−0.899400	−1.170200
H	9.517500	−1.501400	−0.554500
H	9.110900	−1.130100	−2.208300
C	7.886200	−1.689400	1.494900
H	7.620000	−1.544900	2.538600
H	8.918200	−1.351100	1.383600
H	7.863100	−2.766400	1.296000

C	5.891200	1.220400	−0.279000
H	4.936600	0.688000	−0.226200
H	6.184400	1.229800	−1.328400
H	5.699400	2.256100	0.022300
C	8.747700	1.381800	−2.201700
H	7.709900	1.191800	−2.498100
H	9.388700	1.054000	−3.026400
C	8.515100	2.678200	0.432300
H	7.892800	3.076800	1.239700
H	9.546200	2.922800	0.717500
C	8.204900	3.479800	−0.850500
C	8.950000	2.871100	−2.022900
C	10.636100	0.758900	−0.727800
H	10.968700	0.261000	0.190100
H	10.941700	1.809100	−0.677600
H	11.170500	0.306700	−1.570600
C	8.539300	4.955600	−0.678800
H	7.975300	5.384700	0.154800
H	8.308400	5.516600	−1.586900
H	9.607600	5.082500	−0.479000
O	−0.859800	−1.829900	1.775800
H	−1.217800	−1.102000	2.336600
C	0.843400	−3.988000	1.068300
H	−0.228700	−4.071200	0.878500
H	1.062400	−4.424700	2.048900
H	1.378500	−4.557700	0.307200
H	7.137900	3.372300	−1.085900
O	9.680400	3.516600	−2.742300
O	0.795800	−2.313700	−0.799100
O	0.516800	0.021000	0.236400
C	−0.315000	−1.723200	−1.021300
C	−0.446500	−0.387400	−0.463000
C	−1.695200	0.298400	−0.606700
H	−1.747700	1.293600	−0.180200
C	−2.724700	−0.261500	−1.313000
C	−2.580100	−1.594700	−1.854700
C	−1.431600	−2.350000	−1.675500
C	−1.242500	−3.759800	−2.163600
H	−1.221500	−3.798400	−3.255400
H	−2.074100	−4.397800	−1.856600
H	−0.302800	−4.152100	−1.772400
C	−3.968200	0.584000	−1.640400
C	−3.758800	−2.191900	−2.535500
O	−3.700200	−3.209600	−3.209000
C	−5.061700	−1.544000	−2.298600
H	−5.900900	−2.183800	−2.545500
C	−5.217200	−0.289900	−1.852800
C	−4.177600	1.661500	−0.545000
H	−3.487500	2.491700	−0.732700
H	−3.889100	1.242500	0.423400
C	−6.621300	0.304000	−1.654700
C	−5.596700	2.216300	−0.420500

H	−5.842900	2.871300	−1.263900
H	−5.613200	2.854300	0.469100
C	−6.646500	1.094700	−0.298300
C	−3.605700	1.301400	−2.974500
H	−2.610700	1.744600	−2.876300
H	−4.304100	2.106000	−3.211700
H	−3.591200	0.593500	−3.808400
C	−7.713200	−0.782200	−1.601100
H	−7.506900	−1.481200	−0.781100
H	−7.701100	−1.373600	−2.522100
C	−8.059500	1.717500	−0.053800
H	−8.148500	2.493900	−0.826500
C	−9.108800	−0.174500	−1.466100
H	−9.359700	0.353000	−2.393100
H	−9.848600	−0.980500	−1.381700
C	−9.296000	0.780900	−0.272600
C	−6.932700	1.193100	−2.884900
H	−6.170100	1.941400	−3.083300
H	−7.880700	1.724900	−2.781200
H	−7.001800	0.554400	−3.772100
C	−6.206700	0.172300	0.858600
H	−5.924300	0.770900	1.731900
H	−5.336800	−0.432600	0.585400
H	−6.984700	−0.523400	1.171300
C	−9.643500	−0.065200	0.978700
H	−8.881200	−0.830500	1.163700
H	−10.593500	−0.583800	0.813800
C	−8.198500	2.483000	1.285500
H	−7.276700	3.026700	1.515200
H	−8.964600	3.258100	1.156200
C	−9.795100	0.795900	2.213800
C	−8.595900	1.667300	2.535100
H	−7.779700	0.974700	2.779500
C	−10.528700	1.654800	−0.565000
H	−10.817100	2.273100	0.291500
H	−11.387900	1.020600	−0.810000
H	−10.342600	2.316600	−1.418400
O	−10.811800	0.811200	2.872700
C	−8.865000	2.573300	3.729200
H	−7.982300	3.175900	3.963500
H	−9.137000	1.988100	4.610300
H	−9.699900	3.247200	3.514200

M06-2X/6-31G(d) Free Energy = −2700.387647

xuxuarine Aβ

C	−2.753000	−0.588900	1.970100
C	−1.048000	−2.336900	1.482800
C	−2.854500	−1.949400	−0.076600
C	−1.653200	−2.729000	0.266400
C	−3.252900	−0.785300	0.728800
C	−1.652800	−1.404100	2.461400
H	−3.060600	0.226200	2.613400

O	−1.164100	−1.372100	3.585600
C	−3.660600	−2.306100	−1.104600
H	−3.426500	−3.188000	−1.693100
C	−4.124500	0.256900	0.035900
C	−5.157700	−0.378400	−0.906100
C	−4.868600	−1.583000	−1.437100
H	−5.534200	−2.051800	−2.151800
C	−3.073700	1.047500	−0.817900
H	−2.196600	1.249900	−0.197100
H	−3.482200	2.001700	−1.156500
H	−2.751400	0.463600	−1.684300
C	−4.749900	1.225500	1.061900
H	−3.979700	1.932400	1.390700
H	−5.046500	0.662000	1.951700
C	−5.968400	1.998400	0.557800
H	−5.678500	2.750200	−0.185500
H	−6.372100	2.555600	1.409200
C	−6.415200	0.397800	−1.311000
C	−7.041600	1.061200	−0.031200
C	−7.484500	−0.504400	−1.956700
H	−7.070300	−1.013800	−2.833100
H	−7.789600	−1.290000	−1.254200
C	−8.312800	1.884900	−0.414700
H	−7.920300	2.726900	−1.001300
C	−9.356000	1.184800	−1.350700
C	−8.690100	0.308500	−2.428800
H	−8.383700	0.941900	−3.268900
H	−9.448400	−0.370700	−2.838600
C	−5.988900	1.430800	−2.386200
H	−5.134400	2.033700	−2.090500
H	−6.795200	2.120900	−2.643100
H	−5.704700	0.892700	−3.297100
C	−7.343800	−0.022700	1.026600
H	−6.526800	−0.747100	1.107100
H	−8.246200	−0.593400	0.810700
H	−7.470000	0.436100	2.013700
C	−10.376100	0.313600	−0.575000
H	−9.900100	−0.581600	−0.158600
H	−11.160200	−0.023600	−1.260600
C	−9.030400	2.552400	0.784200
H	−8.301100	2.918400	1.513400
H	−9.542900	3.450400	0.415900
C	−10.084800	1.715300	1.539900
C	−11.040400	1.089400	0.542200
C	−10.172500	2.270600	−2.074100
H	−9.513300	2.953700	−2.621800
H	−10.784400	2.862700	−1.385800
H	−10.854800	1.808500	−2.796300
C	−10.823100	2.553400	2.574600
H	−10.121900	2.975700	3.300700
H	−11.564500	1.954100	3.107700
H	−11.356500	3.374900	2.086700

H	−9.579500	0.878000	2.039500
O	−12.241900	1.229300	0.608400
O	−0.423800	−2.152100	−1.065800
O	−0.121200	−0.435000	0.815900
C	0.776400	−1.953800	−0.665700
C	0.939300	−0.944800	0.365700
C	2.242600	−0.717400	0.907300
H	2.312700	0.009800	1.708300
C	3.334300	−1.362600	0.393600
C	3.154100	−2.375200	−0.622200
C	1.902900	−2.728800	−1.107100
C	1.649900	−3.802800	−2.128900
H	1.965600	−4.778400	−1.752500
H	2.231200	−3.631500	−3.038800
H	0.587100	−3.823600	−2.373500
C	4.717400	−1.130100	1.024600
C	4.377700	−2.994800	−1.196400
O	4.360100	−3.995900	−1.896500
C	5.652000	−2.289500	−0.957700
H	6.433100	−2.585700	−1.648500
C	5.853200	−1.395500	0.020100
C	4.782300	0.293900	1.636000
H	4.286900	0.278900	2.613600
H	4.194300	0.972000	1.011100
C	7.221900	−0.720700	0.201800
C	6.180000	0.889900	1.797900
H	6.728000	0.407500	2.614900
H	6.048400	1.933900	2.100700
C	6.993800	0.806000	0.492200
C	4.800500	−2.169700	2.180800
H	3.884500	−2.109800	2.776100
H	5.642500	−1.974400	2.847600
H	4.894100	−3.185900	1.786900
C	8.106800	−0.849500	−1.052900
H	7.600800	−0.401200	−1.917100
H	8.260100	−1.905400	−1.297700
C	8.361400	1.541900	0.672300
H	8.760300	1.142600	1.615400
C	9.484200	−0.223300	−0.839700
H	10.038500	−0.817500	−0.104400
H	10.063600	−0.296700	−1.768700
C	9.470400	1.250400	−0.394500
C	7.959300	−1.454900	1.349900
H	7.380100	−1.516700	2.267700
H	8.907000	−0.975800	1.604600
H	8.176500	−2.480100	1.030800
C	6.139500	1.434900	−0.629100
H	5.719700	2.389200	−0.291800
H	5.298600	0.793400	−0.909700
H	6.703900	1.625200	−1.541500
C	9.346400	2.139300	−1.657600
H	8.466100	1.867000	−2.251300

H	10.225500	1.991700	−2.293500
C	8.241100	3.067000	0.912600
H	7.375900	3.290400	1.545000
H	9.113700	3.388100	1.495800
C	9.274000	3.609000	−1.304700
C	8.172600	3.981100	−0.329900
H	7.229200	3.789600	−0.858200
C	10.843500	1.562700	0.225700
H	10.972500	2.627800	0.444900
H	11.641700	1.276500	−0.468400
H	10.987600	1.001600	1.156000
O	10.059700	4.419800	−1.744700
C	8.244300	5.452200	0.057500
H	7.431500	5.710900	0.742900
H	8.179800	6.092900	−0.824600
H	9.197500	5.668800	0.549600
O	0.018800	−2.993100	1.902700
H	0.212000	−2.617300	2.794100
C	−1.545700	−4.187200	−0.117800
H	−1.723400	−4.324800	−1.184800
H	−2.274600	−4.779600	0.445900
H	−0.543300	−4.554700	0.110700

M06-2X/6-31G(d) Free Energy = −2700.387592

Cartesian Coordinates of All the Stationary Points for Triterpene Dimers Included in This Study at M06_2x/6-31G* Level of Theory.

cangorosin A

H	7.697400	−0.098300	−0.059700
C	7.192900	−1.061100	−0.059700
C	5.978800	−3.580900	−0.034100
C	5.870100	−1.177200	−0.512100
C	7.883600	−2.166000	0.396400
C	7.286000	−3.431500	0.408000
C	5.285100	−2.445800	−0.501700
O	9.172400	−2.136600	0.862900
H	9.504300	−1.228500	0.850300
O	7.990900	−4.506100	0.856600
H	8.862200	−4.185800	1.139500
C	5.156300	0.059500	−1.082900
C	3.600100	−0.153300	−0.976500
H	3.420600	−0.396100	0.078300
C	3.857100	−2.635400	−0.933900
H	3.744300	−3.548300	−1.536600
C	3.305800	−1.462200	−1.739900
H	3.800700	−1.449100	−2.713000
O	3.087200	−2.803300	0.262800
O	1.946800	−1.731900	−2.071400
C	1.183300	−2.339600	−1.114700
C	−0.472200	−3.459100	0.824900
C	−0.191900	−2.383900	−1.318800
C	1.734900	−2.870300	0.049500
C	0.917500	−3.445200	1.025600

C	−1.033800	−2.915000	−0.351100
H	−0.573100	−1.959800	−2.242200
C	1.541600	−4.006900	2.278600
H	1.374700	−5.087700	2.351300
H	1.106700	−3.548700	3.172500
H	2.617100	−3.828100	2.290100
C	−1.377300	−4.025900	1.834300
H	−0.975600	−4.716700	2.570000
C	−2.670100	−3.683100	1.848700
H	−3.338800	−4.098400	2.597900
C	−3.175000	−2.675200	0.843000
H	−2.696300	−1.730300	1.144300
C	−2.552100	−2.982500	−0.552700
C	−4.704100	−2.413400	0.882700
C	−3.041000	−1.932400	−1.565300
H	−2.745500	−2.237100	−2.577300
H	−2.535200	−0.977800	−1.374100
C	−4.558000	−1.712200	−1.540700
H	−5.079800	−2.613900	−1.884700
H	−4.788500	−0.932200	−2.274700
C	−5.075200	−1.284900	−0.150100
C	−2.851400	−4.391800	−1.110700
H	−2.160100	−4.592900	−1.935600
H	−3.865300	−4.479900	−1.506500
H	−2.706300	−5.165000	−0.351300
C	−5.098100	−1.889200	2.276200
H	−4.546100	−0.964700	2.482200
H	−4.795700	−2.598600	3.055900
C	−6.629900	−1.128500	−0.167500
H	−6.979200	−2.102600	−0.535600
C	−6.608200	−1.665500	2.402800
H	−6.811000	−1.108300	3.326200
H	−7.083500	−2.640900	2.551900
C	−7.323800	−0.935000	1.229200
C	−4.382800	0.052600	0.187900
H	−4.385400	0.684300	−0.703700
H	−3.335900	−0.065500	0.474400
H	−4.870100	0.604600	0.988900
C	−5.466000	−3.730800	0.617700
H	−5.454400	−4.026600	−0.431100
H	−6.514800	−3.674800	0.913700
H	−5.013700	−4.549500	1.187800
C	−7.476300	0.556300	1.610100
H	−6.506400	1.006400	1.843900
H	−8.057700	0.600000	2.540400
C	−7.208700	−0.132100	−1.213800
H	−6.600200	−0.132500	−2.122500
H	−8.182700	−0.534400	−1.521500
C	−8.179100	1.403300	0.554500
H	−8.223700	2.445800	0.884800
H	−9.214700	1.062800	0.433900
C	−7.498700	1.335700	−0.814700

C	−8.737300	−1.543600	1.136900
H	−9.345300	−1.091100	0.348200
H	−9.270300	−1.412400	2.086300
H	−8.679200	−2.618700	0.931500
C	−8.426200	1.946700	−1.884800
H	−7.976200	1.883400	−2.878900
H	−8.637400	2.998700	−1.663900
H	−9.377100	1.404400	−1.895000
C	−6.259800	2.223200	−0.804900
O	−5.906900	2.935700	0.103200
O	−5.610900	2.190400	−1.988700
C	−4.473000	3.046400	−2.071800
H	−4.764100	4.084800	−1.898900
H	−4.075300	2.917400	−3.077500
H	−3.726100	2.761500	−1.324700
C	5.348700	−4.950900	−0.008100
H	5.303200	−5.382300	−1.015300
H	4.330000	−4.904500	0.384400
H	5.937200	−5.626100	0.614200
C	5.534800	1.319900	−0.282500
H	6.590300	1.560600	−0.453100
H	5.433200	1.114200	0.789900
C	2.710400	1.098900	−1.298100
C	4.706900	2.548900	−0.655200
H	4.906700	2.844700	−1.693600
H	5.051400	3.376900	−0.026900
C	3.199500	2.335100	−0.436100
C	5.722900	0.240400	−2.514400
H	5.470800	−0.580200	−3.190100
H	6.815500	0.262300	−2.444100
H	5.409000	1.174100	−2.980500
C	2.415500	3.601800	−0.918600
H	2.675900	3.662400	−1.983600
C	1.242500	0.862500	−0.875200
H	0.820400	0.025300	−1.426100
H	1.207300	0.581600	0.183800
C	0.853500	3.497800	−0.872200
C	0.330100	2.064600	−1.165100
H	0.049400	2.015100	−2.223400
H	−0.613100	1.920500	−0.620200
C	2.722700	1.372800	−2.818000
H	2.621200	0.430900	−3.367000
H	3.629800	1.870500	−3.163900
H	1.886400	2.003600	−3.125500
C	3.012700	2.056000	1.074500
H	3.234100	1.020200	1.339500
H	2.004200	2.258900	1.430200
H	3.705800	2.676000	1.647400
C	2.883700	4.980800	−0.378200
H	3.971300	5.013500	−0.272800
H	2.644900	5.718300	−1.155000
C	0.266200	3.984700	0.474000

H	0.474800	3.271600	1.276200
H	−0.827100	3.998400	0.369900
C	2.256800	5.537300	0.918900
C	0.732700	5.376500	0.895000
H	0.315900	5.631700	1.874300
H	0.340100	6.119700	0.190000
C	0.279100	4.403000	−1.981100
H	0.646600	4.086600	−2.964000
H	0.538800	5.457800	−1.854500
H	−0.815600	4.331700	−1.994800
C	2.614900	7.032800	1.024700
H	2.205700	7.572000	0.164300
H	3.699500	7.167400	1.046600
H	2.191500	7.471400	1.935200
C	2.927500	4.910100	2.134800
O	4.125900	4.899000	2.303100
O	2.066400	4.416300	3.037700
C	2.667900	3.854700	4.203700
H	3.307900	4.591100	4.693900
H	3.269800	2.982700	3.935100
H	1.842400	3.563700	4.851300

M06-2X/6-31G(d) Free Energy = −2935.117194

cangorosin Aβ

H	4.068200	3.062300	2.923900
C	3.668800	3.483100	2.004100
C	2.684400	4.633600	−0.344400
C	3.322300	2.644800	0.935300
C	3.509200	4.850100	1.917800
C	3.026700	5.430300	0.742300
C	2.788300	3.229500	−0.224700
O	3.819600	5.737300	2.916900
H	4.142400	5.256300	3.690900
O	2.910900	6.784400	0.658800
H	3.216700	7.155100	1.502000
C	3.545800	1.132300	1.113300
C	3.490800	0.459200	−0.294400
H	4.288700	0.969100	−0.851800
C	2.386300	2.369700	−1.414600
C	2.240200	0.893100	−1.057500
O	1.206200	2.819800	−2.072200
O	1.078600	0.668600	−0.267100
C	−0.028600	1.404900	−0.575500
C	−2.346900	2.813900	−1.187600
C	−1.228800	1.060500	0.037200
C	0.025200	2.473400	−1.468300
C	−1.135500	3.175700	−1.799300
C	−2.396600	1.752100	−0.259000
H	−1.213000	0.229500	0.735100
C	−1.071500	4.280800	−2.824400
H	−1.253500	5.260800	−2.367600
H	−1.830500	4.135100	−3.599000

H	−0.092300	4.308900	−3.303400
C	−3.590000	3.533600	−1.496300
H	−3.524100	4.510600	−1.966300
C	−4.779300	2.992500	−1.210600
H	−5.693200	3.530600	−1.446900
C	−4.841600	1.611800	−0.601200
H	−4.528300	0.937100	−1.414300
C	−3.716900	1.465000	0.466500
C	−6.263600	1.152200	−0.178700
C	−3.763300	0.041900	1.045700
H	−3.077200	−0.027800	1.899400
H	−3.394400	−0.669800	0.296600
C	−5.163000	−0.385100	1.503800
H	−5.484600	0.224500	2.357600
H	−5.088500	−1.412400	1.878000
C	−6.209400	−0.323200	0.369400
C	−3.780600	2.479000	1.631400
H	−2.829000	2.446200	2.171700
H	−4.572300	2.249100	2.346900
H	−3.927400	3.499800	1.268300
C	−7.182900	1.131400	−1.414100
H	−6.762000	0.450300	−2.162500
H	−7.212100	2.118100	−1.891700
C	−7.629200	−0.686900	0.911000
H	−7.779700	0.031200	1.727900
C	−8.623000	0.739200	−1.062100
H	−9.166100	0.514300	−1.988600
H	−9.122700	1.622900	−0.651100
C	−8.828500	−0.450900	−0.077900
C	−5.746000	−1.320400	−0.713600
H	−5.390600	−2.232100	−0.226700
H	−4.910600	−0.945100	−1.308000
H	−6.532400	−1.602100	−1.411100
C	−6.847300	2.143000	0.854200
H	−6.462800	1.982900	1.861500
H	−7.934700	2.079800	0.922600
H	−6.601500	3.172700	0.574300
C	−9.164700	−1.710400	−0.910700
H	−8.384200	−1.914700	−1.650000
H	−10.072100	−1.489900	−1.488200
C	−7.765200	−2.061200	1.626900
H	−6.842800	−2.307900	2.159700
H	−8.523500	−1.932800	2.410000
C	−9.405700	−2.965100	−0.079000
H	−9.610900	−3.814400	−0.738400
H	−10.293200	−2.829200	0.551300
C	−8.226800	−3.307500	0.834400
C	−10.067800	−0.106400	0.772500
H	−10.311400	−0.884500	1.501900
H	−10.945000	0.036000	0.129800
H	−9.904700	0.824800	1.327400
C	−8.654100	−4.388300	1.848000

H	−7.838300	−4.629900	2.534300
H	−8.959600	−5.306200	1.333400
H	−9.507300	−4.026000	2.430600
C	−7.119700	−3.950200	0.008800
O	−7.183400	−4.232900	−1.162900
O	−6.044600	−4.255600	0.766300
C	−4.996700	−4.930400	0.072600
H	−5.356900	−5.880100	−0.329000
H	−4.211000	−5.095200	0.808400
H	−4.629500	−4.316000	−0.753800
C	2.252800	5.344900	−1.603300
H	1.165800	5.468800	−1.635800
H	2.543400	4.795300	−2.500600
H	2.703100	6.339100	−1.632400
C	4.968800	0.861100	1.656800
H	5.049000	1.224800	2.687600
H	5.696200	1.437300	1.071300
C	3.866300	−1.053000	−0.365900
C	5.352100	−0.624300	1.643000
H	4.702400	−1.185800	2.326800
H	6.365600	−0.706900	2.051100
C	5.314200	−1.247400	0.231800
C	2.538000	0.663500	2.190800
H	1.508500	0.873500	1.909000
H	2.753400	1.213200	3.113800
H	2.623600	−0.396700	2.425100
C	5.602200	−2.781600	0.305900
H	4.813000	−3.153800	0.971800
C	3.940000	−1.511200	−1.836300
H	2.965300	−1.398500	−2.323700
H	4.632600	−0.862800	−2.386500
C	5.425900	−3.574300	−1.037200
C	4.331200	−2.990300	−1.980000
H	3.420700	−3.585900	−1.856400
H	4.640800	−3.168100	−3.017500
C	2.798200	−1.917200	0.344800
H	1.805300	−1.485500	0.193700
H	2.960400	−2.003300	1.419800
H	2.778900	−2.937400	−0.043900
C	6.387900	−0.520400	−0.604400
H	6.103000	0.494700	−0.887600
H	6.650800	−1.042800	−1.522200
H	7.294400	−0.422600	−0.003000
C	6.903300	−3.215400	1.039600
H	7.122400	−2.537100	1.868900
H	6.686300	−4.182800	1.511000
C	6.746600	−3.692300	−1.835100
H	7.043300	−2.728700	−2.259800
H	6.554800	−4.350300	−2.692800
C	8.203100	−3.455100	0.236600
C	7.914200	−4.257300	−1.033700
H	8.813400	−4.283400	−1.657100

H	7.700100	−5.291800	−0.738900
C	4.971600	−5.003600	−0.677500
H	4.005500	−4.980700	−0.160700
H	5.680800	−5.523500	−0.026900
H	4.851400	−5.605200	−1.586500
C	9.183500	−4.237800	1.133200
H	8.754800	−5.215600	1.375300
H	9.380300	−3.700800	2.064800
H	10.136400	−4.402700	0.618500
C	8.935200	−2.171500	−0.131800
O	9.321200	−1.865900	−1.233400
O	9.184600	−1.410900	0.956500
H	2.119000	0.347500	−2.000800
H	3.156200	2.442200	−2.193200
C	9.941500	−0.229300	0.701200
H	10.079100	0.250500	1.669100
H	9.399600	0.430600	0.018000
H	10.905000	−0.483500	0.253900

M06-2X/6-31G(d) Free Energy = −2935.113727

isocangorosin A

H	−5.598300	4.468200	0.664700
C	−4.555300	4.567400	0.375100
C	−1.875800	4.918000	−0.346700
C	−3.910700	3.548000	−0.341500
C	−3.881600	5.720600	0.724800
C	−2.541500	5.905900	0.365000
C	−2.576000	3.746400	−0.702300
O	−4.435300	6.758900	1.428700
H	−5.346000	6.541200	1.669400
O	−1.901400	7.053700	0.720000
H	−2.528700	7.592800	1.227400
C	−4.713000	2.310500	−0.780000
C	−3.715000	1.117000	−1.030100
H	−3.134900	1.038800	−0.101900
C	−1.812800	2.678100	−1.435100
H	−1.173600	3.113400	−2.216700
C	−2.708500	1.627400	−2.084800
H	−3.240700	2.094600	−2.915700
O	−1.885000	0.662700	−2.733300
O	−0.959300	2.049000	−0.471100
C	−0.280400	0.968000	−0.960300
C	1.132900	−1.232300	−1.903600
C	0.864500	0.545400	−0.293900
C	−0.734600	0.286000	−2.087700
C	−0.027500	−0.816000	−2.576600
C	1.582900	−0.551300	−0.751200
H	1.163200	1.103900	0.587100
C	−0.532000	−1.537800	−3.801900
H	−0.879600	−2.547500	−3.550200
H	0.259000	−1.640600	−4.550800
H	−1.365800	−0.999400	−4.253600

C	1.897500	−2.396500	−2.371300
H	1.424300	−3.090000	−3.060400
C	3.155600	−2.594500	−1.963300
H	3.713200	−3.457100	−2.317900
C	3.809100	−1.589900	−1.044100
H	3.968000	−0.701200	−1.675700
C	2.779200	−1.117500	0.024200
C	5.209500	−2.007400	−0.517100
C	3.441500	−0.060400	0.922600
H	2.779200	0.168900	1.766900
H	3.557200	0.875100	0.360900
C	4.805500	−0.496400	1.472300
H	4.682200	−1.341000	2.161600
H	5.197900	0.330400	2.074600
C	5.817100	−0.850000	0.359900
C	2.199100	−2.237800	0.918100
H	1.317300	−1.846600	1.435700
H	2.902700	−2.575800	1.681000
H	1.887500	−3.103500	0.327600
C	6.168600	−2.211100	−1.705800
H	6.237300	−1.277600	−2.275900
H	5.770000	−2.957800	−2.402700
C	7.154400	−1.367800	0.980700
H	6.832800	−2.208700	1.609100
C	7.556300	−2.683900	−1.256600
H	8.252100	−2.598300	−2.100800
H	7.496800	−3.757800	−1.049000
C	8.196100	−1.975100	−0.026600
C	6.036800	0.437200	−0.462900
H	6.103300	1.287300	0.220500
H	5.210400	0.654500	−1.142200
H	6.944200	0.422800	−1.063500
C	5.098200	−3.339400	0.259700
H	4.714100	−3.211900	1.271700
H	6.059200	−3.847300	0.354200
H	4.420500	−4.026300	−0.258600
C	9.202700	−0.920700	−0.544900
H	8.719900	−0.213500	−1.226500
H	9.952800	−1.449900	−1.147200
C	7.854700	−0.425100	1.999700
H	7.113300	0.137900	2.573300
H	8.354200	−1.072200	2.732000
C	9.919000	−0.145200	0.556600
H	10.586500	0.599800	0.112200
H	10.548300	−0.827500	1.140700
C	8.955600	0.550200	1.522000
C	8.997400	−3.053700	0.730500
H	9.506700	−2.661600	1.615800
H	9.758400	−3.493900	0.074900
H	8.335200	−3.861200	1.063700
C	9.731800	1.041600	2.760700
H	9.062400	1.518300	3.481500

H	10.506000	1.762300	2.474600
H	10.221900	0.191300	3.245800
C	8.409800	1.811900	0.865600
O	8.742600	2.251600	−0.208000
O	7.538100	2.456300	1.670600
C	7.037700	3.685800	1.149100
H	7.856700	4.387500	0.976400
H	6.351300	4.071100	1.901700
H	6.516000	3.514800	0.203600
C	−0.429900	5.129000	−0.719200
H	−0.326200	5.346800	−1.789000
H	0.161000	4.237100	−0.498400
H	−0.016000	5.973900	−0.167800
C	−5.707400	1.892300	0.320800
H	−6.491700	2.652600	0.415400
H	−5.189400	1.859900	1.287000
C	−4.369400	−0.288600	−1.262100
C	−6.382400	0.548400	0.048700
H	−7.011000	0.609700	−0.849200
H	−7.062600	0.349100	0.883600
C	−5.374800	−0.607800	−0.082400
C	−5.557200	2.772800	−1.996400
H	−4.967300	2.969300	−2.894600
H	−6.045100	3.715900	−1.729500
H	−6.346100	2.066900	−2.256400
C	−6.136400	−1.930000	−0.423700
H	−6.661500	−1.681400	−1.355700
C	−3.310300	−1.412600	−1.215400
H	−2.566000	−1.261800	−1.993000
H	−2.771000	−1.373000	−0.261800
C	−5.245300	−3.174000	−0.762200
C	−3.910500	−2.803500	−1.463200
H	−4.044100	−2.914800	−2.545500
H	−3.158700	−3.557500	−1.195700
C	−5.029900	−0.328100	−2.656500
H	−4.324500	0.050300	−3.404700
H	−5.943200	0.262900	−2.724600
H	−5.293400	−1.342600	−2.960200
C	−4.635200	−0.704100	1.269400
H	−3.869200	0.064200	1.391400
H	−4.147300	−1.662600	1.430100
H	−5.353300	−0.554600	2.078700
C	−7.301100	−2.336700	0.523800
H	−7.832300	−1.455500	0.893100
H	−8.031900	−2.869500	−0.098200
C	−4.925500	−4.033800	0.484000
H	−4.228700	−3.519300	1.152800
H	−4.396600	−4.931300	0.137200
C	−7.031000	−3.287300	1.712100
C	−6.154800	−4.465600	1.278500
H	−5.846100	−5.031000	2.163300
H	−6.772800	−5.137000	0.670300

C	−6.028100	−4.059400	−1.752400
H	−6.239400	−3.507900	−2.676000
H	−6.984700	−4.408000	−1.352500
H	−5.436700	−4.943200	−2.019700
C	−8.386900	−3.814300	2.224800
H	−8.880600	−4.383800	1.430900
H	−9.041600	−2.992200	2.525800
H	−8.246000	−4.480100	3.083400
C	−6.394500	−2.595500	2.910300
O	−5.415200	−2.969300	3.507900
O	−7.111600	−1.519600	3.303700
C	−6.622900	−0.872000	4.476400
H	−5.614600	−0.484300	4.306900
H	−6.593200	−1.572400	5.313900
H	−7.317900	−0.058000	4.678400

M06-2X/6-31G(d) Free Energy = −2935.117707

isocangorosin Aβ

H	2.690800	2.848700	2.489700
C	2.096900	2.790500	1.581100
C	0.546000	2.708300	−0.744600
C	2.318700	1.761500	0.656000
C	1.121200	3.741000	1.365000
C	0.348100	3.707900	0.201500
C	1.511800	1.707700	−0.491100
O	0.839600	4.777900	2.217400
H	1.390200	4.707300	3.008900
O	−0.592000	4.671100	−0.006300
H	−0.558600	5.277300	0.751000
C	3.404300	0.725000	0.985800
C	3.789800	−0.024600	−0.330600
H	4.102000	0.785100	−1.005300
C	1.715300	0.616500	−1.532000
C	2.537100	−0.564400	−1.019600
O	1.795700	−1.362500	−0.103800
O	0.497800	0.130300	−2.088100
C	−0.204200	−0.707200	−1.266600
C	−1.677500	−2.393900	0.379700
C	−1.575100	−0.830400	−1.456200
C	0.441800	−1.450000	−0.281000
C	−0.287100	−2.295100	0.559100
C	−2.327400	−1.666700	−0.640700
H	−2.022800	−0.252900	−2.258700
C	0.426700	−3.075200	1.635800
H	0.301000	−4.153900	1.490800
H	0.029900	−2.829900	2.626600
H	1.495300	−2.856900	1.632500
C	−2.491300	−3.252900	1.250500
H	−1.996200	−4.015200	1.845000
C	−3.816500	−3.086100	1.325700
H	−4.408000	−3.718300	1.982400
C	−4.477900	−1.985400	0.529700

H	−4.160100	−1.055200	1.027600
C	−3.823200	−1.903100	−0.880800
C	−6.030600	−1.991600	0.572700
C	−4.471700	−0.754400	−1.670100
H	−4.125100	−0.785200	−2.710800
H	−4.133200	0.206000	−1.261900
C	−6.004100	−0.797000	−1.659700
H	−6.363600	−1.681600	−2.200800
H	−6.361700	0.069800	−2.226300
C	−6.593100	−0.760200	−0.232700
C	−3.910700	−3.196200	−1.724100
H	−3.205000	−3.114300	−2.557000
H	−4.902600	−3.354200	−2.151700
H	−3.640800	−4.078700	−1.138200
C	−6.506100	−1.823900	2.028200
H	−6.111000	−0.883500	2.428600
H	−6.092200	−2.615600	2.664000
C	−8.149500	−0.888200	−0.280500
H	−8.308600	−1.826500	−0.828100
C	−8.033300	−1.874900	2.151300
H	−8.321300	−1.526300	3.151100
H	−8.338800	−2.926400	2.121200
C	−8.869900	−1.084400	1.101800
C	−6.153300	0.580900	0.391500
H	−6.261500	1.370400	−0.356100
H	−5.104200	0.586900	0.694300
H	−6.736700	0.868300	1.264000
C	−6.560600	−3.339800	0.033800
H	−6.542300	−3.400400	−1.054200
H	−7.590300	−3.534000	0.338700
H	−5.950600	−4.166200	0.413700
C	−9.303300	0.258100	1.736300
H	−8.437500	0.833300	2.078800
H	−9.883100	0.023600	2.638700
C	−8.905400	0.153900	−1.153200
H	−8.307500	0.429500	−2.026100
H	−9.785900	−0.360500	−1.560000
C	−10.152700	1.136000	0.822600
H	−10.394500	2.074900	1.330500
H	−11.104400	0.636100	0.605100
C	−9.466700	1.445200	−0.510200
C	−10.143500	−1.915900	0.848300
H	−10.820200	−1.449100	0.126900
H	−10.698700	−2.057800	1.783500
H	−9.883800	−2.907400	0.459700
C	−10.486500	2.063300	−1.487600
H	−10.026400	2.272200	−2.456900
H	−10.894400	2.997700	−1.086700
H	−11.316900	1.365600	−1.636200
C	−8.410700	2.522500	−0.295700
O	−8.200500	3.113400	0.735500
O	−7.758400	2.815000	−1.440800

C	−6.802600	3.868600	−1.335100
H	−7.288800	4.791000	−1.009700
H	−6.377100	3.986000	−2.330600
H	−6.024200	3.607200	−0.613000
C	−0.279600	2.777800	−2.006200
H	−1.167600	2.141600	−1.932000
H	0.284700	2.440600	−2.878000
H	−0.611000	3.804600	−2.170900
C	4.691400	1.429600	1.470500
H	4.516000	1.894200	2.447000
H	4.939700	2.248100	0.782900
C	5.021100	−0.985400	−0.271700
C	5.885500	0.480400	1.603100
H	5.674300	−0.279900	2.367000
H	6.733300	1.064300	1.978300
C	6.272100	−0.187500	0.268200
C	2.841300	−0.109100	2.166200
H	1.812200	−0.418000	1.987000
H	2.846500	0.524200	3.061200
H	3.426000	−0.999300	2.394600
C	7.437400	−1.204400	0.483100
H	7.030000	−1.901700	1.226000
C	5.393200	−1.457400	−1.691900
H	4.563100	−2.010600	−2.145000
H	5.564400	−0.585100	−2.333900
C	7.810000	−2.086800	−0.760200
C	6.608400	−2.400200	−1.703200
H	6.246300	−3.407700	−1.473100
H	6.986700	−2.466700	−2.730800
C	4.705900	−2.241900	0.571100
H	3.686200	−2.582800	0.371800
H	4.797600	−2.072700	1.644800
H	5.380200	−3.066400	0.333900
C	6.689200	0.943200	−0.695700
H	5.843400	1.506000	−1.095600
H	7.263400	0.591600	−1.550500
H	7.304000	1.663900	−0.152300
C	8.715800	−0.666900	1.186700
H	8.456300	0.088300	1.933400
H	9.129300	−1.506900	1.759900
C	8.948400	−1.469200	−1.609400
H	8.604600	−0.584600	−2.153800
H	9.220900	−2.204600	−2.377800
C	9.903700	−0.148800	0.344500
C	10.198300	−1.103900	−0.814300
H	10.940500	−0.650300	−1.478700
H	10.654200	−2.010000	−0.397200
C	8.314100	−3.444400	−0.228700
H	7.521100	−3.959400	0.325100
H	9.171100	−3.343600	0.443400
H	8.616700	−4.092100	−1.060000
C	11.137800	−0.051400	1.264100

H	11.392700	−1.047500	1.640000
H	10.943300	0.605500	2.116000
H	12.003200	0.336300	0.715200
C	9.698200	1.258500	−0.199800
O	9.845500	1.603500	−1.346700
O	9.396600	2.134300	0.783500
H	2.248300	1.031700	−2.396700
H	2.777300	−1.185100	−1.890700
C	9.275800	3.491000	0.360500
H	9.025800	4.060600	1.254400
H	8.488400	3.589100	−0.391600
H	10.217100	3.840500	−0.069700

M06-2X/6-31G(d) Free Energy = −2935.113875

isoxuxurine Aα

C	4.160400	−3.543800	−0.054700
C	1.806500	−4.547500	−0.040800
C	2.347500	−2.532300	−1.359100
C	1.592900	−3.850100	−1.388200
C	3.707600	−2.517500	−0.817000
C	3.304500	−4.672900	0.290400
H	5.174200	−3.591200	0.326200
O	3.681600	−5.684500	0.851100
C	1.818200	−1.385600	−1.821800
H	0.797300	−1.383600	−2.193600
C	4.631000	−1.392900	−1.284500
C	3.867700	−0.071600	−1.504200
C	2.552500	−0.132300	−1.781100
H	1.984600	0.767700	−1.983800
C	5.148200	−1.913100	−2.663600
H	5.502100	−2.942300	−2.543700
H	5.984300	−1.316300	−3.029100
H	4.348800	−1.899500	−3.410300
C	5.829200	−1.246100	−0.316800
H	6.560000	−2.033000	−0.537600
H	5.479700	−1.433600	0.703100
C	6.533500	0.111400	−0.336700
H	7.132800	0.233300	−1.246000
H	7.246400	0.112400	0.494000
C	4.626700	1.260500	−1.472400
C	5.543100	1.284400	−0.194800
C	3.675200	2.470700	−1.408900
H	3.000900	2.461700	−2.272100
H	3.041100	2.403700	−0.516100
C	6.332300	2.631800	−0.114300
H	7.039800	2.579900	−0.953900
C	5.516900	3.949400	−0.347800
C	4.438900	3.794200	−1.436300
H	4.899000	3.922800	−2.422200
H	3.727300	4.623900	−1.338100
C	5.427500	1.392500	−2.792400
H	6.082400	0.548400	−2.990800

H	6.053400	2.286800	−2.807800
H	4.722200	1.462400	−3.627700
C	4.697300	1.027900	1.069900
H	3.992000	0.203900	0.926900
H	4.106900	1.892500	1.372500
H	5.347300	0.757900	1.909600
C	4.834700	4.472900	0.941300
H	4.015200	3.815200	1.253300
H	4.404900	5.461200	0.748400
C	7.230100	2.772700	1.140400
H	7.687600	1.811300	1.395200
H	8.070700	3.430600	0.884500
C	6.586800	3.349000	2.420200
C	5.822000	4.613300	2.079500
C	6.487700	5.051400	−0.807700
H	7.037100	4.737500	−1.702600
H	7.216300	5.316500	−0.034500
H	5.931300	5.962400	−1.054600
C	7.629100	3.598500	3.502100
H	8.158600	2.673500	3.749100
H	7.165700	3.992500	4.409100
H	8.359900	4.338000	3.160500
O	1.205200	−5.783100	−0.040800
H	1.725500	−6.326800	0.581800
C	2.055200	−4.718000	−2.551900
H	3.136300	−4.880600	−2.506300
H	1.815800	−4.208300	−3.488400
H	5.837400	2.637200	2.790700
O	6.009300	5.666700	2.647600
C	0.014400	−3.283600	0.792900
C	−0.517200	−3.247900	−0.498200
C	−1.817300	−2.816800	−0.708800
H	−2.181700	−2.836100	−1.729900
C	−2.606900	−2.387500	0.355700
C	−2.063300	−2.422700	1.652300
C	−0.744800	−2.875400	1.887200
C	−0.115600	−2.932700	3.256500
H	−0.654100	−3.632700	3.898700
H	−0.175500	−1.961800	3.753200
H	0.926500	−3.240000	3.178900
C	−4.074600	−2.027800	0.081100
C	−2.904500	−1.938900	2.774000
O	−2.616100	−2.083400	3.952400
C	−4.120500	−1.183900	2.414100
H	−4.537100	−0.641800	3.255100
C	−4.670700	−1.152200	1.193000
C	−4.197900	−1.357900	−1.313800
H	−4.165400	−2.137900	−2.083000
H	−3.318200	−0.728300	−1.482400
C	−5.918300	−0.298500	0.914000
C	−5.453400	−0.511400	−1.533100
H	−6.341300	−1.145200	−1.638800

H	−5.332100	−0.003700	−2.495600
C	−5.670200	0.517400	−0.406700
C	−4.812100	−3.398600	0.037800
H	−4.228300	−4.095000	−0.570900
H	−5.802900	−3.320000	−0.412600
H	−4.912600	−3.818400	1.042700
C	−6.203700	0.700200	2.051600
H	−5.336400	1.354800	2.202200
H	−6.350400	0.162300	2.994200
C	−6.913800	1.406300	−0.733800
H	−7.702100	0.681400	−0.981700
C	−7.465500	1.521400	1.789300
H	−8.341600	0.868100	1.864400
H	−7.588200	2.261000	2.590700
C	−7.501900	2.262400	0.439100
C	−7.144600	−1.240400	0.829300
H	−7.046900	−2.020900	0.079900
H	−8.065200	−0.699200	0.602300
H	−7.277200	−1.732800	1.798900
C	−4.372500	1.343500	−0.283400
H	−4.040800	1.674500	−1.273900
H	−3.559500	0.755100	0.151900
H	−4.485600	2.228100	0.342700
C	−6.786600	3.626500	0.610300
H	−5.768500	3.496400	0.994600
H	−7.334000	4.234300	1.338200
C	−6.768800	2.274900	−2.006700
H	−6.234300	1.726200	−2.788700
H	−7.772700	2.450300	−2.414400
C	−6.732100	4.401300	−0.688400
C	−6.102100	3.658900	−1.851800
H	−5.048100	3.515100	−1.579200
C	−8.974700	2.580200	0.124400
H	−9.086800	3.230700	−0.749600
H	−9.435600	3.097500	0.973300
H	−9.540800	1.659900	−0.058900
O	−7.188800	5.518400	−0.796700
C	−6.184700	4.465900	−3.140700
H	−5.708300	3.926900	−3.965200
H	−5.698300	5.437100	−3.025900
H	−7.229800	4.654200	−3.404800
O	1.305700	−3.710400	0.993900
O	0.199200	−3.645600	−1.589300
H	1.542500	−5.680400	−2.513000

M06-2X/6-31G(d) Free Energy = −2700.480231

isoxuxuarine Aβ

C	3.396400	3.356100	0.126300
C	0.977400	3.512600	−0.646400
C	2.250800	1.395400	−0.790300
C	1.253700	2.287300	−1.516600
C	3.322700	2.005500	−0.017200

C	2.296900	4.218700	−0.283500
H	4.220400	3.846600	0.632500
O	2.316300	5.435700	−0.287000
C	2.232800	0.058000	−0.965500
H	1.430400	−0.380500	−1.553700
C	4.243600	1.086300	0.795100
C	4.324700	−0.342500	0.247300
C	3.304000	−0.798600	−0.504200
H	3.286000	−1.822700	−0.856700
C	3.481800	1.029900	2.161500
H	3.305000	2.053100	2.506400
H	4.053400	0.502400	2.924400
H	2.513800	0.534600	2.040400
C	5.630500	1.753600	0.990600
H	5.558800	2.491200	1.797600
H	5.867000	2.319800	0.083400
C	6.801500	0.809300	1.282100
H	6.783800	0.468900	2.322500
H	7.718600	1.398500	1.178100
C	5.524500	−1.228700	0.600700
C	6.835400	−0.401400	0.329000
C	5.563900	−2.508400	−0.254500
H	4.644500	−3.085100	−0.103500
H	5.594300	−2.244700	−1.319200
C	8.101200	−1.270800	0.628500
H	8.086500	−1.397900	1.720400
C	8.120000	−2.727200	0.050600
C	6.741300	−3.407200	0.115600
H	6.584500	−3.813900	1.120500
H	6.754900	−4.279400	−0.550400
C	5.392100	−1.678100	2.077100
H	5.326200	−0.850700	2.778400
H	6.234600	−2.293700	2.397800
H	4.479700	−2.274900	2.185400
C	6.832900	0.150000	−1.111500
H	5.870100	0.599500	−1.372700
H	7.034800	−0.612700	−1.863600
H	7.593500	0.931500	−1.215500
C	8.621400	−2.787500	−1.413800
H	7.901300	−2.326000	−2.099300
H	8.737400	−3.833300	−1.716700
C	9.448200	−0.565000	0.335000
H	9.392000	0.495500	0.601400
H	10.205500	−0.989500	1.005900
C	10.013300	−0.668300	−1.098500
C	9.964200	−2.108900	−1.573200
C	9.096600	−3.574600	0.884700
H	8.821300	−3.553500	1.945300
H	10.132900	−3.232800	0.793400
H	9.069100	−4.617200	0.548800
C	11.423800	−0.099900	−1.182600
H	11.438100	0.947700	−0.867100

H	11.812300	−0.167500	−2.201100
H	12.100500	−0.667700	−0.536500
O	0.120200	4.376600	−1.281700
H	0.381700	5.269900	−0.984800
C	1.768300	2.684400	−2.893400
H	1.061300	3.372700	−3.359200
H	1.868200	1.784800	−3.505600
H	2.747800	3.165200	−2.814100
H	9.355200	−0.110700	−1.778700
O	10.932600	−2.684900	−2.017700
O	0.467600	3.078100	0.610200
C	−0.548800	2.156500	0.532500
C	−1.355200	1.945000	1.649700
C	−0.756700	1.427500	−0.641600
C	−2.386100	0.983100	1.554900
C	−1.089300	2.759900	2.890100
C	−1.783700	0.501000	−0.722100
O	0.028700	1.598200	−1.744700
C	−2.624700	0.274200	0.364500
C	−3.286600	0.728500	2.705900
H	−0.367300	3.546700	2.673800
H	−0.704600	2.127500	3.694100
H	−2.011700	3.202600	3.269800
H	−1.891700	−0.031400	−1.660600
C	−3.673400	−0.843200	0.265200
C	−4.555000	0.033500	2.410700
O	−3.053900	1.102900	3.845300
C	−4.800700	−0.663800	1.293600
C	−2.887300	−2.151000	0.573900
C	−4.215100	−0.924500	−1.185800
H	−5.283200	0.135500	3.206900
C	−6.162500	−1.341000	1.069100
H	−1.964000	−2.153600	−0.013200
H	−3.450500	−3.046400	0.305600
H	−2.623600	−2.204300	1.634200
H	−3.481900	−1.452700	−1.806200
H	−4.284600	0.088300	−1.595100
C	−5.572300	−1.611200	−1.347400
C	−6.637600	−1.030300	−0.396900
C	−7.236100	−0.819800	2.044400
C	−5.999600	−2.855400	1.348800
H	−5.485700	−2.694100	−1.201000
H	−5.877700	−1.473400	−2.389800
C	−8.018400	−1.710700	−0.667900
C	−6.676000	0.494500	−0.631700
H	−6.918000	−0.993600	3.077700
H	−7.345500	0.266000	1.932700
C	−8.577000	−1.528100	1.851400
H	−5.249900	−3.334700	0.725200
H	−6.931200	−3.404400	1.199800
H	−5.694700	−2.991000	2.392300
H	−7.794500	−2.786500	−0.652500

C	−9.133300	−1.510900	0.414500
C	−8.595800	−1.453900	−2.081600
H	−5.787700	0.988900	−0.227200
H	−7.537900	0.978800	−0.173900
H	−6.704700	0.709200	−1.705800
H	−8.484500	−2.566000	2.189800
H	−9.323600	−1.074400	2.515500
C	−9.937200	−0.200000	0.219800
C	−10.144900	−2.664700	0.295900
H	−7.796500	−1.456700	−2.829400
H	−9.238800	−2.303100	−2.346000
C	−9.437500	−0.175800	−2.284700
H	−9.322900	0.678000	0.449000
H	−10.788800	−0.190100	0.907900
C	−10.479400	−0.076100	−1.187500
H	−9.641300	−3.634300	0.382600
H	−10.692900	−2.647300	−0.651900
H	−10.886600	−2.595800	1.099600
C	−10.074900	−0.141000	−3.667100
H	−8.785900	0.699400	−2.161000
O	−11.661700	0.055900	−1.417300
H	−9.308800	−0.191300	−4.447000
H	−10.659600	0.771300	−3.804700
H	−10.756500	−0.987800	−3.793100

M06-2X/6-31G(d) Free Energy = −2700.477506

xuxuarine A*α*

C	3.289800	−2.924200	2.173100
C	0.955900	−3.676500	1.435100
C	2.636200	−2.885500	−0.189700
C	1.568900	−3.938000	0.055200
C	3.569000	−2.569700	0.894000
C	2.042500	−3.594600	2.522700
H	3.974100	−2.749700	2.995600
O	1.788900	−4.061200	3.617500
C	2.756700	−2.238900	−1.363400
H	2.047500	−2.445900	−2.160600
C	4.921200	−1.985700	0.489500
C	4.796600	−1.036100	−0.719100
C	3.773800	−1.222500	−1.573300
H	3.671400	−0.610000	−2.460900
C	5.738200	−3.246100	0.059700
H	5.638400	−4.013200	0.834400
H	6.799200	−3.015700	−0.043100
H	5.368300	−3.649600	−0.887100
C	5.615800	−1.333500	1.707300
H	6.057600	−2.123600	2.325900
H	4.856000	−0.849200	2.328500
C	6.688200	−0.296500	1.371700
H	7.591200	−0.773400	0.973700
H	6.987300	0.170400	2.315700
C	5.871900	0.028200	−0.969400

C	6.172900	0.769300	0.383600
C	5.422700	1.077900	−2.004900
H	5.173800	0.588100	−2.952400
H	4.506300	1.574200	−1.661700
C	7.267000	1.865300	0.172900
H	8.181000	1.295200	−0.043700
C	7.084100	2.823400	−1.053800
C	6.524800	2.096700	−2.291100
H	7.345200	1.595800	−2.816800
H	6.148000	2.851100	−2.993200
C	7.115200	−0.682900	−1.562300
H	7.464400	−1.519900	−0.962900
H	7.962500	−0.004900	−1.685300
H	6.856200	−1.077800	−2.550800
C	4.869000	1.351500	0.969900
H	4.039900	0.640600	0.900100
H	4.542900	2.260600	0.466200
H	5.006300	1.589400	2.030700
C	6.169400	4.034500	−0.744300
H	5.127100	3.721900	−0.614500
H	6.197000	4.734600	−1.585600
C	7.610600	2.683900	1.441700
H	7.600200	2.042800	2.328800
H	8.648100	3.030400	1.350900
C	6.749300	3.929700	1.742600
C	6.623900	4.779800	0.492300
C	8.458400	3.400200	−1.436000
H	9.178700	2.596800	−1.628200
H	8.871500	4.050600	−0.658000
H	8.371600	4.001900	−2.347700
C	7.316100	4.729700	2.907800
H	7.379400	4.109600	3.807200
H	6.694500	5.601300	3.124100
H	8.318600	5.095500	2.665400
O	0.032800	−4.644500	1.744700
H	0.046800	−4.717400	2.718600
C	2.152300	−5.342500	−0.039900
H	2.982700	−5.466800	0.661700
H	2.523500	−5.500800	−1.055400
H	5.728900	3.604900	1.986700
O	6.897700	5.959700	0.472900
H	1.374200	−6.072900	0.186900
O	0.545300	−3.881800	−0.926100
C	−0.334200	−2.834100	−0.816300
C	−1.146800	−2.524600	−1.905600
C	−0.420200	−2.098000	0.368000
C	−2.050700	−1.446000	−1.778600
C	−1.018300	−3.360900	−3.154300
C	−1.306800	−1.039100	0.473100
O	0.376400	−2.373700	1.443200
C	−2.142900	−0.701900	−0.589400
C	−2.959300	−1.089100	−2.895600

H	−0.356000	−4.206900	−2.973000
H	−0.629800	−2.763900	−3.983300
H	−1.993900	−3.726000	−3.480900
H	−1.316900	−0.499100	1.412900
C	−3.027000	0.546300	−0.462500
C	−4.101900	−0.215500	−2.564600
O	−2.836900	−1.517800	−4.033300
C	−4.204700	0.518800	−1.448500
C	−2.080700	1.735100	−0.806200
C	−3.499700	0.706300	1.006700
H	−4.868300	−0.221600	−3.331000
C	−5.453300	1.374200	−1.181900
H	−1.131500	1.593800	−0.280900
H	−2.490800	2.695100	−0.487600
H	−1.879800	1.775100	−1.880700
H	−2.680100	1.135700	1.594200
H	−3.687600	−0.285900	1.428700
C	−4.746100	1.568700	1.207800
C	−5.912200	1.128400	0.301500
C	−6.623500	1.004400	−2.114300
C	−5.097800	2.852900	−1.475100
H	−4.522300	2.629200	1.043300
H	−5.028500	1.481700	2.262100
C	−7.182800	1.978100	0.625000
C	−6.138000	−0.378900	0.544000
H	−6.324400	1.131000	−3.160100
H	−6.880700	−0.055000	−1.991500
C	−7.842500	1.893700	−1.873600
H	−4.232500	3.210200	−0.923800
H	−5.922500	3.530200	−1.244100
H	−4.870600	2.955200	−2.542000
H	−6.822300	3.016200	0.609000
C	−8.350100	1.935500	−0.419800
C	−7.740000	1.781700	2.056200
H	−5.338100	−0.983700	0.106100
H	−7.073300	−0.745600	0.122200
H	−6.152500	−0.589000	1.619300
H	−7.612500	2.912300	−2.205000
H	−8.666800	1.559500	−2.516300
C	−9.311900	0.738900	−0.205700
C	−9.196900	3.210100	−0.259600
H	−6.923400	1.668300	2.776000
H	−8.254500	2.706000	2.349400
C	−8.738800	0.626300	2.280500
H	−8.825800	−0.210700	−0.457600
H	−10.178400	0.847300	−0.866100
C	−9.820400	0.675800	1.218200
H	−8.573800	4.106200	−0.358900
H	−9.708000	3.254800	0.707900
H	−9.969200	3.247700	−1.036000
C	−9.329500	0.666900	3.683500
H	−8.216600	−0.328100	2.132300

O	−11.001700	0.699700	1.486400
H	−8.538600	0.602300	4.437000
H	−10.031400	−0.155700	3.837000
H	−9.880700	1.599800	3.836300

M06-2X/6-31G(d) Free Energy = −2700.479521

xuxuarine Aβ

C	2.905700	−2.731300	−2.164100
C	1.151300	−4.302000	−1.200100
C	2.173600	−2.497600	0.161900
C	1.685800	−3.940200	0.185700
C	2.798600	−1.969800	−1.042200
C	2.207400	−4.003400	−2.281000
H	3.435100	−2.398100	−3.049700
O	2.335000	−4.798900	−3.193500
C	2.167000	−1.753500	1.287300
H	1.709300	−2.166300	2.183700
C	3.150700	−0.478500	−1.075200
C	3.410600	0.121200	0.312200
C	2.849700	−0.480100	1.379100
H	2.948300	−0.058000	2.372000
C	1.819600	0.138000	−1.621900
H	1.537500	−0.394300	−2.535200
H	1.936000	1.195100	−1.861800
H	1.013800	0.025700	−0.890600
C	4.293000	−0.216900	−2.088400
H	3.874500	−0.212200	−3.101000
H	4.987900	−1.062400	−2.051200
C	5.092600	1.069900	−1.874700
H	4.522900	1.947500	−2.198600
H	5.962100	1.017400	−2.537800
C	4.238300	1.404900	0.451300
C	5.544500	1.243500	−0.410800
C	4.649800	1.673400	1.912100
H	3.757800	1.760400	2.541900
H	5.223200	0.822300	2.300500
C	6.441600	2.517400	−0.286600
H	5.869100	3.301000	−0.802400
C	6.688800	3.077800	1.155200
C	5.441100	2.973300	2.051300
H	4.777700	3.820800	1.846900
H	5.755500	3.095900	3.095500
C	3.364400	2.602800	0.004200
H	2.963700	2.496200	−1.000300
H	3.913900	3.545900	0.026800
H	2.513900	2.693200	0.688600
C	6.295600	−0.042500	−0.006700
H	5.615900	−0.894300	0.091800
H	6.822400	0.046400	0.943500
H	7.033200	−0.303500	−0.774000
C	7.869900	2.382900	1.877900
H	7.625200	1.344600	2.129600

H	8.081200	2.905900	2.816200
C	7.777900	2.435200	−1.065500
H	7.637400	1.909700	−2.015300
H	8.071800	3.455100	−1.343900
C	8.987200	1.802800	−0.343700
C	9.129900	2.407700	1.039600
C	7.057700	4.567800	1.044500
H	6.280100	5.121500	0.506200
H	8.010600	4.726500	0.528800
H	7.154100	5.005500	2.044300
C	10.265200	1.957500	−1.156900
H	10.153600	1.497600	−2.143500
H	11.111800	1.494800	−0.645200
H	10.503600	3.017000	−1.291000
H	8.784400	0.735800	−0.181900
O	10.162100	2.903700	1.434500
O	0.648400	−4.110800	1.141500
O	0.074700	−3.422600	−1.526700
C	−0.551400	−3.535600	0.804500
C	−0.828000	−3.206000	−0.525500
C	−2.035200	−2.616900	−0.864900
H	−2.195300	−2.398100	−1.914500
C	−2.990900	−2.336500	0.109300
C	−2.715400	−2.692000	1.441600
C	−1.487200	−3.288100	1.806900
C	−1.129900	−3.658100	3.224700
H	−1.784300	−4.450400	3.594600
H	−1.275100	−2.810800	3.898300
H	−0.095300	−3.997600	3.274700
C	−4.355700	−1.791600	−0.336100
C	−3.731400	−2.373000	2.472900
O	−3.687600	−2.789300	3.620800
C	−4.806600	−1.441300	2.078400
H	−5.345800	−1.043400	2.930400
C	−5.104000	−1.105100	0.816100
C	−4.170000	−0.847400	−1.553400
H	−4.035000	−1.456900	−2.454000
H	−3.238900	−0.285800	−1.425400
C	−6.220900	−0.092500	0.513300
C	−5.309400	0.141600	−1.803300
H	−6.194500	−0.369600	−2.198400
H	−4.975800	0.818400	−2.596800
C	−5.679400	0.940600	−0.539200
C	−5.147600	−3.049900	−0.799800
H	−4.503200	−3.656700	−1.442300
H	−6.036700	−2.790600	−1.376700
H	−5.448400	−3.656500	0.059200
C	−6.653000	0.685400	1.771000
H	−5.790900	1.210100	2.201300
H	−7.004100	−0.011700	2.539000
C	−6.784800	1.992400	−0.881800
H	−7.550400	1.412500	−1.415800

C	−7.793700	1.658500	1.477800
H	−8.704800	1.088300	1.265500
H	−8.019400	2.234700	2.384000
C	−7.537300	2.650100	0.325500
C	−7.463900	−0.873600	0.020500
H	−7.274300	−1.490300	−0.853900
H	−8.292500	−0.210200	−0.234100
H	−7.802900	−1.538200	0.822800
C	−4.385400	1.601000	−0.019200
H	−3.844600	2.071400	−0.848100
H	−3.712200	0.868400	0.435600
H	−4.569900	2.366900	0.733100
C	−6.798400	3.885000	0.903300
H	−5.884000	3.588900	1.430500
H	−7.445200	4.386200	1.630700
C	−6.345400	3.080300	−1.892400
H	−5.704600	2.648800	−2.667900
H	−7.240300	3.432200	−2.421800
C	−6.447800	4.889700	−0.172700
C	−5.640300	4.332100	−1.329200
H	−4.672800	4.033400	−0.904400
C	−8.906400	3.152700	−0.166100
H	−8.819100	3.962900	−0.897800
H	−9.488300	3.540200	0.677700
H	−9.477600	2.337700	−0.624800
O	−6.816200	6.043100	−0.127600
C	−5.416600	5.380000	−2.411500
H	−4.818900	4.967800	−3.230300
H	−4.905400	6.256700	−2.007600
H	−6.375100	5.718800	−2.816300
O	0.765900	−5.617900	−1.247400
H	0.951900	−5.912800	−2.160100
C	2.799400	−4.888000	0.610500
H	2.433600	−5.915300	0.574400
H	3.104400	−4.639200	1.629700
H	3.665400	−4.785400	−0.050200

M06-2X/6-31G(d) Free Energy = −2700.475154
